# The clinical and economic costs associated with regional disparities in varicella vaccine coverage in Italy over 50 years (2020–2070)

**DOI:** 10.1038/s41598-024-60649-8

**Published:** 2024-05-24

**Authors:** J. C. Lang, S. Samant, J. R. Cook, S. Ranjan, F. Senese, S. Starnino, S. Giuffrida, C. Azzari, V. Baldo, M. Pawaskar

**Affiliations:** 1 Biostatistics and Research Decision Sciences (BARDS) Health Economic and Decision Sciences (HEDS), Merck Canada Inc, Kirkland, QC Canada; 2 Center for Observational and Real-World Evidence, Merck & Co., Inc., Rahway, NJ USA; 3 CHEORS, North Wales, PA USA; 4 Market Access, MSD Italy, Rome, Italy; 5 Medical Affairs, MSD Italy, Rome, Italy; 6 LHU Reggio Calabria, Calabria, Italy; 7 Department of Health Sciences, University of Florence, and Meyer Children’s University Hospital, Florence, Italy; 8 Department of Cardiac Thoracic Vascular Sciences, Hygiene and Public Health Unit, and Public Health, University of Padua, Padua, Italy

**Keywords:** Health care economics, Preventive medicine, Dynamical systems, Numerical simulations

## Abstract

Italy implemented two-dose universal varicella vaccination (UVV) regionally from 2003 to 2013 and nationally from 2017 onwards. Our objective was to analyze regional disparities in varicella outcomes resulting from disparities in vaccine coverage rates (VCRs) projected over a 50-year time-horizon (2020–2070). A previously published dynamic transmission model was updated to quantify the potential public health impact of the UVV program in Italy at the national and regional levels. Four 2-dose vaccine strategies utilizing monovalent (V) and quadrivalent (MMRV) vaccines were evaluated for each region: (A) MMRV-MSD/MMRV-MSD, (B) MMRV-GSK/MMRV-GSK, (C) V-MSD/MMRV-MSD, and (D) V-GSK/MMRV-GSK. Costs were reported in 2022 Euros. Costs and quality-adjusted life-years (QALYs) were discounted 3% annually. Under strategy A, the three regions with the lowest first-dose VCR reported increased varicella cases (+ 34.3%), hospitalizations (+ 20.0%), QALYs lost (+ 5.9%), payer costs (+ 22.2%), and societal costs (+ 14.6%) over the 50-year time-horizon compared to the three regions with highest first-dose VCR. Regions with low first-dose VCR were more sensitive to changes in VCR than high first-dose VCR regions. Results with respect to second-dose VCR were qualitatively similar, although smaller in magnitude. Results were similar across all vaccine strategies.

## Introduction

Varicella-zoster virus (VZV) is a highly contagious infectious agent responsible for varicella (chicken pox) with primary infection and herpes zoster (shingles) on reactivation in later life^1,2^. While varicella is usually mild, it can lead to severe complications, hospitalizations, and death^1,3^. Globally, it is estimated that there are approximately 4,200,000 varicella-related complications requiring hospitalization and 4200 varicella-related deaths annually^1^.

Live-attenuated VZV vaccines are active immunizing agents that induce protection against VZV primary infection^4–6^. Varicella-containing vaccines may be available as monovalent formulations protecting against varicella only (e.g., VARIVAX® [V-MSD] by Merck Sharp & Dohme LLC, Rahway, NJ, USA [MSD] and VARILRIX® [V-GSK] by GlaxoSmithKline SA, Belgium [GSK]) or as quadrivalent formulations protecting against measles, mumps, and rubella (MMR) in addition to varicella (e.g., ProQuad® [MMRV-MSD] by MSD or PRIORIX TETRA® [MMRV-GSK] by GSK). Quadrivalent formulations of the vaccine have been shown to be immunologically non-inferior to monovalent vaccines^7–9^.

Italy implemented two-dose varicella vaccination programs in eight pilot regions (Sicily, Veneto, Puglia, Tuscany, Basilicata, Calabria, Sardinia, Friuli-Venezia-Giulia) between 2003 and 2013. Two-dose UVV (with first and second dose administered at 12–15 months and 5–6 years, respectively) was recommended to the national immunization program of Italy in 2015 and was made mandatory for all children in Italy in 2017^10,11^. Varicella vaccination in Italy has resulted in significant reductions in varicella cases and hospitalizations across regions and nationally^12,13^. For example, in Sicily, which introduced two-dose UVV in 2003, statutory varicella notifications were estimated to have decreased from 105.7 per 100,000 in 2003 to 9.2 per 100,000 in 2010^12^. On average, hospitalization rates decreased by 80.0% and 86.7% for < 1-year-olds and 1–5-year-olds, respectively, during the first five years following inclusion of varicella vaccines into regional immunization schedules^13^. A previous cost-effective modeling study by Azzari et al., estimated that two-dose UVV was cost-saving in Italy with respect to no vaccination from both a payer and societal perspective^14^. Studies elsewhere in Europe also show that UVV was associated with a decline in varicella cases, hospitalizations, and costs^1,3,7,11,15,16^.

Italian regions exhibit significant disparities in vaccine coverage rates (VCRs) for both MMR and varicella-containing vaccines. For example, in 2021 the average second-dose varicella VCR (for 5–6-year-olds) was 20.0% (range: 7.6–31.0%) in the five lowest-VCR regions versus 83.2% (range: 80.3–86.6%) in the five highest-VCR regions^17^. Whereas some of the current disparities in varicella VCR may be transient, e.g., due to the comparatively recent 2017 introduction of mandatory UVV in many Italian regions, persistent disparities in varicella VCRs should be expected, as evidenced by disparities observed in MMR VCRs. The average second-dose MMR VCR (for 5–6-year-olds) in 2021 was 76.5% (range: 71.3–79.3%) for the five lowest coverage regions and 91.9% (range: 89.9–92.6%) for the five highest coverage regions^17^. The primary objective of this study was to assess the impact of regional disparities in varicella VCRs on clinical and economic outcomes over a 50-year time-horizon using a dynamic transmission model in Italy. This study also compared the impact of different vaccination strategies on health outcomes.

We adapted a previously published national-level deterministic, age structured, dynamic transmission cost-effectiveness model for varicella vaccination in Italy to a multi-regional model by incorporating regional-level demographic and VCR data (2013–2019)^14^. Model compartments and corresponding transitions for each of the 21 Italian regions were parameterized to obtain results for each individual region. National results were estimated by aggregating regional results.

Following Azzari and colleagues^14^, our two-dose strategies utilizing monovalent (V) and quadrivalent (MMRV) varicella-containing vaccines were considered: (A) first dose MMRV-MSD, second dose MMRV-MSD; (B) first dose MMRV-GSK, second dose MMRV-GSK; (C) first dose V-MSD, second dose MMRV-MSD; and (D) first dose V-GSK, second dose MMRV-GSK. Consistent with the varicella vaccination schedule in Italy, infants were eligible for their first dose of varicella vaccine at 1 year and children were eligible for their second dose of varicella vaccine at 5 years^18^. Catchup vaccination was excluded from this analysis.

Outcomes of interest included varicella incidence and varicella-related hospitalization, mortality, quality-adjusted life-years (QALYs) lost (due to varicella infection and deaths), and varicella-associated costs from both payer (direct costs) and societal (direct and indirect costs) perspectives. Direct costs included costs for vaccination, outpatient visits, prescriptions, and hospitalization, whereas indirect costs included productivity loss in caregivers and adult patients. Based on Italian Medicines Agency guidelines, costs and QALYs were both discounted at 3% annually^19^, and costs were reported in 2022 Euros. A 50-year time-horizon (2020–2070) was employed and inputs from the pre-COVID period were used (e.g., VCR rates from 2013 to 2019). Thus, our model does not incorporate changes in VCRs or social mixing patterns due to the COVID-19 pandemic, or the decline in varicella- related hospitalization seen during the pandemic period^20–23^. Scenario analysis investigated the impact of varying first- and second-dose VCR (± 2.5, ± 5%, ± 10%).

A standard cost-effectiveness analysis comparing strategies A-D was performed, influential model parameters were identified through deterministic sensitivity analysis (DSA), and probabilistic sensitivity analysis (PSA) was conducted to evaluate robustness of model results to parameter uncertainty.

## Results

### Base case analysis

Strategy A dominated strategies B-D in all regions from both payer and societal perspectives, with the best clinical outcomes and lowest costs of all four strategies. Given the relatively straightforward nature of the cost-effectiveness and sensitivity analyses, we henceforth restrict our attention to analyzing the inter-regional differences in clinical and economic outcomes in Italy under strategy A. For additional details on the cost-effectiveness of different strategies, and for the results from the deterministic and probabilistic sensitivity analyses, we refer the reader to Sect. S2.2 of the supplementary materials.

**Figure 1:**
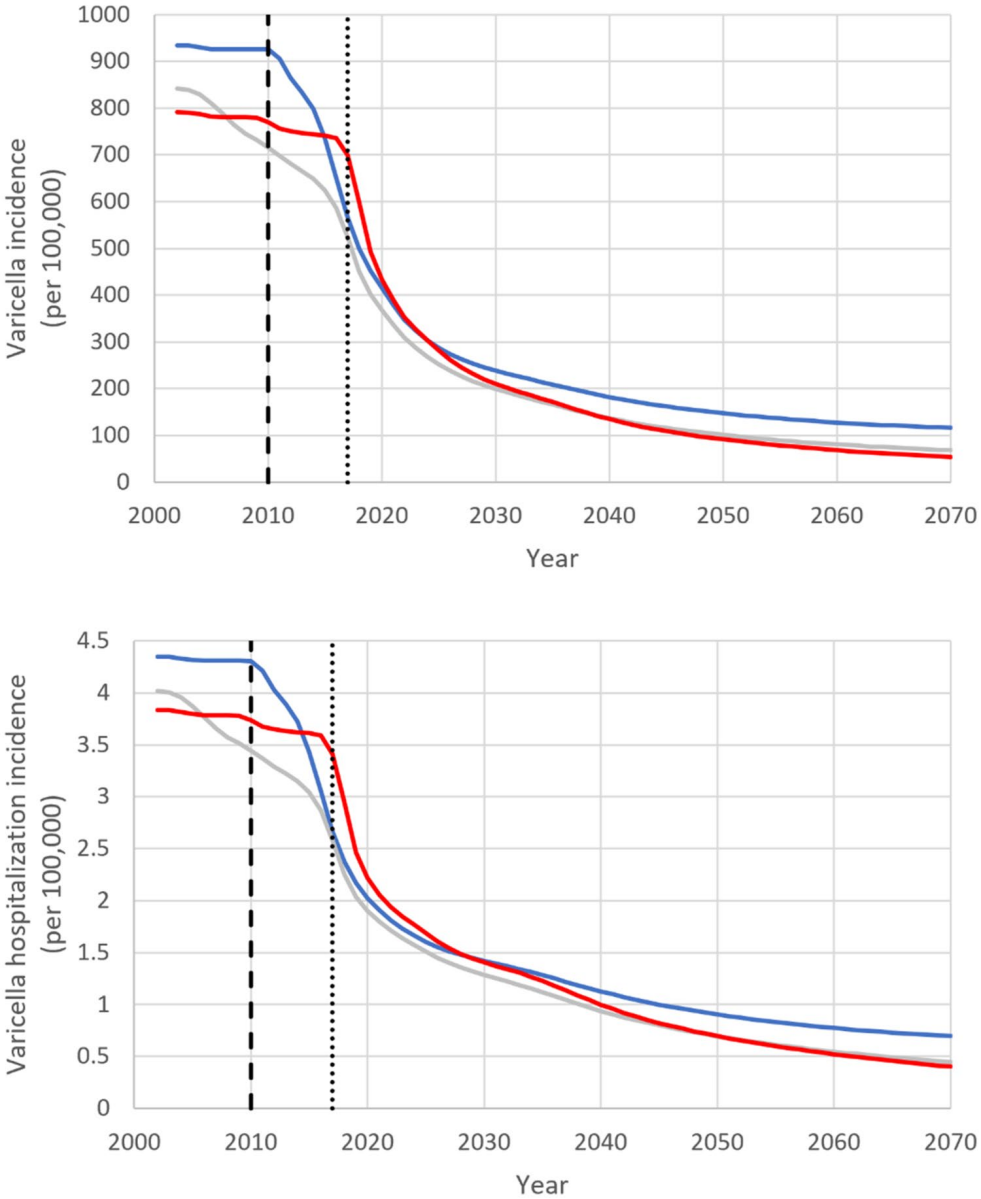
Varicella incidence under strategy A. Top row: All varicella incidence. Bottom row: Varicella hospitalization incidence. Grey line: National average. Blue line: Average of the three regions with lowest equilibrium first-dose VCR (vcr_equilibrium_1; Prov. Auton. Bolzano, Campania, Calabria). Red line: Average of three regions with highest equilibrium first-dose VCR (vcr_equilibrium_1; Piemonte, Umbria, Basilicata). Year of UVV adoption for low first-dose VCR: Calabria (2010), Campania (2017), Prov. Auton. Bolzano (2017). Year of adoption for high first-dose VCR: Basilicata (2010), Piemonte (2017), Umbria (2017). Dashed line: UVV adoption in 2010 Basilicata and Calabria. Dotted line: UVV adoption nationally.

**Figure 2:**
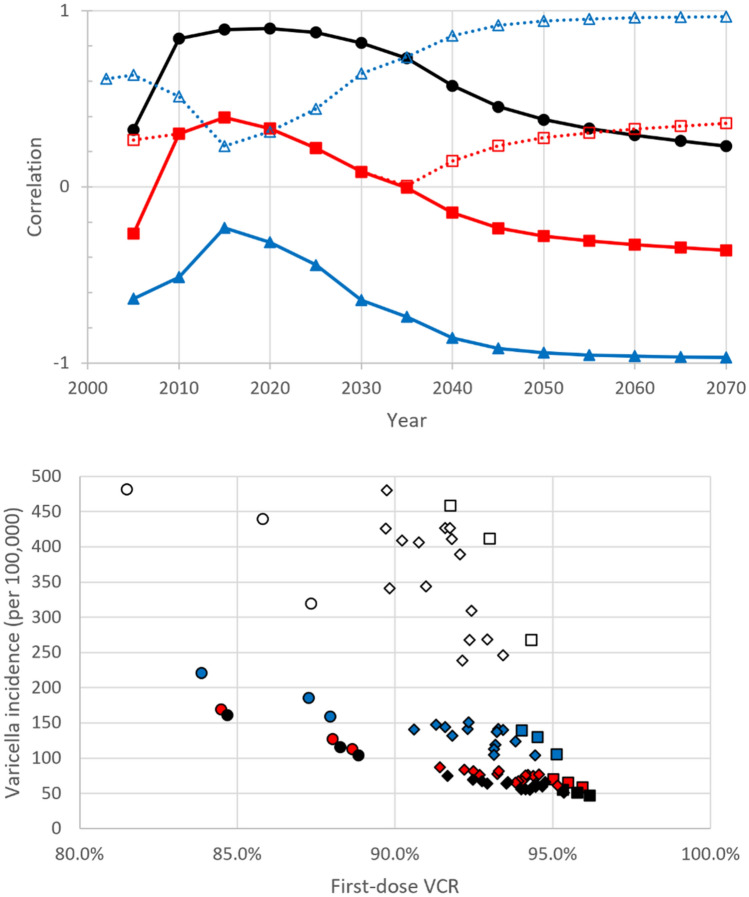
Influence of year of adoption, first-dose VCR, and second-dose VCR on varicella incidence under strategy A. Top row: (Solid lines, filled markers) Correlation. (Dotted lines, open markers) Magnitude of correlation. Correlation between varicella incidence under strategy A and (circles) year of adoption of mandatory UVV, (triangles) equilibrium first-dose VCR parameter (vcr_equilibrium_1), and (squares) equilibrium second-dose VCR parameter (vcr_equilibrium_2). Bottom row: Varicella incidence versus time-varying first-dose VCR in (open markers) 2020, (blue-filled markers) 2040, (red-filled markers) 2060, (black) 2070 for strategy A. Three regions with lowest and highest first-dose equilibrium VCR (vcr_equilibrium_1) are shown as circles and squares, respectively. Other regions are shown as diamonds.

Nationally, the overall decline in varicella incidence from 2020 to 2070 was 82.8% under strategy A (regionally: [69.1%, 89.2%]), see Fig. 1. Although early introduction of UVV accelerated the decline in varicella incidence, the influence of the year of UVV introduction on varicella incidence was transient. Figure 2 (top) plots the correlation between varicella incidence and (a) the year of adoption of mandatory UVV, (b) the long-term first-dose VCR (*vcr_equilibrium_1*; equivalently, equilibrium first-dose VCR), and (c) the long-term second-dose VCR (*vcr_equilibrium_2*; equivalently, equilibrium second-dose VCR). Correlation between varicella incidence and the year of adoption of mandatory UVV peaked in 2020 and decreased over the 2020–2070 time-horizon from 0.90 in 2020 to 0.23 in 2070. In contrast, the magnitude of the correlation between varicella incidence and equilibrium first-dose VCR increased from 0.31 in 2020 to 0.97 in 2070. In other words, year of adoption of mandatory UVV is correlated with short-term varicella incidence and outcomes, whereas long-term equilibrium first-dose VCR is correlated with long-term varicella incidence and outcomes. An alternative illustration of this relationship is presented in Fig. 2 (bottom), which plots varicella incidence versus time-varying fist-dose varicella VCR for various years in the 2020–2070 time-horizon. From 2020 onwards, the relationship between varicella incidence and first-dose VCR becomes increasingly linear, i.e., the magnitude of the correlation between these two quantities is increasing over the time-horizon. In 2020 there is overlap in varicella incidence between the three regions with highest equilibrium first-dose VCR (Piemonte [96.5%], Umbria [96.9%], Basilicata [97.2%]) and the three regions with lowest equilibrium first-dose VCR (Prov. Auton. Bolzano [86.5%], Campania [90.0%], Calabria [90.3%]). However, from 2040 onwards, the three regions with highest equilibrium first-dose VCR (circles) have consistently lower varicella incidence than the three regions with lowest equilibrium first-dose VCR (squares). See Supplementary Sect. S2.2 for additional results.

**Figure 3:**
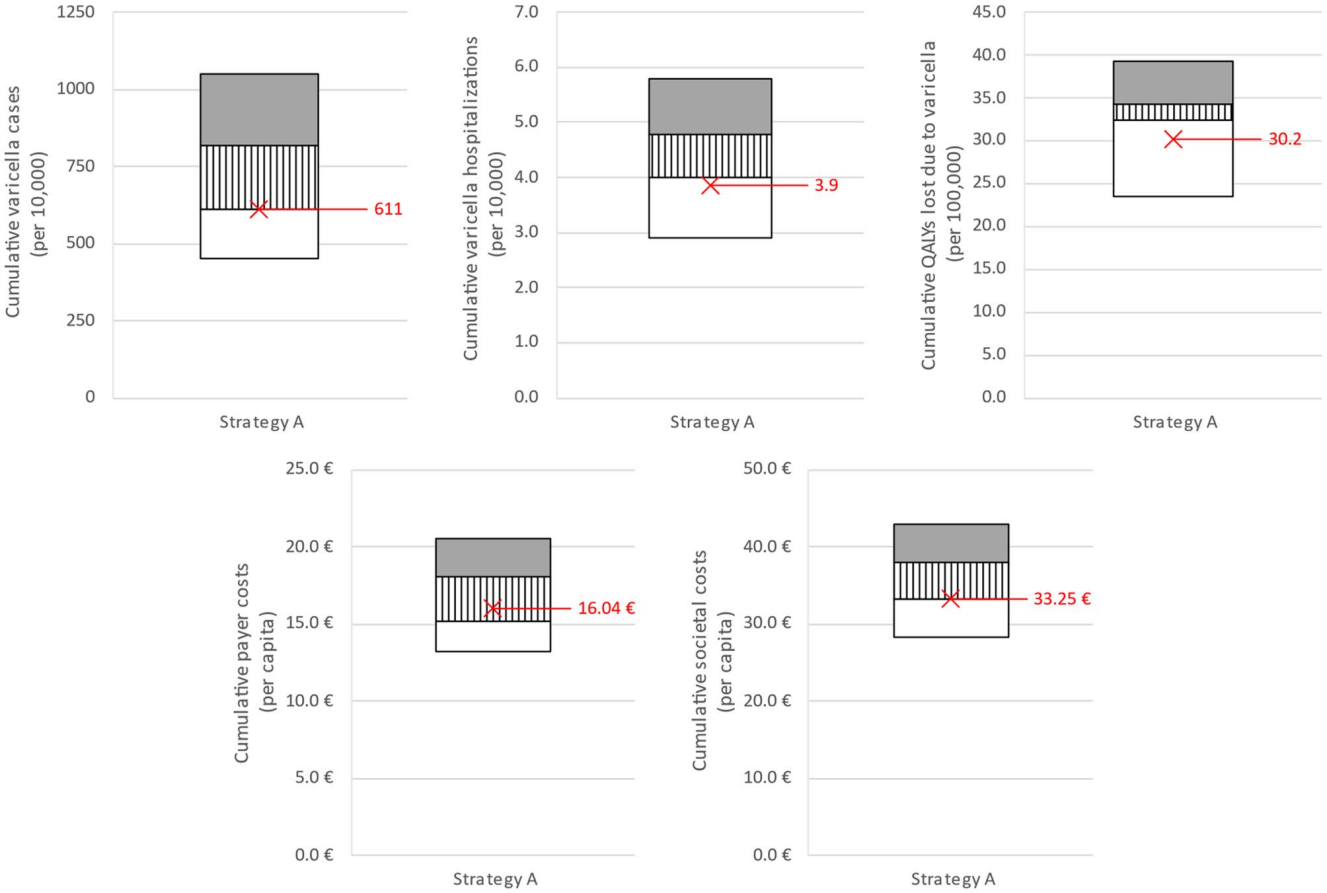
Cumulative outcomes under strategy A (2020–2070). Partitioned rectangles indicate (upper horizontal boundary) maximum of 21 regional values, (boundary between the shaded and striped areas) average of three regions with lowest equilibrium first-dose VCR (vcr_equilibrium_1), (boundary between striped and white areas) average of three regions with highest equilibrium first-dose VCR (vcr_equilibrium_1), and (lower horizontal boundary) minimum of 21 regional values. (Red exes) National average.

Correlation between cumulative outcomes and long-term equilibrium first-dose VCR (*vcr_equilibrium_1*) was also observed. The three regions with lowest *vcr_equilibrium_1* (Prov. Auton. Bolzano [86.5%], Campania [90.0%], Calabria [90.3%]) consistently underperformed compared to the three regions with highest *vcr_equilibrium_1* (Piemonte [96.5%], Umbria [96.9%], Basilicata [97.2%]), see Fig. 3. Specifically, under strategy A the three regions with lowest *vcr_equilibrium_1* recorded 34.3% more varicella cases, 20.0% more varicella hospitalizations, 5.9% more QALYs lost, 22.2% more costs under the payer perspective, and 14.6% more costs under the societal perspective over the 2020–2070 time-horizon.

### Varying first- and second-dose VCRs

**Figure 4:**
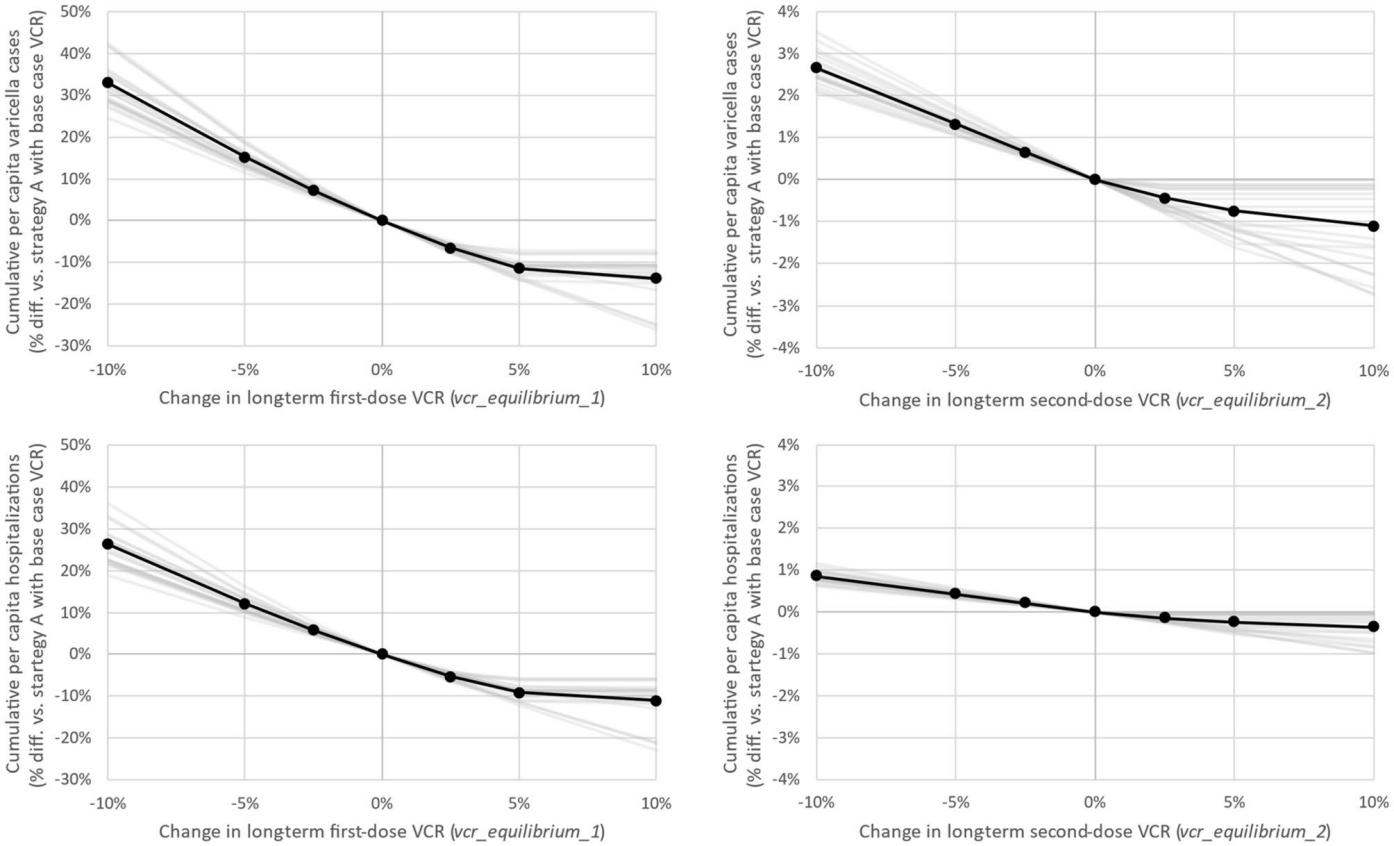
Cumulative outcomes versus change in equilibrium first- and second-dose VCR. (Black line, circles) National outcomes for strategy A. (Gray lines) Regional outcomes for strategy A.

Figure 4 illustrates the change in cumulative varicella cases and hospitalizations over the 50-year time-horizon (2020–2070) in each region under strategy A as equilibrium first-dose VCR (*vcr_equilibrium_1*; Fig. 4, left panels) and equilibrium second-dose VCR (*vcr_equilibrium_2*; Fig. 4, right panels) are varied. To allow for comparison between regions, change in an outcome’s value was measured as a percentage of the outcome’s value under strategy A (with base case values of *vcr_equilibrium_1* and *vcr_equilibrium_2*). Equilibrium first- and second-dose VCR were increased or decreased by up to 10% (up to a maximum VCR of 100%). Outcomes were more sensitive to decreases in first-dose VCR than they were to increases. Additionally, outcomes were most sensitive to first-dose VCR versus second-dose VCR. Nationally, a drop in first-dose VCR of 10% resulted in + 33.0% and + 26.3% additional cumulative varicella cases and hospitalizations, respectively, while a drop in second-dose VCR of 10% resulted in + 2.7% and + 0.8% more cumulative varicella cases and hospitalizations.

**Figure 5:**
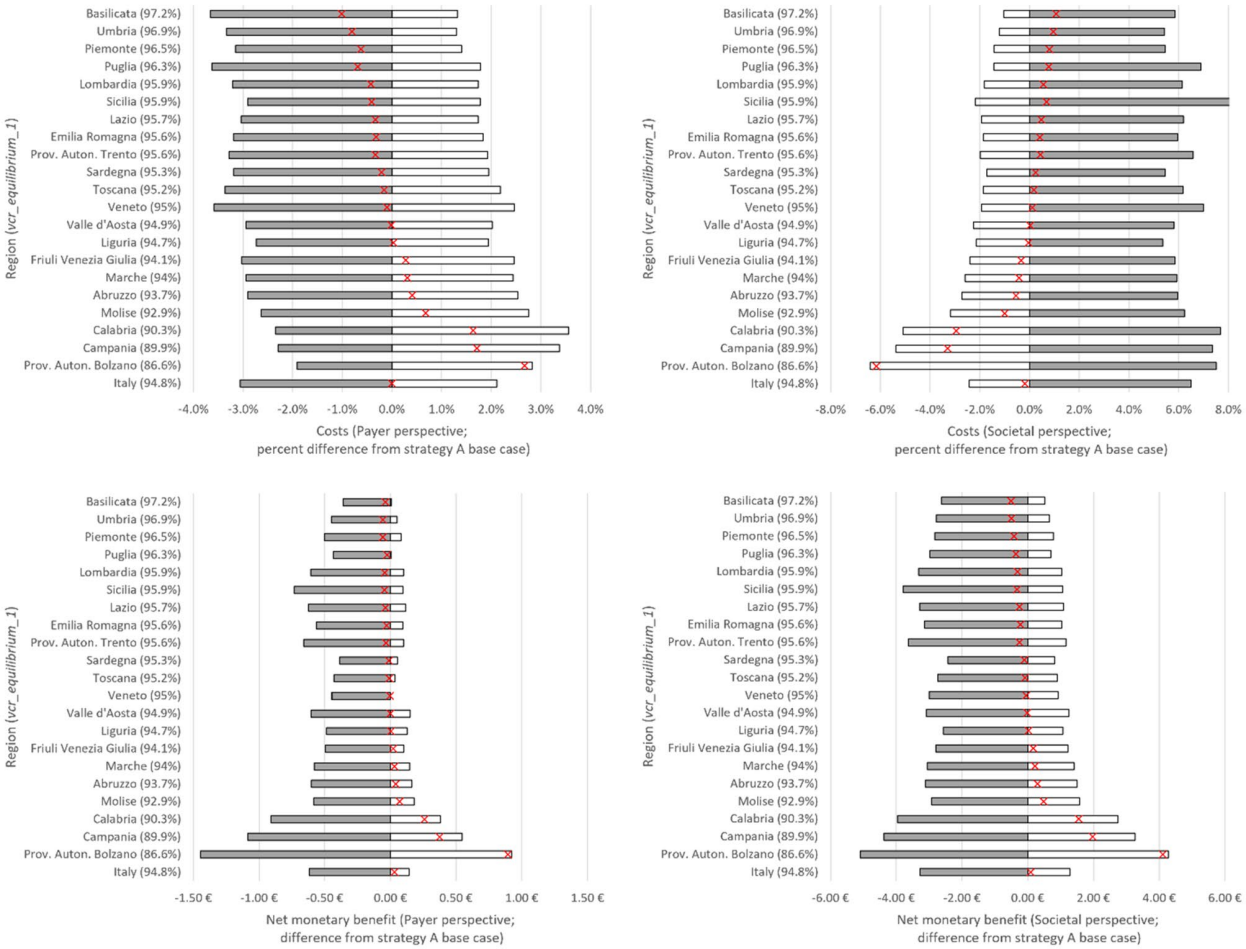
Cumulative cost and NMB versus regional equilibrium first-dose VCR under strategy A. Top row: Payer (left) and societal (right) costs. (Gray bars) Percent change in cost when vcr_equilibrium_1 is decreased by 10%. (White bars) Percent change in cost when vcr_equilibrium_1 is increased by 10%. (Red exes) Percent change in cost when vcr_equilibrium_1 is equal to the national average. Bottom row: Per capita payer (left) and societal (right) NMB with respect to strategy A with base case vcr_equilibrium_1. (Gray bars) NMB when vcr_equilibrium_1 is decreased by 10%. (White bars) NMB when vcr_equilibrium_1 is increased by 10%. (Red exes) NMB when vcr_equilibrium_1 is uniformly equal to the national average.

Figure 5 illustrates changes in costs and NMB by region under strategy A when long-term equilibrium first-dose VCR (*vcr_equilibrium_1*) is increased or decreased by 10%. Regions with lower *vcr_equilibrium_1* tended to be more sensitive than regions with larger *vcr_equilibrium_1*. As in Fig. 4, change in an outcome’s value was measured as a percentage of the outcome’s value under strategy A (with base case *vcr_equilibrium_1*). While costs increased with increasing *vcr_equilibrium_1* under the payer perspective*,* when productivity costs were included (i.e., under the societal perspective), we see that increasing VCR led to a decline in societal costs for all regions. In contrast, NMB increased with increasing *vcr_equilibrium_1* in all regions under both perspectives. When all regions’ equilibrium first-dose VCR was set uniformly equal to the national average we recorded a surplus in NMB under both perspectives. The surplus to regions with lower-than-average equilibrium first-dose VCR was roughly 7.2 and 4.8 times larger than the deficit to regions with higher-than-average equilibrium first-dose VCR under the payer and societal perspectives, respectively.

## Discussion

A national-level dynamic transmission model for varicella vaccination was adapted to a multi-regional model using regional-level demographic data and VCR trends (2013–2019). To the authors’ knowledge, this is the first model to project regional differences in varicella outcomes across a country due to regional differences in varicella VCR. Significant differences in outcomes were observed between high-VCR regions versus low-VCR-regions, with the model showing greater sensitivity to first-dose VCR. For example, the three regions with lowest first-dose VCRs reported 34.3%, 20.0%, and 5.9% more varicella cases, hospitalizations, and QALYs lost over 50 years (2020–2070) than the three regions with highest first-dose VCR under the strategy A (i.e., first- and second-dose MMRV-MSD). Qualitatively similar differences were observed with respect to second-dose VCR, however, with lower magnitude. Scenario analysis indicated that outcomes for low-VCR regions were more sensitive to changes in VCR than outcomes for high-VCR regions. Our results imply that the most cost-effective strategy for improving varicella outcomes is to increase first-dose VCR in low-VCR regions. This is line with WHO recommendation to maintain > 80% VCR for UVV in countries to avoid possible age shift. One-dose UVV is sufficient to reduce mortality and severe morbidity, whereas two-dose UVV is needed to reduce cases and outbreaks^1^.

The COVID-19 pandemic resulted in significant disruption to pediatric vaccination programs and negatively impacted VCRs^20,24,25^. Although we do not explicitly model the effects of the COVID-19 pandemic (e.g., with respect to changes in vaccine coverage, social mixing patterns, etc.), our results highlight the importance of prioritizing recovery of VCRs to pre-pandemic levels by illustrating the significant impact that VCRs can have on varicella outcomes. Moreover, the framework developed for the multi-regional model admits analysis of different vaccination catchup scenarios that can be tailored to the regional level. Thus, the multi-regional model is a potentially valuable tool in evaluating different recovery strategies.

Finally, this model was consistent with other studies’ estimates of overall decline in varicella incidence following the introduction of a UVV program^14,26–31^. Under both perspectives, and for all regions, strategy A (first- and second-dose MMRV-MSD) dominated the remaining strategies (B–D) over the 50-year time-horizon (2020–2070). Robustness of our modelling results was confirmed through PSA and DSA.

This modelling work was subject to several limitations. Where possible our study used pre-2020 input data did not assess the impact of the COVID-19 pandemic of the subsequent societal and governmental response. Separate modelling work would be needed to fully investigate the potential short- and long-term impact of COVID-19 on varicella VCRs and transmission dynamics. Second, we assumed the same first- and second-dose vaccine uptake regardless of vaccine formulation, despite the potential for higher vaccine uptake when varicella vaccines are co-administered with MMR^32^. Because the exact magnitude of the improvement in uptake when comparing monovalent versus quadrivalent vaccine formulations is unknown, our analysis was limited to exploring the effect of different vaccine uptake rates as part of the scenario analysis. Third, to limit computational complexity of this model we implemented the switch from GSK to MSD vaccines through time-varying vaccine performance parameters instead of more complex vaccine-switching models^31^. Similarly, even though a dynamic population model might be appropriate for the ageing Italian population, a static demographic model was employed to limit computational complexity. These simplifications were necessary to ensure that the regional model would be computationally tractable. Additionally, list prices for varicella-containing vaccines were used in the analysis since tender prices are not publicly available. Our model was sensitive to price changes and price change may impact the magnitude of the costs and comparisons between strategies, though not the impact of VCR on clinical burden. Finally, we have used the temporary immunity model to estimate exogenous boosting and its duration. There is ongoing research on alternative models of the exogenous boosting mechanism such as progressive immunity^33^. We do not include HZ outcomes in our cost effectiveness analysis; however, different modelling approaches may lead to different outcomes since our model assumes that HZ cases contribute to the force of infection.

In conclusion, our model predicted significant disparities in varicella outcomes across Italian regions due to differences in varicella VCRs. Regions with lowest VCRs had significantly higher varicella cases, hospitalizations, and costs compared to the regions with highest VCRs. VCR recovery efforts should prioritize regions with low VCRs, and in particular, low first-dose VCRs, in order to reduce varicella-related morbidity and mortality. Our model also showed that all two-dose strategies significantly reduce the burden of varicella at national and regional levels; however, strategy A (first- and second-dose MMRV-MSD) is the dominant strategy over the 50-year time-horizon (2020–2070) under both payer and societal perspectives.

## Methods

We previously developed, parameterized, and published a dynamic transmission model for varicella zoster virus^14,34,35^, which we updated to quantify the potential public health impact of the UVV program in Italy at the national and regional levels. A full description of the methods can be found in Supplementary Sect. S1.

National-level inputs that were derived from literature comprised of epidemiological inputs, vaccine performance inputs, healthcare resource use inputs, and productivity loss inputs (Table 1), and QALY^36^ parameters for healthy and infected individuals. With respect to productivity loss, we adopted the conservative assumption of no productivity loss after retirement (≥ 65 years). National-level inputs that were calibrated comprised parameters for scaling the force of infection by age (i.e., relative-risk parameters), the HZ reactivation rate, the duration of natural maternal immunity, and the probability of hospitalization per varicella infection by age. Relative-risk and natural maternal immunity parameters were calibrated to national pre-UVV varicella seroprevalence data^37,38^. Herpes zoster reactivation parameters were calibrated to national HZ incidence data^39,40^. We note that HZ dynamics were included in the model since the modelling framework we applied assumes that HZ reactivations contribute to the force of infection. Parameters for the probability of hospitalization per varicella infection by age were calibrated to hospitalization incidence^12^. In contrast to national-level parameters, demographic and VCR parameters were assumed to vary by region. Demographic parameters were calibrated to regional-level age-stratified population, fertility, and mortality data^41^. We assumed that varicella VCRs converge to MMR VCRs in the long-term, i.e., we assumed that in equilibrium varicella and MMR VCRs were equal. Thus, the equilibrium first-dose varicella VCR parameter (*vcr_equilibrium_1*) (i.e., the asymptotic, equivalently long-term, value of the first-dose varicella VCR) and the equilibrium second-dose varicella VCR parameter (*vcr_equilibrium_2*) (i.e., the asymptotic value of the second-dose varicella VCR) were calibrated to regional average MMR VCRs (2013–2019)^17^. The remaining VCR parameters were calibrated to regional varicella VCRs (2013–2019)^17^. For a detailed description of data and calibration methods related to seroprevalence, HZ incidence, and regional varicella VCR parameters, refer to Supplementary Sections S1.3–S1.4 and S2.1.

**Table 1:** Healthcare resource use and cost parameters.

Parameter	Ages						Source
	< 15	15–17	18–44	45–64	65–74	75 +	
Number of outpatient visits per varicella case (n_GP_)	1						Assumption, expert opinion
Cost per outpatient visit (c_GP_)	29.00 €	23.31 €					^43,44^
Fraction of varicella cases requiring OTC drugs (p_OTC_)	90%						Assumption, expert opinion
Cost of outpatient drugs (c_OTC_)	11.16 €	39.20 €					^19^
Fraction of varicella cases requiring additional diagnostics, e.g., lab work (p_LAB_)	1%						Assumption, expert opinion
Cost of additional labwork (c_LAB_)	40.96 €						^44^
Productivity loss per varicella case (days; out_PL_)	0.7	5.7			0.0		^45–48^
Cost of productivity loss (per day; c_PL_)	123.52 €						^41^
Total outpatient direct costs per varicella case^A,B^	39.45 €	59.00 €			0.00 €		Calculation
Total outpatient indirect costs per varicella case^A,C^	86.46 €	704.06 €			0.00 €		
Duration of hospitalization (days; in_PL_)	7.5		5	7	8	10	^12,21^
Hospitalization cost (per day, c_H_)	862.88 €						^14,49^
Total direct costs per hospitalization^A,B^	6,471.60 €		4,314.40 €	6,040.16 €	6,903.04 €	8,628.80 €	Calculation
Total indirect costs per hospitalization^A,C^	926.40 €		617.60 €	864.64 €	0.00 €		

	Marginal cost of varicella vaccine/component^A^		Marginal cost of vaccine administration^A^		Marginal cost of adverse events^A,D^		Source
	First dose	Second dose	First dose	Second dose	First dose	Second dose	
Strategy A: 2 doses of MMRV—MSD	38.25 €	38.25 €	0 €		0.43 €	0 €	^44,50–52^
Strategy B: 2 doses of MMRV—GSK	40.25 €	40.25 €	0 €		0.43 €	0 €	
Strategy C: 1st dose V/2nd dose MMRV—MSD	38.25 €	38.25 €	6.95 €	0 €	0 €		
Strategy D: 1st dose V/2nd dose MMRV—GSK	39.32 €	40.25 €	6.95 €	0 €	0 €		

Strategy A: MMRV-MSD/MMRV-MSD, Strategy B: MMRV-GSK/MMRV-GSK, Strategy C: V-MSD/MMRV-MSD, and Strategy D: V-GSK/MMRV-GSK.

^A^Costs are inflated to 2022 values^41^, see section S1.5.4 for additional details.

^B^Total outpatient and inpatient direct costs per varicella case is equal to (nGP × cGP + pOTC × cOTC + pLAB × cLAB) and (nH × cH), respectively.

^C^Total outpatient and inpatient indirect costs per varicella case is equal to (outPL × cPL) and (nH × cPL), respectively.

^D^Administration of MMRV as the first dose in the series increases the risk febrile seizure by 1/4,587. The hospitalization cost for febrile seizure is 1950.56 €. Adverse event costs for first-dose MMRV are 1837.61 €/4587 = 0.43 €.

The incremental cost-effectiveness ratio of a given strategy, which is measured against a reference strategy by computing the ratio of the difference in costs to the difference in QALYs, is a commonly used metric for establishing cost-effectiveness when the reference strategy is “no vaccination”. However, this analysis pertains to a country where UVV is already established, i.e., UVV strategies are only compared to each other and not to a “no vaccination” scenario. Consequently, we expected the difference in QALYs between UVV strategies to be small, and therefore, we choose to use an alternative (and equivalent) metric, the net monetary benefit (NMB), which is defined as

1 $$NMB=\left(difference\, in\, QALYs\right)\times WTP-\left(difference\, in\, costs\right)$$

where WTP is the willingness-to-pay threshold. In this analysis we assumed a WTP of 1 × GDP per capita or 26,700€ per QALY^42^.

### Supplementary Information

Supplementary Information 1.

Supplementary Information 2.

## Data Availability

All data generated or analyzed during this study are included in this published article (and its Supplementary Information files).
