# Supplementary material for: The clinical and economic costs associated with regional disparities in varicella vaccine coverage in Italy over 50 years (2020–2070)
Source: Sci Rep. 2024 May 24;14:11929. doi: 10.1038/s41598-024-60649-8 (PMC11126631; doi:10.1038/s41598-024-60649-8)
Supplement: Supplementary file 1 — Supplementary Information 1. [file 41598_2024_60649_MOESM1_ESM.docx]

**The Clinical and Economic Costs Associated with Regional Disparities in Varicella Vaccine Coverage in Italy Over 50 Years (2020-2070)**

**Supplemental Methods (S1)**

**Authors:** Lang, J.C., PhD^1*^, Samant, S., MPH^2^, Cook, J.R., PhD^3^, Ranjan, S., MSc ^3^, Senese, F., MPH^4^, Starnino, S., PhD^5^, Giuffrida, S., MD^6^, Azzari, C., MD^7^, Baldo, V., MD^8^, Pawaskar, M., PhD^2^

^1*^ Biostatistics and Research Decision Sciences, Merck Canada Inc., Kirkland, QC, Canada; [john.lang@merck.com](mailto:john.lang@merck.com)

^2^ Center for Observational and Real-World Evidence, Merck & Co., Inc., Rahway, NJ, USA

^3^ CHEORS, North Wales, PA, USA

^4^ Market Access, MSD Italy, Rome, Italy

^5^ Medical Affairs, MSD Italy, Rome, Italy

^6^ LHU Reggio Calabria, Calabria, Italy

^7^ Department of Health Sciences, University of Florence, and Meyer Children’s University Hospital, Florence, Italy

^8^ Department of Cardiac Thoracic Vascular Sciences, Hygiene and Public Health Unit, and Public Health University of Padua, Padua, Italy

**Contents**

[**S1.1 Model Diagram and Definitions** 4](#_Toc161175589)

[**S1.2 Model Demographic Parameters** 7](#_Toc161175590)

[**S1.3 Ordinary Differential Equations** 10](#_Toc161175591)

[**S1.4 Calibration Steps for Vaccine Coverage Parameters** 14](#_Toc161175592)

[***S1.4.1 Epidemiological Parameters*** 14](#_Toc161175593)

[***S1.4.2 Vaccine Coverage Parameters*** 18](#_Toc161175594)

[**S1.5 Model Inputs (QALYs and Cost)** 22](#_Toc161175595)

[***S1.5.1 Epidemiological inputs*** 22](#_Toc161175596)

[***S1.5.2 Vaccine Efficacy*** 23](#_Toc161175597)

[***S1.5.3 QALY inputs*** 24](#_Toc161175598)

[***S1.5.4 Cost inputs*** 25](#_Toc161175599)

[**S1.6 Distributions in PSA Analysis for Cost and QALY Parameters** 28](#_Toc161175600)

[***S1.6.1 Calibrated vaccine coverage parameters*** 30](#_Toc161175601)

[***S1.6.2 Herpes zoster related parameters*** 30](#_Toc161175602)

[***S1.6.3 Cost and QALY parameters*** 31](#_Toc161175603)

[***S1.6.4 Vaccine parameters*** 32](#_Toc161175604)

[***S1.6.5 Vaccine failure rate*** 32](#_Toc161175605)

[***S1.6.6 Vaccine take-rates*** 33](#_Toc161175606)

[***S1.6.7 Waning of temporary immunity*** 34](#_Toc161175607)

[***S1.6.8 Waning of durable immunity*** 34](#_Toc161175608)

[***S1.6.9 Relative infectiousness of breakthrough varicella*** 34](#_Toc161175609)

[**References** 35](#_Toc161175610)

**Figures**

[**Figure S1.1: Disease and vaccination structure for dynamic transmission model*** 6](#_Toc161175611)

[**Figure S1.2 National-level model calibration and validation results** 17](#_Toc161175612)

**Tables**

[**Table S1.1: Definition of model compartments** 5](#_Toc161175613)

[**Table S1.2: Summary of demographic model inputs for MMRV DTM** 9](#_Toc161175614)

[**Table S1.3: Age strata for MMRV DTM** 9](#_Toc161175615)

[**Table S1.4: Calibrated parameters for VZV DTM (excluding demographic parameters)** 15](#_Toc161175616)

[**Table S1.5: Description of parameters used in sigmoidal function** $\boldsymbol{sigFun}$ 19](#_Toc161175617)

[**Table S1.6: Average MMR VCR and fitted equilibrium varicella VCR parameters** 20](#_Toc161175618)

[**Table S1.7: Epidemiological parameters** 22](#_Toc161175619)

[**Table S1.8: Vaccine properties** 23](#_Toc161175620)

[**Table S1.9: QALYs for healthy individuals** 25](#_Toc161175621)

[**Table S1.10: QALY loss for varicella infection** 25](#_Toc161175622)

[**Table S1.11: Vaccination costs** 26](#_Toc161175623)

[**Table S1.12: Vaccination costs by strategy for years 2020 onward** 26](#_Toc161175624)

[**Table S1.13: Drug costs by age (2022 currency)** 27](#_Toc161175625)

[**Table S1.14: Summary of outpatient cost data (2022 currency)** 27](#_Toc161175626)

[**Table S1.15: Parameter and values used in the sensitivity analyses** 28](#_Toc161175627)

# **S1.1** **Model Diagram and Definitions**

Model compartments are summarized in Table S1.1 and model disease-state transitions are illustrated in Figure S1.1. The set of health states and corresponding transitions were similar to previous cost-effectiveness analyses of UVV, e.g., [1, 2, 3, 4, 5]. Model states and transitions were implemented separately for each region and national results were estimated by aggregating regional results.

**Table S1.1: Definition of model compartments**

| **Compartment*** | **Description** |
| --- | --- |
| **Unvaccinated compartments** | |
| $m_{j}$ | passively immune |
| $s_{j}$ | susceptible to varicella infection |
| $e_{j}$ | latent varicella |
| $i_{j}$ | infectious varicella |
| $r_{j}$ | recovered from varicella with high HZ immunity |
| $w_{j}$ | low HZ immunity due to waning effects |
| **Varicella vaccination compartments** | |
| $v_{j,l}$ | long-lasting immunity following $l-$dose vaccination |
| ${vq}_{j,l}$ | temporary immunity following $l-$dose vaccination |
| ${vs}_{j,l}$ | susceptible to varicella following $l-$dose vaccine waning |
| $s_{j,l}$ | susceptible to varicella following $l-$dose vaccine failure |
| $e_{j,l}$ | latent varicella following $l-$dose vaccine failure |
| ${rvv}_{j,l}$ | high HZ immunity following $l-$dose vaccination |
| ${wvv}_{j,l}$ | low HZ immunity following $l-$dose vaccine waning |
| **Breakthrough varicella infection compartments** | |
| ${evb}_{j,l}$ | latent varicella following $l-$dose vaccine waning |
| ${ivb}_{j}$ | infectious varicella |
| ${rvb}_{j}$ | recovered from varicella with high HZ immunity |
| ${wvb}_{j}$ | low HZ immunity due to waning |
| **HZ reactivation compartments** | |
| $z_{j}$ | infectious with wild-type HZ |
| ${zvacc}_{j}$ | infectious with vaccine-type HZ post varicella vaccination |
| ${zwild}_{j}$ | infectious with wild-type HZ post breakthrough varicella |
| ${rz}_{j}$ | recovered from HZ with high HZ immunity |
| **Death compartments** | |
| ${ndv}_{j}$ | death from varicella |
| ${ndz}_{j}$ | death from HZ |

* Subscript j indicates compartment for age strata j.

**Figure S1.1: Disease and vaccination structure for dynamic transmission model***


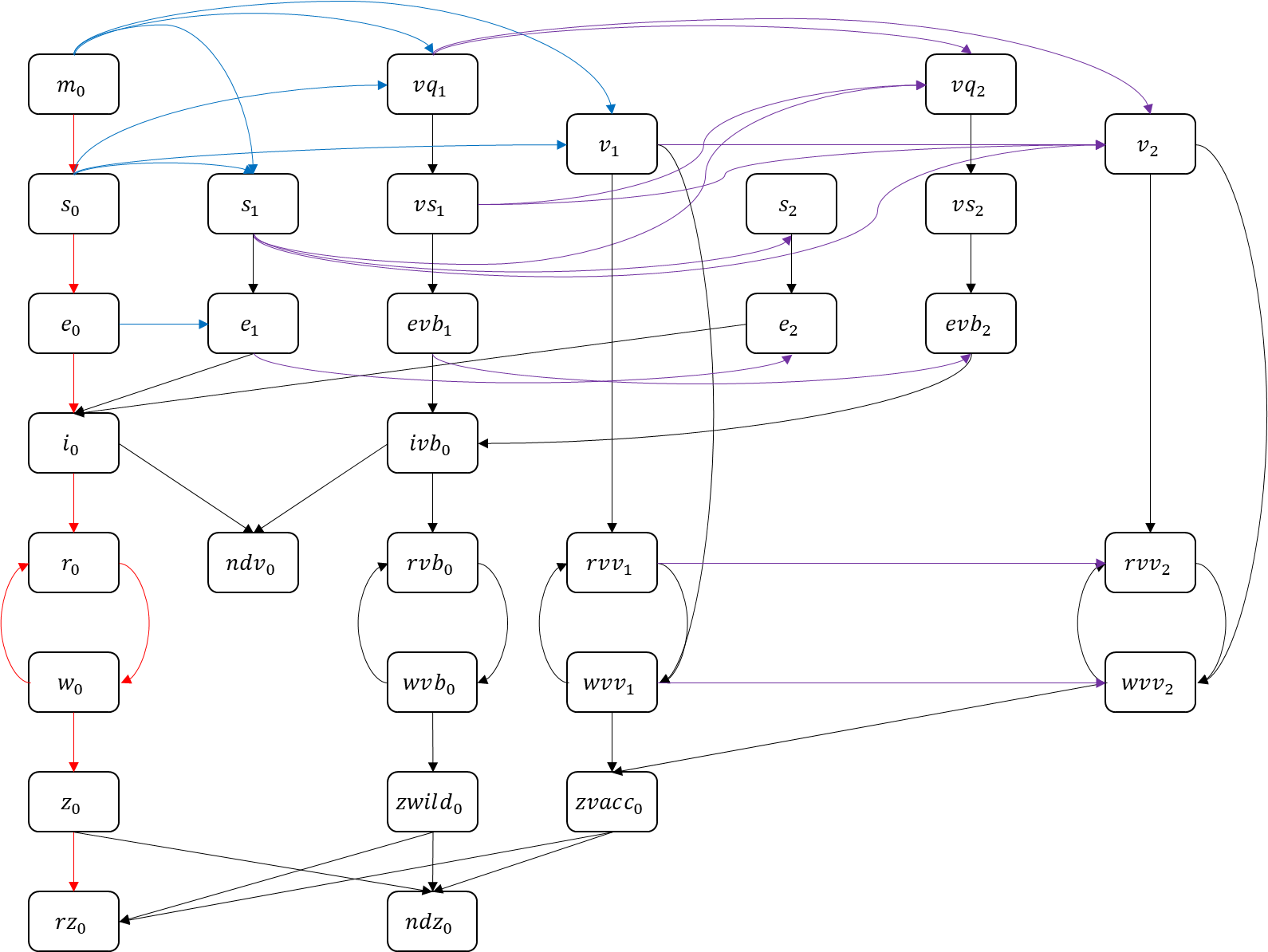


*Blue lines represent vaccination with UVV first dose, purple lines represent vaccination with second dose, red lines represent varicella natural history and black lines represent all other transitions in the figure.

# **S1.2** **Model Demographic Parameters**

We apply the modeling framework of Hethcote [6] to model demographic structure. Conceptually, our goal is to choose growth rate ($q$) and background mortality rates ($\mu_{j}$) to simultaneously minimize the difference between (a) the model age probability density function (PDF) and the empirical age PDF and (b) the model background mortality rates ($\mu_{j}$) and the empirical background mortality rates ($\hat{\mu}_{j}$). Specifically, we begin with the parameterization of background death rates

$$\mu_{j}=\exp\left\{ \begin{matrix} m_{\left\{ 0,1 \right\}} \\ m_{\left\{ 1,2 \right\}} \\ \begin{matrix} m_{\left\{ 2,20 \right\}}^{a}\times\left( mid_{j}-m_{\left\{ 2,20 \right\}}^{b} \right)^{2}+m_{\left\{ 2,20 \right\}}^{c} \\ m_{\left\{ 20,30 \right\}} \\ m_{\left\{ 30,\infty\right\}}^{a}\times\left( mid_{j}-m_{\left\{ 30,\infty\right\}}^{b} \right) \end{matrix} \end{matrix} \right.\begin{matrix} if mid_{j}\in\left[ 0,1 \right) \\ if mid_{j}\in[1,2) \\ \begin{matrix} if mid_{j}\in[2,20) \\ if mid_{j}\in\left[ 20,30 \right) \\ otherwise \end{matrix} \end{matrix} ,$$

where $mid_{j}$ is the centroid of age strata *j* with respect to empirical death rates $\hat{\mu}_{j}$. We then minimize the joint objective function

$$\left( 1-0.999 \right)\times\sum_{j} \left( \log\left( \mu_{j} \right)-\log\left( \hat{\mu}_{j} \right) \right)^{2}+0.999\sum_{j} \left( \frac{{N_{j}-\hat{N}}_{j}}{w_{j}} \right)^{2}$$

with respect to parameters

$$\left\{ q, m_{\left\{ 0,1 \right\}}, m_{\left\{ 1,2 \right\}}, m_{\left\{ 2,20 \right\}}^{a}, m_{\left\{ 2,20 \right\}}^{b}, m_{\left\{ 2,20 \right\}}^{c},m_{\left\{ 20,30 \right\}},m_{\left\{ 30,\infty\right\}}^{a},m_{\left\{ 30,\infty\right\}}^{b} \right\} ,$$

where $w_{j}$ is the width of age strata $j$,

$$m_{j}=\lim_{x\to\mu_{j}+q} \frac{x}{\exp\left[ w_{j}\times x \right]-1}$$

is the maturation rate for age strata $j$,

$$N_{j}=\left\{ \begin{matrix} 1-\sum_{j>1} N_{j} & j=1 \\ \frac{d_{j-1}}{d_{j}+\mu_{j}+q}N_{j-1} & j>1 \end{matrix} \right.$$

is the fraction of individuals in age strata $j$ in the model, and $\hat{N}_{j}$ is the fraction of individuals in age strata $j$ in the empirical data.

Fertility rates ($f_{j}$) are parameterized with a gaussian function, i.e.,

$$f_{j}=\left\{ \begin{matrix} F_{const}\exp\left( - \left( \frac{\left( mid_{j}-F_{mean} \right)^{2}}{2 F_{std}} \right) \right) & mid_{j}\in\left[ 10,50 \right) \\ 0 & otherwise \end{matrix} \right. .$$

Fertility rates are then chosen by minimizing

$$\sum_{j} \left( f_{j}-\hat{f}_{j} \right)^{2}$$

with respect to parameters $\left\{ F_{const},F_{mean},F_{std} \right\}$subject to the constraint given by the Lotka equation, i.e.,

$$d_{1}+\mu_{1}+q=\underset{=B^{s}+B^{m}}{\underbrace{\sum_{j} f_{j}\times N_{j}}} ,$$

where $\hat{f}_{j}$ are the empirical fertility rates.

**Table S1.2: Summary of demographic model inputs for MMRV DTM**

| **Input parameter** | **Description** | **Source** |
| --- | --- | --- |
| Age strata | Age strata for MMRV DTM | Assumption |
| Population by age | Population by age for Italy and regions (2021) | [7] |
| Births per person per year | Births per person per year by age for Italy and regions (2020) | [7, 8] |
| Annual probability of death by age | Annual probability of death by age for Italy and regions (2020) | [9] |
| Population growth projections | Population growth projections for 2020-2070 for Italy and regions (for demographic model validation only) | [10] |

**Table S1.3: Age strata for MMRV DTM**

| **Age**  **group**  **(j)** | **Minimum**  **age**  **(years)** | **Width**  **(years)** | **Notes** | **Age**  **group**  **(j)** | **Minimum**  **age**  **(years)** | **Width**  **(years)** | **Notes** |
| --- | --- | --- | --- | --- | --- | --- | --- |
| 1 | 0 | 0.08 | <1 month | 26 | 11 | 1 | 11-12 years |
| 2 | 0.08 | 0.42 | 1-6 months | 27 | 12 | 1 | 12-13 years |
| 3 | 0.50 | 0.42 | 6-11 months | 28 | 13 | 1 | 13-14 years |
| 4 | 0.92 | 0.08 | 11-12 months | 29 | 14 | 1 | 14-15 years |
| 5 | 1.00 | 0.08 | 12-13 months | 30 | 15 | 1 | 15-16 years |
| 6 | 1.08 | 0.08 | 13-14 months | 31 | 16 | 1 | 16-17 years |
| 7 | 1.17 | 0.08 | 14-15 months | 32 | 17 | 1 | 17-18 years |
| 8 | 1.25 | 0.08 | 15-16 months | 33 | 18 | 1 | 18-19 years |
| 9 | 1.33 | 0.08 | 16-17 months | 34 | 19 | 1 | 19-20 years |
| 10 | 1.42 | 0.08 | 17-18 months | 35 | 20 | 5 | 20-25 years |
| 11 | 1.50 | 0.08 | 18-19 months | 36 | 25 | 5 | 25-30 years |
| 12 | 1.58 | 0.08 | 19-20 months | 37 | 30 | 5 | 30-35 years |
| 13 | 1.67 | 0.08 | 20-21 months | 38 | 35 | 5 | 35-40 years |
| 14 | 1.75 | 0.08 | 21-22 months | 39 | 40 | 5 | 40-45 years |
| 15 | 1.83 | 0.08 | 22-23 months | 40 | 45 | 5 | 45-50 years |
| 16 | 1.92 | 0.08 | 23-24 months | 41 | 50 | 5 | 50-55 years |
| 17 | 2 | 1 | 2-3 years | 42 | 55 | 5 | 55-60 years |
| 18 | 3 | 1 | 3-4 years | 43 | 60 | 5 | 60-65 years |
| 19 | 4 | 1 | 4-5 years | 44 | 65 | 5 | 65-70 years |
| 20 | 5 | 1 | 5-6 years | 45 | 70 | 5 | 70-75 years |
| 21 | 6 | 1 | 6-7 years | 46 | 75 | 5 | 75-80 years |
| 22 | 7 | 1 | 7-8 years | 47 | 80 | 5 | 80-85 years |
| 23 | 8 | 1 | 8-9 years | 48 | 85 | 5 | 85-90 years |
| 24 | 9 | 1 | 9-10 years | 49 | 90 | 10 | 90+ years |
| 25 | 10 | 1 | 10-11 years |  |  |  |  |

# **S1.3 Ordinary Differential Equations**

$$\frac{{dm}_{j}}{dt}= B^{m}\left( t \right)\delta_{1,j}+d_{j-1}\left( {1-\delta}_{1,j} \right)\left( 1-\theta_{j}^{p}\left( t \right)-\theta_{j}^{c}\left( t \right)-\theta_{j}^{s}\left( t \right) \right)m_{j-1}\left( t \right) -\left( d_{j}+\omega^{m}+\mu_{j} \right)m_{j}\left( t \right).$$

$$\frac{{ds}_{j}}{dt}= B^{s}\left( t \right)\delta_{1,j}+ \omega^{m}m_{j}\left( t \right)+d_{j-1}\left( 1-\delta_{1,j} \right)\left( 1-\theta_{j}^{p}\left( t \right)-\theta_{j}^{c}\left( t \right)-\theta_{j}^{s}\left( t \right) \right)s_{j-1}\left( t \right) -\left( d_{j}+\mu_{j}+\lambda_{j} \right)s_{j}\left( t \right).$$

$$\frac{{de}_{j}}{dt}= \lambda_{j}s_{j}\left( t \right)+d_{j-1}\left( {1-\delta}_{1,j} \right)\left( 1-\theta_{j}^{p}\left( t \right)-\theta_{j}^{c}\left( t \right)-\theta_{j}^{s}\left( t \right) \right)e_{j-1}\left( t \right) -\left( d_{j}+\mu_{j}+\epsilon^{n} \right)e_{j}\left( t \right).$$

$$\frac{{di}_{j}}{dt}= d_{j-1}\left( {1-\delta}_{1,j} \right)i_{j-1}\left( t \right)+\epsilon^{n}\left( e_{j}\left( t \right)+ e_{j,1}\left( t \right)+e_{j,2}\left( t \right) \right)- \left( d_{j}+\mu_{j}+\gamma^{n}+d_{j}^{v} \right)i_{j}\left( t \right).$$

$$\frac{{dr}_{j}}{dt}= d_{j-1}\left( {1-\delta}_{1,j} \right)r_{j-1}\left( t \right) +\left( {\xi^{n}}_{j}+{\zeta^{n}}_{j}\lambda_{j} \right)w_{j}\left( t \right)+ \gamma^{n}i_{j}\left( t \right)-\left( d_{j}+\mu_{j}+\delta^{n} \right)r_{j}\left( t \right).$$

$$\frac{{dw}_{j}}{dt}=d_{j-1}\left( {1-\delta}_{1,j} \right)w_{j-1}\left( t \right)+\delta^{n}r_{j}\left( t \right)-\left( d_{j}+\mu_{j}+\sigma_{j}+{\xi^{n}}_{j}+{\zeta^{n}}_{j}\lambda_{j} \right)w_{j}\left( t \right).$$

$$\frac{{dv}_{j,1}}{dt}=d_{j-1}\left( {1-\delta}_{1,j} \right)v_{j-1,1}\left( t \right)+d_{j-1}\left( {1-\delta}_{1,j} \right)\left[ \left( \theta_{j}^{p}\left( t \right)+\theta_{j}^{c}\left( t \right)+\theta_{j}^{s}\left( t \right) \right)P T_{1}\left( m_{j-1}\left( t \right)+s_{j-1}\left( t \right) \right)-\left( \theta_{j}^{b}\left( t \right)+\theta_{j}^{d}\left( t \right) \right)v_{j-1,1}\left( t \right) \right]-\left( d_{j}+\mu_{j}+\pi_{1}+k_{1}\lambda_{j}\left( t \right) \right)v_{j,1}\left( t \right).$$

$$\frac{{dv}_{j,2}}{dt}=d_{j-1}\left( {1-\delta}_{1,j} \right)v_{j-1,2}\left( t \right)+d_{j-1}\left( {1-\delta}_{1,j} \right)\left( \theta_{j}^{b}\left( t \right)+\theta_{j}^{d}\left( t \right) \right)\left[ v_{j-1,1}\left( t \right)+P T_{1}s_{j-1,1}\left( t \right)+T_{2}\left( {vq}_{j-1,1}\left( t \right)+{vs}_{j-1,1}\left( t \right) \right) \right]-\left( d_{j}+\mu_{j}+\pi_{2}+k_{2}\lambda_{j}\left( t \right) \right)v_{j,2}\left( t \right).$$

$$\frac{{dvq}_{j,1}}{dt}=d_{j-1}\left( {1-\delta}_{1,j} \right){vq}_{j-1,1}\left( t \right)+d_{j-1}\left( {1-\delta}_{1,j} \right)\left[ P\left( 1-T_{1} \right)\left( \theta_{j}^{p}\left( t \right)+\theta_{j}^{c}\left( t \right)+\theta_{j}^{s}\left( t \right) \right)\left( m_{j-1}\left( t \right)+s_{j-1}\left( t \right) \right)-\left( \theta_{j}^{b}\left( t \right)+\theta_{j}^{d}\left( t \right) \right){vq}_{j-1,1}\left( t \right) \right]-\left( d_{j}+\mu_{j}+\sigma^{v} \right){vq}_{j,1}\left( t \right).$$

$$\frac{{dvq}_{j,2}}{dt}=d_{j-1}\left( {1-\delta}_{1,j} \right){vq}_{j-1,2}\left( t \right)+d_{j-1}\left( {1-\delta}_{1,j} \right)\left( \theta_{j}^{b}\left( t \right)+\theta_{j}^{d}\left( t \right) \right)\left[ P\left( 1-T_{1} \right)s_{j-1,1}\left( t \right)+\left( 1-T_{2} \right)\left( {vq}_{j-1,1}\left( t \right)+{vs}_{j-1,1}\left( t \right) \right) \right]-\left( d_{j}+\mu_{j}+\sigma^{v} \right){vq}_{j,2}\left( t \right).$$

$$\frac{{dvs}_{j,1}}{dt}= \sigma^{v}{vq}_{j,1}\left( t \right)+d_{j-1}\left( {1-\delta}_{1,j} \right)\left( 1-\theta_{j}^{b}\left( t \right)-\theta_{j}^{d}\left( t \right) \right){vs}_{j-1,1}\left( t \right)-\left( d_{j}+\mu_{j}+\lambda_{j}\left( t \right) \right){vs}_{j,1}\left( t \right).$$

$$\frac{{dvs}_{j,2}}{dt}= \sigma^{v}{vq}_{j,2}\left( t \right)+d_{j-1}\left( {1-\delta}_{1,j} \right){vs}_{j-1,2}\left( t \right)-\left( d_{j}+\mu_{j}+\lambda_{j}\left( t \right) \right){vs}_{j,2}\left( t \right).$$

$$\frac{{ds}_{j,1}}{dt}= d_{j-1}\left( {1-\delta}_{1,j} \right)s_{j-1,1}\left( t \right)+d_{j-1}\left( {1-\delta}_{1,j} \right)\left[ \left( 1-P \right)\left( \theta_{j}^{p}\left( t \right)+\theta_{j}^{c}\left( t \right)+\theta_{j}^{s}\left( t \right) \right)\left( m_{j-1}\left( t \right)+s_{j-1}\left( t \right) \right)-\left( \theta_{j}^{b}\left( t \right)+\theta_{j}^{d}\left( t \right) \right)s_{j-1,1}\left( t \right) \right]-\left( d_{j}+\mu_{j}+\lambda_{j}\left( t \right) \right)s_{j,1}\left( t \right).$$

$$\frac{{ds}_{j,2}}{dt}= d_{j-1}\left( {1-\delta}_{1,j} \right)s_{j-1,2}\left( t \right)+d_{j-1}\left( {1-\delta}_{1,j} \right)\left( 1-P \right)\left( \theta_{j}^{b}\left( t \right)+\theta_{j}^{d}\left( t \right) \right)s_{j-1,1}\left( t \right)-\left( d_{j}+\mu_{j}+\lambda_{j}\left( t \right) \right)s_{j,2}\left( t \right).$$

$$\frac{{de}_{j,1}}{dt}=d_{j-1}\left( {1-\delta}_{1,j} \right)e_{j-1,1}\left( t \right)+d_{j-1}\left( {1-\delta}_{1,j} \right)\left[ \left( \theta_{j}^{p}\left( t \right)+\theta_{j}^{c}\left( t \right)+\theta_{j}^{s}\left( t \right) \right)e_{j-1}\left( t \right)-\left( \theta_{j}^{b}\left( t \right)+\theta_{j}^{d}\left( t \right) \right)e_{j-1,1}\left( t \right) \right]+\lambda_{j}\left( t \right)s_{j,1}\left( t \right)- \left( d_{j}+\mu_{j}+\epsilon^{n} \right)e_{j,1}\left( t \right).$$

$$\frac{{de}_{j,2}}{dt}=d_{j-1}\left( {1-\delta}_{1,j} \right)e_{j-1,2}\left( t \right)+d_{j-1}\left( {1-\delta}_{1,j} \right)\left( \theta_{j}^{b}\left( t \right)+\theta_{j}^{d}\left( t \right) \right)e_{j-1,1}\left( t \right)+\lambda_{j}\left( t \right)s_{j,2}\left( t \right)- \left( d_{j}+\mu_{j}+\epsilon^{n} \right)e_{j,2}\left( t \right).$$

$$\frac{{drvv}_{j,1}}{dt}= d_{j-1}\left( {1-\delta}_{1,j} \right)\left( 1-\theta_{j}^{b}\left( t \right)-\theta_{j}^{d}\left( t \right) \right){rvv}_{j-1,1}\left( t \right)+{\xi^{vv}}_{j}{wvv}_{j,1}\left( t \right)+\lambda_{j}\left( t \right)\left[ k_{1}v_{j,1}\left( t \right)+{\zeta^{vv}}_{j}{wvv}_{j,1}\left( t \right) \right]-\left( d_{j}+\mu_{j}+\delta^{vv} \right){rvv}_{j,1}\left( t \right).$$

$$\frac{{drvv}_{j,2}}{dt}= d_{j-1}\left( {1-\delta}_{1,j} \right)\left[ \left( \theta_{j}^{b}\left( t \right)+\theta_{j}^{d}\left( t \right) \right){rvv}_{j-1,1}\left( t \right)+{rvv}_{j-1,2}\left( t \right) \right]+{\xi^{vv}}_{j}{wvv}_{j,2}\left( t \right)+\lambda_{j}\left( t \right)\left[ k_{2}v_{j,2}\left( t \right)+{\zeta^{vv}}_{j}{wvv}_{j,2}\left( t \right) \right]-\left( d_{j}+\mu_{j}+\delta^{vv} \right){rvv}_{j,2}\left( t \right).$$

$$\frac{{dwvv}_{j,1}}{dt}=d_{j-1}\left( {1-\delta}_{1,j} \right)\left( 1-\theta_{j}^{b}\left( t \right)-\theta_{j}^{d}\left( t \right) \right){wvv}_{j-1,1}\left( t \right)+\delta^{vv}{rvv}_{j,1}\left( t \right)+\pi_{1}v_{j,1}\left( t \right)-\left( d_{j}+\mu_{j}+\chi\sigma_{j}+{\xi^{vv}}_{j}+{\zeta^{vv}}_{j}\lambda_{j}\left( t \right) \right){wvv}_{j,1}\left( t \right).$$

$$\frac{{dwvv}_{j,2}}{dt}=d_{j-1}\left( {1-\delta}_{1,j} \right){wvv}_{j-1,2}\left( t \right)+d_{j-1}\left( {1-\delta}_{1,j} \right)\left( \theta_{j}^{b}\left( t \right)+\theta_{j}^{d}\left( t \right) \right){wvv}_{j-1,1}\left( t \right)+\delta^{vv}{rvv}_{j,2}\left( t \right)+\pi_{2}v_{j,2}\left( t \right)-\left( d_{j}+\mu_{j}+\chi\sigma_{j}+{\xi^{vv}}_{j}+{\zeta^{vv}}_{j}\lambda_{j}\left( t \right) \right){wvv}_{j,2}\left( t \right).$$

$$\frac{{devb}_{j,1}}{dt}= d_{j-1}\left( {1-\delta}_{1,j} \right)\left( 1-\theta_{j}^{b}\left( t \right)-\theta_{j}^{d}\left( t \right) \right){evb}_{j-1,1}\left( t \right)+\lambda_{j}\left( t \right){vs}_{j,1}\left( t \right)- \left( d_{j}+\mu_{j}+\epsilon^{vb} \right){evb}_{j,1}\left( t \right).$$

$$\frac{{devb}_{j,2}}{dt}= d_{j-1}\left( {1-\delta}_{1,j} \right){evb}_{j-1,2}\left( t \right)+\lambda_{j}\left( t \right){vs}_{j,2}\left( t \right)+d_{j-1}\left( {1-\delta}_{1,j} \right)\left( \theta_{j}^{b}\left( t \right)+\theta_{j}^{d}\left( t \right) \right){evb}_{j-1,1}\left( t \right)- \left( d_{j}+\mu_{j}+\epsilon^{vb} \right){evb}_{j,2}\left( t \right).$$

$$\frac{{divb}_{j}}{dt}= d_{j-1}\left( {1-\delta}_{1,j} \right){ivb}_{j-1}\left( t \right)+\epsilon^{vb}\left( {evb}_{j,1}\left( t \right)+{evb}_{j,2}\left( t \right) \right)-\left( d_{j}+\mu_{j}+\gamma^{vb}+d_{j}^{vb} \right){ivb}_{j}\left( t \right).$$

$$\frac{{drvb}_{j}}{dt}= d_{j-1}\left( {1-\delta}_{1,j} \right){rvb}_{j-1}\left( t \right)+\left( {\xi^{vb}}_{j}+{\zeta^{vb}}_{j}\lambda_{j}\left( t \right) \right){wvb}_{j}\left( t \right)+\gamma^{vb}{ivb}_{j}\left( t \right)-\left( d_{j}+\mu_{j}+\delta^{vb} \right){rvb}_{j}\left( t \right).$$

$$\frac{{dwvb}_{j}}{dt}= d_{j-1}\left( {1-\delta}_{1,j} \right)\left( 1-\theta_{j}^{z}\left( t \right) \right){wvb}_{j-1}\left( t \right)+\delta^{vb}{rvb}_{j}\left( t \right)-\left( d_{j}+\mu_{j}+\chi\sigma_{j}+{\xi^{vb}}_{j}+{\zeta^{vb}}_{j}\lambda_{j}\left( t \right) \right){wvb}_{j}\left( t \right).$$

$$\frac{{dz}_{j}}{dt}= \sigma_{j}w_{j}\left( t \right)+d_{j-1}\left( {1-\delta}_{1,j} \right)z_{j-1}\left( t \right)-\left( d_{j}+\mu_{j}+\eta^{n}+d_{j}^{z} \right)z_{j}\left( t \right).$$

$$\frac{{dzvacc}_{j}}{dt}=d_{j-1}\left( {1-\delta}_{1,j} \right){zvacc}_{j-1}\left( t \right)+\chi\sigma_{j}\left( {wvv}_{j,1}\left( t \right)+{wvv}_{j,2}\left( t \right) \right)-\left( d_{j}+\mu_{j}+\eta^{vv}+d_{j}^{z} \right){zvacc}_{j}\left( t \right).$$

$$\frac{{dzwild}_{j}}{dt}=d_{j-1}\left( {1-\delta}_{1,j} \right){zwild}_{j-1}\left( t \right)+\chi\sigma_{j}{wvb}_{j}\left( t \right)-\left( d_{j}+\mu_{j}+\eta^{vb}+d_{j}^{z} \right){zwild}_{j}\left( t \right).$$

$$\frac{{drz}_{j}}{dt}=d_{j-1}\left( {1-\delta}_{1,j} \right){rz}_{j-1}\left( t \right)+\eta^{n}z_{j}\left( t \right)+\eta^{vb}{zwild}_{j}\left( t \right)+\eta^{vv}{zvacc}_{j}\left( t \right)-\left( d_{j}+\mu_{j} \right){rz}_{j}\left( t \right).$$

$$\frac{{dndv}_{j}}{dt}=d_{j-1}\left( {1-\delta}_{1,j} \right){ndv}_{j-1}\left( t \right)+d_{j}^{v}i_{j}\left( t \right)+d_{j}^{vb}{ivb}_{j}\left( t \right)-\left( d_{j}+\mu_{j} \right){ndv}_{j}\left( t \right).$$

$$\frac{{dndz}_{j}}{dt}=d_{j-1}\left( {1-\delta}_{1,j} \right){ndz}_{j-1}\left( t \right)+d_{j}^{z}\left( z_{j}\left( t \right)+{zwild}_{j}\left( t \right)+{zvacc}_{j}\left( t \right) \right)-\left( d_{j}+\mu_{j} \right){ndz}_{j}\left( t \right).$$

$$\lambda_{j}\left( t \right)=\sum_{a} \beta_{j,a}\left( i_{a}\left( t \right)+\rho^{v}{ivb}^{a}\left( t \right)+\rho^{z}z_{a}\left( t \right)+\rho^{z}{zwild}_{a}\left( t \right)+\rho^{z}{zvacc}_{a}\left( t \right) \right).$$

$$B^{m}\left( t \right)=\sum_{j} f_{j}n_{j}- \sum_{j} \left( f_{j}s_{j}\left( t \right)+\sum_{l} f_{j}s_{j,l}\left( t \right) \right).$$

$$B^{s}\left( t \right)= \sum_{j} \left( f_{j}s_{j}\left( t \right)+\sum_{l} f_{j}s_{j,l}\left( t \right) \right).$$

For all strategies

$$\theta_{j}^{c}\left( t \right)=\theta_{j}^{d}\left( t \right)=\theta_{j}^{s}\left( t \right)=0,$$

$$\theta_{j}^{p}\left( t \right)=\left\{ \begin{matrix} sigFun\left( vcr_{initial}^{1},vcr_{equilibrium}^{1},adoption_{year}^{1},adoption_{speed}^{1},t \right) & j=5 \\ 0 & j\neq5 \end{matrix} \right., and$$

$$\theta_{j}^{b}\left( t \right)=\left\{ \begin{matrix} sigFun(vcr_{initial}^{2},vcr_{equilibrium}^{2},adoption_{year}^{2},adoption_{speed}^{2},t) & j=20 \\ 0 & j\neq20 \end{matrix} \right. .$$

In words, the fraction of eligible 1-year-olds receiving varicella vaccination, i.e., the first-dose vaccine uptake, was parameterized by the following time-varying sigmoidal function:

$$sigFun\left( vcr_{initial}^{1},vcr_{equilibrium}^{1},adoption_{year}^{1},adoption_{speed}^{1},t \right)=\left\{ \begin{matrix} 0 & if t<2003 \\ vcr_{equilibrium}^{1}\times\Phi\left( t \right)+vcr_{initial}^{1}\times\left( 1-\Phi\left( t \right) \right) & otherwise \end{matrix} \right. ,$$

where $vcr_{initial}^{1}<vcr_{equilibrium}^{1}$, where $\Phi\left( t \right)$ is the cumulative distribution function of a normal random variable with mean $adoption_{year}^{1}$ and standard deviation $adoption_{speed}^{1}$ evaluated at time $t$, and where we have assumed null VCR prior to 2003. The interpretation of the parameters of $sigFun$ are as follows: $vcr_{initial}^{1}$ determines the initial first-dose vaccine uptake, $vcr_{equilibrium}^{1}$ determines the long-term equilibrium first-dose uptake as $t\to\infty$, $adoption_{year}^{1}$ determines the year where first-dose uptake experiences the fastest growth rate, and $adoption_{speed}^{1}$ determines the steepness of the increase in first-dose uptake over time. Analogously, the fraction of eligible 5-year-olds receiving varicella vaccination was parameterized by

$sigFun\left( {vcr}_{initial}^{2},vcr_{equilibrium}^{2},{adoption}_{year}^{2},{adoption}_{speed}^{2},t \right) .$

# **S1.4 Calibration Steps for Vaccine Coverage Parameters**

## ***S1.4.1 Epidemiological Parameters***

The epidemiological model parameters determined through calibration, i.e., the parameters determined through calibration in the no-vaccination model, are summarized in Table S1.4. These parameters are calibrated using a single set of national-level ODEs that are calibrated against national-level seroprevalence, HZ incidence, and hospitalization data. We note that calibration of epidemiological parameters is the only context in which a single national-level ODE model is simulated. In all other contexts, national level results are computed by aggregating regional results.

Epidemiological parameters can be classified into four categories. First, we have the relative risk of parameters for age groups 0-3 ($rr[1]$), 4-19 ($rr[2]$), and 20+ ($rr[3]$), which are multiplying factors that modify the force of infection. Second, we have the waning rate for natural maternal immunity ($\omega m$). Third, we have the parameters for reactivation rates ($\omega$, $\phi$, $\eta$, $\pi$). We note that the reactivation rate for age *a* is assumed to be given by the “bathtub function”

$$\sigma\left( a \right)=\omega e^{-\phi a}+\frac{\pi a^{\eta}}{100 000} .$$

Finally, the probability of hospitalization per varicella infection has been assumed to vary by age and is given by a continuous piecewise linear function for the age groups <1, 1-4, 5-24, and 25+. This parameterization is done with five parameters $h[0]$, $h[1]$, $h[5]$, $h[25]$, and $h[100]$, corresponding to the probability of hospitalization per varicella infection for ages 0, 1, 5, 25, and 100.

**Table S1.4: Calibrated parameters for VZV DTM (excluding demographic parameters)**

| **Symbol** | **Description** |
| --- | --- |
| $rr\left[ 1 \right]$ | Relative risk for ages 0-3 |
| $rr\left[ 2 \right]$ | Relative risk for ages 4-19 |
| $rr\left[ 3 \right]$ | Relative risk for ages 20+ |
| $\omega m$ | Natural maternal immunity waning rate |
| $\omega$ | Reactivation rate parameter for “bathtub function” |
| $\phi$ |  |
| $\eta$ |  |
| $\pi$ |  |
| $h\left[ 0 \right]$ | Probability of hospitalization per varicella case for age 0 |
| $h\left[ 1 \right]$ | Probability of hospitalization per varicella case for age 1 |
| $h\left[ 5 \right]$ | Probability of hospitalization per varicella case for age 5 |
| $h\left[ 25 \right]$ | Probability of hospitalization per varicella case for age 25 |
| $h\left[ 100 \right]$ | Probability of hospitalization per varicella case for age 100 |

Optimization proceeded in four steps. First, assuming an initial guess for HZ reactivation parameters, a binomial log-likelihood objective function for $rr\left[ j \right]$ (relative risk) and $\omega m$ (waning rate of maternal immunity) parameters was formed using the seroprevalence data [11, 12]. These parameters were estimated by maximizing the log-likelihood objective function. Second, using the $rr\left[ j \right]$ and $\omega m$ from the last step, a binomial log-likelihood objective function was formed for the reactivation parameters ($\omega$, $\phi$, $\eta$, $\pi$) using the HZ incidence data ( [13, 14]). These parameters were fit by maximizing the log-likelihood function. Third, using the $rr\left[ j \right]$, $\omega m$, and bathtub parameters ($\omega$, $\phi$, $\eta$, $\pi$) found above, a binomial log-likelihood objective function for hospitalizations was formed for the hospitalization parameters (h[0], h[1], h[5], h[25], h[100]) using hospitalization incidence data [15]. These parameters were fit by maximizing the log-likelihood function. Finally, the calibrated parameters found in above steps were used to identify an initial search region for a master binomial log-likelihood objective function (i.e., the objective function equal to the sum of the three log-likelihood functions described above). In this last step, the master objective function was optimized with respect to all parameters simultaneously.

All optimizations were done with the Nelder-Mead algorithm as implemented by the NMinimize routine in Mathematica 13.1.

**Figure S1.2 National-level model calibration and validation results**


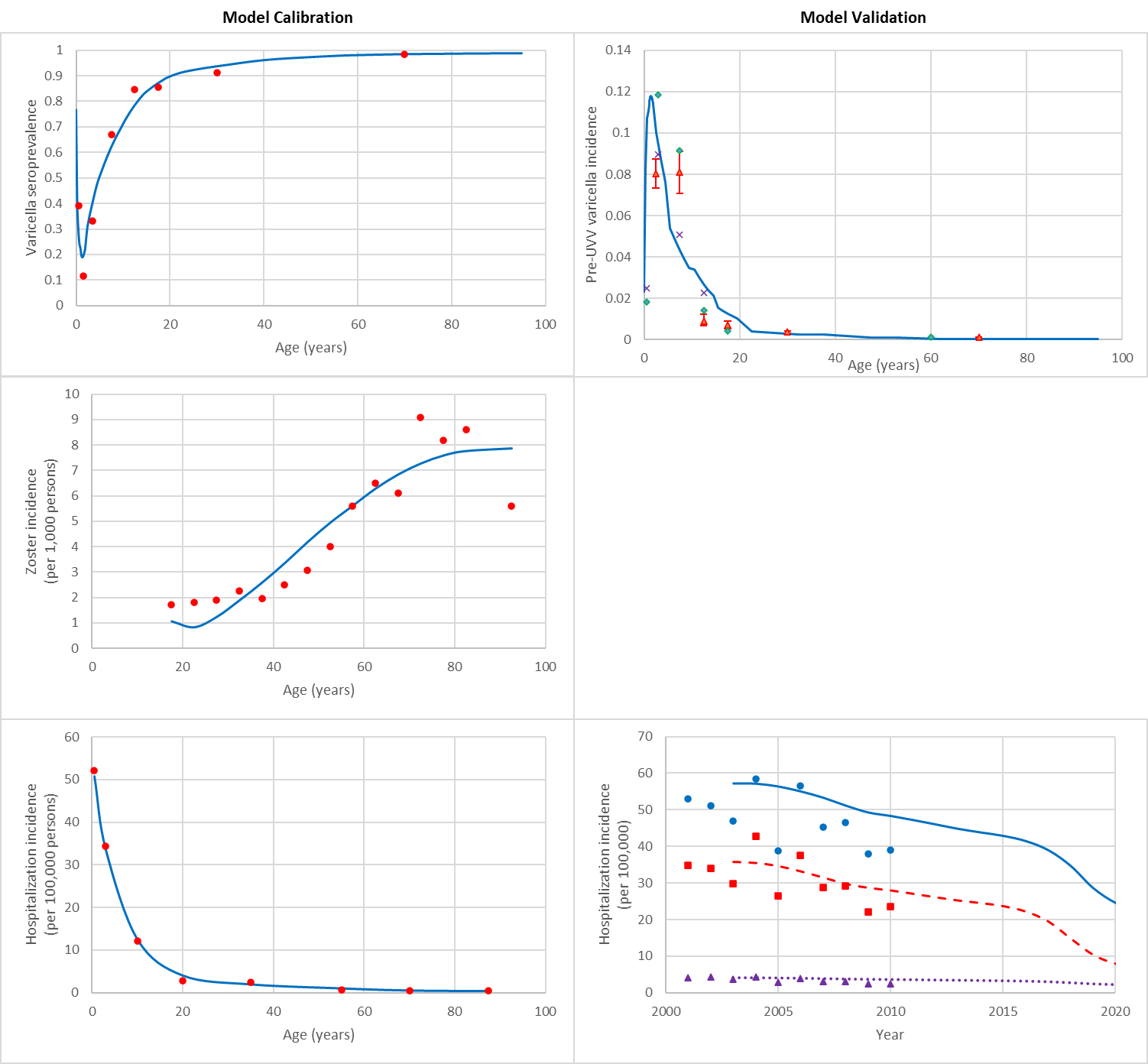


**Note 1**: (Top) Varicella seroprevalence and incidence. (Top left) Varicella seroprevalence for (line) model output versus (circles) calibration data. (Top right) Varicella incidence for (line) model output and (red triangles; bars indicate 95% CI) Riera-Montes, et al. (2017), (green triangles) Baldo, et al. (2009), (purple exes) Atti, et al. (2002).

**Note 2**: (Middle) Zoster incidence for (line) model output versus (circles) calibration data.

**Note 3:** (Bottom) Varicella hospitalization. (Bottom left) Varicella hospitalization incidence for (line) model output and calibration data (circles). (Bottom right) Varicella hospitalizations by age and time for (blue) <1-year-olds, (red) 1-4-year-olds, and (purple) total population. Calibration data are denoted by symbols; model output are denoted by lines.

## ***S1.4.2 Vaccine Coverage Parameters***

The parameters $vcr_{initial}$, $vcr_{equilibrium}$, $adoption_{year}$, and $adoption_{speed}$ were calibrated in two steps. In the first step, the parameter $vcr_{equilibrium}$, i.e., the asymptotic varicella uptake as $t\to\infty$, was calibrated. In the second step, the remaining parameters were considered.

In the first step $vcr_{initial}^{1}=vcr_{initial}^{2}=0$, $adoption_{year}^{1}=adoption_{year}^{2}=2003$, and $adoption_{speed}^{1}=adoption_{speed}^{2}=0.125$ were assumed. The parameter $vcr_{equilibrium}^{1}$ is interpreted to be the asymptotic 1-dose varicella coverage/uptake rate. The expression $vcr_{equilibrium}^{1}\times vcr_{equilibrium}^{2}$ is interpreted to be the asymptotic 2-dose varicella coverage rate. The average 2-dose MMR coverage for 5–6-year-olds for 2013–2019 was assumed as representative of the asymptotic 2-dose varicella coverage. Similarly, the maximum of the averages of the 1-dose MMR coverage for 2-year-olds (2013–2019) and 4-year-olds (2017–2019) was assumed as the representative of the asymptotic 1-dose varicella coverage. Optimization in the first step proceeds by minimizing the sum of squared differences between the quantities described above. We note that the situation for 1-dose varicella coverage is more complex than for 2-dose varicella coverage because in our model the first dose in the MMRV vaccination series is given in the first year of life, whereas in the real world the first dose in the MMRV vaccination series can be given up to at least the fourth year of life. For example, the average difference between the first dose MMR vaccine coverage in Italy for 4-year-olds and 2-year-olds is 5.36%: 93.46% (4-year-olds; 2017-2019) versus 88.09% (2-year-olds; 2015-2017).

In the second step, $vcr_{initial}^{2}=0$, $adoption_{year}^{2}=2003$, and $adoption_{speed}^{2}=0.125$ were assumed. We assume that the 2-dose varicella VCRs for 5–6-year-olds are the most reliable vaccine coverage data, i.e., they are more reliable than the 1-dose varicella vaccine coverage at 2- and 4-years-old, since 2-dose varicella vaccination is a prerequisite for enrolling in school [16]. The calibration in this step proceeds by minimizing the sum of squared differences between the second-dose varicella coverage subject to a penalty that penalizes parameter combinations that predict first-dose varicella coverage rates lower than observed first-dose varicella coverage rates for 2-year-olds. Varicella VCRs at 4-years-old are held in reserve for the purposes of model validation. Specifically, for first-dose vaccine coverage data for 2-year-olds given by $y_{i}$, second-dose vaccination coverage data for 5–6-year-olds by $x_{i}$, model output for first-dose vaccine coverage for 1-year-olds by $\hat{y}_{i}$, and model output for second-dose vaccine coverage for 5-year-olds by $\hat{x}_{i}$, the following objective function was applied:

$$\sum_{i} \left( x_{i}-\hat{x}_{i} \right)^{2}+\left( y_{i}-\hat{y}_{i-1} \right)^{2}\mathbb{\times I}_{\left\{ y_{i}>\hat{y}_{i-1} \right\}}$$

Note that, since the varicella vaccine coverage data are given for 2-year-olds, the indices for model outputs for first-dose vaccine coverage for 1-year-olds has been shifted downward by one year (i.e., we use $\hat{y}_{i-1}$ as our model output in the objective function).

**Table S1.5: Description of parameters used in sigmoidal function** $\boldsymbol{sigFun}$

| **Parameter** | **Description** |
| --- | --- |
| $vcr_{initial}$ | For small $adoption_{speed}$, the parameter $vcr_{initial}$ is the asymptotic uptake rate as $t\to{2003}^{+}$. |
| $vcr_{equilibrium}$ | The parameter $vcr_{equilibrium}$ is the asymptotic vaccine uptake rate as $t\to\infty$. |
| $adoption_{year}$ | The parameter $adoption_{year}$ is the year where vaccine uptake rate experiences the fastest rate of growth. |
| $adoption_{speed}$ | The parameter $adoption_{speed}$ controls the steepness of the vaccine uptake rate over time, i.e., it should take roughly $4\times adoption_{speed}$ years for vaccine coverage to reach $vcr_{equilibrium}$. |

**Table S1.6: Average MMR VCR and fitted equilibrium varicella VCR parameters**

| **Region** | **First dose** | | | **Second dose** | |
| --- | --- | --- | --- | --- | --- |
|  | **Average MMR VCR** | | **First-dose uptake (proxy for 1^st^-dose VCR;**  $\boldsymbol{vc}\boldsymbol{r}_{\boldsymbol{equillibrium}}^{\boldsymbol{1}}$**)** | **Average MMR VCR at 5 years (2013-2019 data; 2008-2014 birth cohorts)** | **Product of first- and second-dose uptake**  **(proxy for 2^nd^-dose VCR;**  $\boldsymbol{vc}\boldsymbol{r}_{\boldsymbol{equilibrium}}^{\boldsymbol{1}}\boldsymbol{\times vc}\boldsymbol{r}_{\boldsymbol{equilibrium}}^{\boldsymbol{2}}$**)** |
|  | **24 months (2013-2019 data;  2011-2017 birth cohorts)** | **48-month (2017-2019 data; 2013-2015 birth cohorts)** |  |  |  |
| Abruzzo | 89.6% | 92.5% | 93.7% | 88.3% | 93.7% |
| Basilicata | 91.5% | 96.3% | 97.2% | 87.5% | 92.2% |
| Calabria | 88.6% | 86.9% | 90.3% | 74.9% | 81.1% |
| Campania | 88.1% | 87.5% | 89.9% | 76.7% | 83.5% |
| Emilia Romagna | 90.8% | 94.6% | 95.6% | 89.9% | 95.0% |
| Friuli Venezia Giulia | 86.7% | 93.0% | 94.1% | 88.0% | 93.3% |
| Lazio | 91.1% | 94.6% | 95.7% | 84.7% | 89.8% |
| Liguria | 87.2% | 93.6% | 94.7% | 83.6% | 88.6% |
| Lombardia | 92.7% | 94.9% | 95.9% | 90.2% | 95.5% |
| Marche | 86.6% | 92.9% | 94.0% | 87.7% | 93.1% |
| Molise | 85.8% | 91.6% | 92.9% | 79.4% | 84.8% |
| Piemonte | 92.5% | 95.6% | 96.5% | 91.0% | 95.9% |
| Prov. Auton. Bolzano | 70.3% | 84.4% | 86.6% | 70.6% | 78.0% |
| Prov. Auton. Trento | 89.1% | 94.5% | 95.6% | 88.8% | 94.1% |
| Puglia | 89.3% | 95.3% | 96.3% | 84.3% | 89.2% |
| Sardegna | 90.9% | 94.4% | 95.3% | 88.1% | 93.0% |
| Sicilia | 86.0% | 94.5% | 95.9% | 69.2% | 74.1% |
| Toscana | 91.7% | 94.2% | 95.2% | 86.7% | 91.8% |
| Umbria | 91.9% | 96.0% | 96.9% | 92.2% | 96.9% |
| Valle d'Aosta | 85.6% | 93.8% | 94.9% | 88.0% | 93.4% |
| Veneto | 90.6% | 94.0% | 95.0% | 87.7% | 93.0% |
| **Italy** | **89.9%** | **93.5%** | **94.8%*** | **84.7%** | **90.1%*** |

*** Table rows are in descending order with respect to fitted value of${vcr}_{equilibrium}^{1}\times{vcr}_{equilibrium}^{2}$

# **S1.5 Model Inputs (QALYs and Cost)**

## ***S1.5.1 Epidemiological inputs***

**Table S1.7: Epidemiological parameters**

| **Description** | **Value** | **Source** |
| --- | --- | --- |
| **Natural varicella infection inputs** | | |
| Average waning period of passive (maternally induced) immunity | 4.25 months | Calibrated |
| Average latent period of natural and breakthrough varicella | 14 days | [17, 18] |
| Average infectious period of natural varicella | 7 days | [17, 19, 20] |
| Average waning period of zoster immunity after natural and breakthrough varicella | 81.3 years | [21] |
| **Breakthrough varicella infection inputs** | | |
| Relative infectivity of breakthrough varicella | 50% | [18, 22] |
| Average infectious period of breakthrough varicella | 6 days | [17, 18, 19] |
| Average waning period of exogenously boosted zoster immunity following successful varicella vaccination | 81.3 years | [21]  Assumed same as natural varicella |
| **HZ inputs** | | |
| Relative infectivity of HZ | 7% | [23, 24] |
| Relative reactivation rate for zoster on vaccine arms | 1/6 | [25] |
| Average duration of HZ outbreak following natural and breakthrough varicella | 28 days | [23, 26] |
| Average duration of HZ outbreak following successful varicella vaccination | 28 days | [23] |
| **Exogenous boosting inputs** | | |
| Proportion of contacts leading to exogenous boosting after natural/breakthrough varicella | 33.45% | [27] |
| Proportion of contacts leading to exogenous boosting after varicella vaccination | 33.45% | [27] |
| **Disease related death inputs** | | |
| Natural varicella infection-related death rate (per year) | Multiple | [7, 28] |
| Breakthrough varicella infection-related death rate (per year) | 0.00 | Assumed |
| HZ reactivation-related death rate (per year) | Multiple | [29] |

## ***S1.5.2 Vaccine Efficacy***

Vaccine failure rates (defined as the proportion of individuals who did not seroconvert within 42 days of vaccine administration) were drawn directly from RCT results [30, 31, 32]. Duration of protection was previously estimated using deterministic compartmental models to simulate clinical trials of 1 or 2-dose varicella vaccination with MSD and GSK vaccines [33]. Specifically, it was estimated that 90.3% (95% CI: 87.8–92.9%) of the cohort gained permanent protection from breakthrough varicella after the first dose of MSD vaccines compared to 61.7% (95% CI: 58.2–65.3%) after the first dose of GSK vaccines. Inputs for additional vaccine properties (dose take rate and average waning period of high HZ immunity following vaccination) are provided in the table below.

**Table S1.8: Vaccine properties**

| **Parameter** | **Definition** | **Dose** | **MSD vaccines** | **GSK vaccines** | **Source** |
| --- | --- | --- | --- | --- | --- |
| $1-P$ | Vaccine failure rate | 1 & 2 | 4% | 5% | [31, 32] |
| $T_{l}$ | Dose take rate | 1^st^ | 90.3% | 61.7% | [33] |
|  |  | 2^nd^ | 69.0% | 83.4% | [33] |
|  |  | 1 & 2 | 97% | 93.8% | [33] |
| $\frac{1}{\sigma^{v}}$ | Average duration of protection | 1 & 2 | 1.2 years | 0.9 years | [33] |
| $\frac{1}{\pi_{l}}$ | Average waning period of high HZ immunity following vaccination | 1^st^ | 81.3 years | 81.3 years | [21] (Assumed to be same as natural varicella) |

In our model, GSK vaccines were implemented from 2003-2020, after which either GSK or MSD vaccines were implemented. Manufacturer switching at $t=2020$ was implemented by allowing vaccine parameters listed in Table S1.8 to be time varying, i.e., for strategies where MSD vaccines were implemented post-2020 the vaccine parameters took the functional form

$$parameter\left( t \right)=parameter_{GSK}\times\mathbb{I}_{\left\{ t<2020 \right\}}+parameter_{MSD}\times\mathbb{I}_{\left\{ t\geq2020 \right\}} .$$

The manufacturer switching implementation for this model contrasts that of Kujawski and colleagues [34], which implemented two sets of vaccine compartments (one for each brand). For our model we chose not to follow the Kujawski implementation [34], as the additional computational costs of this method (when combined with our regional modelling requirements) were prohibitive.

## ***S1.5.3 QALY inputs***

For individuals with natural varicella infection, a QALY loss of 0.004 and 0.005 per case for <15-year-olds and ≥15-year-olds, respectively was assumed [35]. Following Brisson and Edmunds et al., 0.001 QALYs are lost per breakthrough varicella infection [29]. The instantaneous QALY loss due to varicella infection for age groups was the sum of losses due to natural and breakthrough varicella. Utility values for healthy individuals were obtained from Scalone et al. [36], to align their age ranges with those used in our model and weighted averages (based on population size) of QALY weights across two age categories were calculated. Thus, the age-specific health state utility values were used to calculate QALYs lost.

**Table S1.9: QALYs for healthy individuals**

| **Age group**  **(years)** | **Female**  **population (%)** | **EQ-5D-5L** | | |
| --- | --- | --- | --- | --- |
|  |  | **Female** | **Male** | **Average** |
| <18 | 48.53% | 1.000 | 1.000 | 1.000 |
| 18-35 | 48.57% | 0.941 | 0.959 | 0.950 |
| 36-45 | 50.01% | 0.936 | 0.945 | 0.940 |
| 46-55 | 50.56% | 0.912 | 0.930 | 0.921 |
| 56-65 | 56.63% | 0.895 | 0.919 | 0.905 |
| 66-75 | 53.00% | 0.877 | 0.907 | 0.891 |
| 76+ | 60.15% | 0.829 | 0.879 | 0.849 |
| **Source** | [7] | [36] | | |

**Table S1.10: QALY loss for varicella infection**

| **Age group**  **(years)** | **QALY loss per case** | | **QALY weights** | |
| --- | --- | --- | --- | --- |
|  | **Natural varicella** | **Breakthrough varicella** | **Natural varicella**  **(**$\boldsymbol{q}_{\boldsymbol{j}}^{\boldsymbol{v}}$**)** | **Breakthrough varicella**  **(**$\boldsymbol{q}_{\boldsymbol{j}}^{\boldsymbol{vb}}$**)** |
| <15 | 0.004 | 0.001 | 0.791 | 0.939 |
| ≥15 | 0.005 | 0.001 | 0.739 | 0.939 |
| **Source** | [37] | Assumption | [35] | Assumption |

## ***S1.5.4 Cost inputs***

The list price of vaccines was reduced by subtracting VAT (10%) and a mandatory rebate (50%), consistent with [National Law N. 386/1974](https://www.gazzettaufficiale.it/eli/id/1974/08/29/074U0386/sg) [38]. The administration cost for monovalent and tetravalent vaccine was taken based on national tariff [39].

**Table S1.11: Vaccination costs**

| **Quantity** | **List Price**  **(€)** | **Cost***  **(= Price / 2 / 1.1; €)** | **Source** |
| --- | --- | --- | --- |
| **MSD** | | | |
| M-M-RvaxPro® | 30.50 | 13.86 | [40] |
| Vx | 84.15 | 38.25 | [40] |
| PQ | 114.65 | 52.11 | [40] |
| **GSK** | | | |
| PRIORIX | 26.10 | 11.86 | [40] |
| Vr | 86.50 | 39.32 | [40] |
| PT | 114.65 | 52.11 | [40] |
| **Administration** | | | |
| Monovalent | N/A | 6.95 per dose | National tariff [39] |
| Tetravalent | N/A | 6.95 per dose | National tariff [39] |

**Abbreviation:** Vx: VARIVAX®, PQ: ProQuad®, Vr: VARILRIX®, PT: PRIORIX TETRA.

**Note:** PRIORIX and M-M-RvaxPRO® are both MMR vaccines. *In the calculation cost = price / 2/ 1.1, where 1.1 is used for the removal of VAT (divide by 1.1, i.e., 10%) and 50% mandatory rebate has been applied by dividing the price by 2.

**Table S1.12: Vaccination costs by strategy for years 2020 onward**

| **Strategy** | **Component** | **Cost (€)** | |
| --- | --- | --- | --- |
|  |  | **Dose 1**  **(**$\boldsymbol{vpc}$**)** | **Dose 2**  **(**$\boldsymbol{vbc}$**)** |
| A (MMRV-MSD/MMRV-MSD) | MMRV-MSD | 38.25 | 38.25 |
|  | Marginal administration fee | 0 | 0 |
|  | Adverse events | 0.40 | 0 |
|  | Total | 38.65 | 38.25 |
| B (MMRV-GSK/MMRV-GSK) | MMRV-GSK | 40.25 | 40.25 |
|  | Marginal administration fee | 0 | 0 |
|  | Adverse events | 0.40 | 0 |
|  | Total | 40.65 | 40.25 |
| C (V-MSD/MMRV-MSD) | M-M-R vaxPro® and Vx | 38.25 | 0 |
|  | MMRV- MSD | 0 | 38.25 |
|  | Marginal administration fee | 6.95 | 0 |
|  | Adverse events | 0 | 0 |
|  | Total | 45.20 | 38.25 |
| D (V-GSK/MMRV-GSK) | PRIORIX and Vr | 39.32 | 0 |
|  | MMRV-GSK | 0 | 40.25 |
|  | Marginal administration fee | 6.95 | 0 |
|  | Adverse events | 0 | 0 |
|  | Total | 46.27 | 40.25 |

Direct inpatient costs were assumed to be the same for natural and breakthrough varicella. However, the probability of seeking inpatient care for breakthrough varicella infections was assumed to be 20% of the probability of seeking inpatient care for natural varicella infections. Outpatient indirect costs for breakthrough varicella are assumed to be 86% of the outpatient indirect costs for natural varicella.

**Table S1.13: Drug costs by age (2022 currency)**

| **Age group (years)** | **Cost (€)** | **Source** |
| --- | --- | --- |
| **<15** | | |
| Antihistamine | (5 + 9.32)/2 = 7.16 | Italian Medicines Agency [41] |
| Paracetamol | 4.00 | Italian Medicines Agency [41] |
| **Total** | **11.16** |  |
| **15+** | | |
| Antihistamine | (5 + 9.32)/2 = 7.16 | Italian Medicines Agency [41] |
| Paracetamol | 7.00 | Italian Medicines Agency [41] |
| Acyclovir | 25.04 | Italian Medicines Agency [41] |
| **Total** | **39.20** |  |

**Table S1.14: Summary of outpatient cost data (2022 currency)**

| **Description** | **Value** | **Source** |
| --- | --- | --- |
| Number of outpatient visits for individuals who require at least one outpatient visit | 1 | Assumption |
| Cost per outpatient visit stratified by age | <15-year-olds: 29.00 €  ≥15-year-olds: 23.31 € | Barbieri, et al. (2022) [42]  [39] (Code: 89.7) |
| Fraction of outpatient cases that require prescription or OTC drugs | 90% | Assumption,  Expert opinion |
| Total drug cost per outpatient case | <15-year-olds: 11.16 €  ≥15-year-olds: 39.20 € | See text for details |
| Fraction of outpatient cases that require diagnostic testing | 1% | Assumption,  Expert opinion |
| Cost of diagnostic testing | 40.96 € | [39], see text for details. |
| Productivity loss (days) per varicella infection stratified by age | <15-year-olds: 0.7 days  15 – 64-year-olds: 5.7 days  ≥65-year-olds: 0 days | [43, 44, 45]  [43, 46]  Assumption |

# **S1.6 Distributions in PSA Analysis for Cost and QALY Parameters**

**Table S1.15: Parameter and values used in the sensitivity analyses**

| **Description** | **Distribution** | | |
| --- | --- | --- | --- |
|  | **Type** | **Mean**  **(Base case value)** | **Standard**  **deviation** |
| **Epidemiology and inpatient resource use parameters** | | | |
| Relative risk for 0 – 3-year-olds | Estimated from posterior sampling of history matching calibration procedure | 4.3888 | Calculated from optimization procedure |
| Relative risk for 4 – 19-year-olds |  | 4.3510 |  |
| Relative risk for ≥20-year-olds |  | 2.0466 |  |
| Waning rate for maternal immunity |  | 2.8244 |  |
| Reactivation rate parameter for “bathtub function” |  | 59.3584 |  |
|  |  | 0.4740 |  |
|  |  | 1.5982 |  |
|  |  | 4.8121 |  |
| Per case probability of hospitalization for age 0 |  | 0.007590 |  |
| Per case probability of hospitalization for age 1 |  | 0.004057 |  |
| Per case probability of hospitalization for age 5 |  | 0.002931 |  |
| Per case probability of hospitalization for age 25 |  | 0.005738 |  |
| Per case probability of hospitalization for age 100 |  | 0.020888 |  |
| **Vaccine coverage rate (VCR) parameters** | | | |
| $vcr_{initial}^{1}$ | Uniform | Various values,  See below for details | |
| $vcr_{equilibrium}^{1}$, $vcr_{equilibrium}^{2}$ | Beta, Triangular |  |  |
| $adoption_{year}^{1}$ | Normal |  |  |
| $adoption_{speed}^{1}$ | Lognormal |  |  |
| **Herpes-zoster-related parameters** |  |  | |
| Proportion of contacts leading to exogenous boosting | Beta | 0.3345 | 0.0669** |
| Relative reactivation rate for varicella vaccinated individuals | Beta | 0.1667 | 0.0333** |
| Recovery rate for HZ outbreak | Lognormal | 13.04 | 2.61** |
| Relative infectiousness of herpes zoster | Beta | 0.070 | 0.014** |
| **Cost- and QALY-related parameters** |  |  |  |
| Multiplying factor for direct treatment costs | Lognormal | 1 | 0.2** |
| Multiplying factor for indirect treatment costs | Lognormal | 1 | 0.2** |
| Multiplying factor for direct vaccination costs | Lognormal | 1 | 0.2** |
| Multiplying factor for healthy QALY parameters ($q_{j}^{h}$) | Beta***, see text below | 1 | 0.05* |
| Multiplying factor for natural varicella QALY loss parameters ($q_{j}^{v}$) | Beta***, see text below | 1 | 0.05* |
| Multiplying factor for breakthrough varicella QALY loss parameters ($q_{j}^{vb}$) | Beta***, see text below | 1 | 0.05* |
| **Vaccine-related parameters (MSD-specific)** | | | |
| Fraction protected following first dose/  Take rate following first dose | Beta | 0.903 | 0.014 |
| Fraction protected following two doses | Beta | 0.970 | 0.008 |
| Dirichlet distribution for $T1$, $X2-T1$ | Dirichlet | (0.903, 0.067) | (0.014, 0.012) |
| Take rate following second dose | Computed | 0.691 | 0.068 |
| Vaccine success rate ($1-Vaccine failure rate$) | Beta | 0.960 | 0.0480* |
| Waning rate for temporary vaccine immunity | Truncated Normal | 0.834 | 0.241 |
| Fraction protected following first dose/  Take rate following first dose | Beta | 0.617 | 0.018 |
| **Vaccine-related parameters (GSK-specific)** | | | |
| Fraction protected following two doses | Beta | 0.938 | 0.009 |
| Dirichlet distribution for $T1$, $X2-T1$ | Dirichlet | (0.617, 0.321) | (0.018, 0.017) |
| Take rate following second dose | Computed | 0.838 | 0.022 |
| Vaccine success rate ($1-Vaccine failure rate$) | Beta | 0.950 | 0.0475* |
| Waning rate for temporary vaccine immunity | Truncated Normal | 1.111 | 0.157 |
| **Vaccine-related parameters (constant between MSD and GSK)** | | | |
| Waning rate for durable vaccine immunity | Lognormal | 0.012 | 0.002** |
| Relative infectiousness for breakthrough varicella | Beta | 0.500 | 0.100** |

Ɨ Bound for search region, * Standard deviation assumed to be equal to 5% of the base case value, ** Standard deviation assumed to be equal to 20% of the base case value, *** Proportional to a beta distributed random variable, T1, dose-1 vaccine take rate; X2-T1, difference between total take rate and dose-1 take rate

## ***S1.6.1 Calibrated vaccine coverage parameters***

Calibrated vaccine coverage parameters include $vcr_{initial}^{1}$, $vcr_{equilibrium}^{1}$, $adoption_{year}^{1}$, $adoption_{speed}^{1}$, and ${vcr}_{equilibrium}^{2}$. We assume that $vcr_{initial}^{1}$ is uniformly distributed on the interval $\left[ 0,0.05 \right]$. For both $vcr_{equilibrium}^{1}$ and $vcr_{equilibrium}^{2}$ with calibrated value less than 1 we assume a Beta distribution with mean given by their calibrated value and with standard deviation equal to 0.05. For both $vcr_{equilibrium}^{1}$ and $vcr_{equilibrium}^{2}$ with calibrated value equal to 1 we assume a triangular distribution with mode 1 and support $[0.955,1]$ (i.e., variance is ${0.05}^{2}=0.00025$). We assume that $adoption_{year}^{1}$ is distributed as a Normal random variable with mean given by its calibrated value and with standard deviation equal to 1. Finally, we assume that $adoption_{speed}^{1}$ follows a lognormal distribution with mean given by their calibrated value and with standard deviation equal to 5% of their calibrated value.

## ***S1.6.2 Herpes zoster related parameters***

We include the following herpes zoster related parameters: the proportion of contacts leading to exogenous boosting ($\zeta$, assuming $\zeta=\zeta^{n}=\zeta^{vb}=\zeta^{vv}$), the relative reactivation rate for varicella vaccinated individuals ($\chi$), the recovery rate for HZ outbreaks ($\eta$, assuming $\eta=\eta^{n}=\eta^{vb}=\eta^{vv}$), and the relative infectiousness of HZ ($\rho^{z}$). Since $\zeta$, $\chi$, and $\rho^{z}$ all have support $[0,1]$, we assume that these are Beta random variables with mean given by their base case value and with standard deviation given by 20% of their base case value. We assume that the duration of HZ outbreaks is lognormally distributed, and hence, that the recovery rate for HZ outbreaks is also lognormally distributed. Specifically, we assume that $\eta$ is lognormally distributed with mean given by the base case value and with standard deviation equal to 20% of the base case value.

## ***S1.6.3 Cost and QALY parameters***

The model employs numerous cost and QALY inputs. To simplify the sensitivity analyses, we restrict our attention to six cost and QALY multiplying factors which are used to simulate uncertainty in cost and QALY categories. Specifically, we consider the following variables:

- $directTreatCostFactor$: multiplying factor for direct treatment costs,
- $indirectTreatCostFactor$: multiplying factor for indirect treatment costs,
- $directVaxCostFactor$: multiplying factor for vaccination costs,
- $qalyHealthyFactor:$ multiplying factor for healthy QALY parameters ($q_{j}^{h}$),
- $qalyNaturalFactor$: multiplying factor for natural varicella QALY parameters ($q_{j}^{v}$), and
- $qalyBreakthroughFactor$: multiplying factor for breakthrough varicella QALY parameters ($q_{j}^{vb}$).

We note that this model does not account for indirect costs of vaccination, and hence, there is no multiplying factor associated with indirect vaccination costs.

For cost multipliers we take the simple approach of assuming that cost multipliers are distributed lognormally with mean 1 and variance 0.2. For QALY multipliers, a more sophisticated approach is needed, since QALYs should not exceed 1. Specifically, for healthy QALYs ($q_{j}^{h}$) we assume that $qalyHealthyFactor$ is proportional to a Beta random variable with mean ${0.95}^{2}=0.903$ (i.e., the square of the QALY value for healthy 18–34-year-olds) and with standard deviation $0.05\times{0.95}^{2}=0.045$ (i.e., 5% of the mean). We choose the proportionality constant for $qalyHealthyFactor$ is equal to ${0.95}^{2}$ so that

1. $qalyHealthyFactor\in\left( 0,1 \right)$,
2. The mean of the product of $qalyHealthyFactor$ with the QALYs for healthy 18–34-year-olds is equal to the base case value for QALYs for healthy 18–34-year-olds, and
3. The standard deviation of the product of $qalyHealthyFactor$ with the QALYs for healthy 18 – 34-year-olds is equal to 5% of the base case value for QALYs of healthy 18–34-year-olds.

We take an analogous approach with $qalyNaturalFactor$ and $qalyBreakthroughFactor$, i.e., we assume

$$qalyNaturalFactor\sim\frac{Beta\left[ mean={0.791}^{2},\text{standard deviation}=0.05*{0.791}^{2} \right]}{{0.791}^{2}}$$

and

$$qalyBreakthroughFactor\sim\frac{Beta\left[ mean={0.939}^{2},\text{standard deviation}=0.05*{0.939}^{2} \right]}{{0.939}^{2}}$$

## ***S1.6.4 Vaccine parameters***

Vaccine parameters included in the sensitivity analysis comprise the vaccine failure rate ($P$), vaccine take rates ($T1$ – first dose; $T2$ – second dose), waning of temporary immunity ($\sigma^{v}$), waning of durable immunity ($\Pi=\pi1=\pi2$), and relative infectiousness of breakthrough varicella infections ($\rho^{v}$).

## ***S1.6.5 Vaccine failure rate***

We assume that the successful vaccination rate ($P=1-vaccine failure rate$) is Beta distributed with mean equal to the base case parameter value and with standard deviation equal to 5% of the base case parameter value.

## ***S1.6.6 Vaccine take-rates***

We introduce the notation $X2$ to represent the total take rate following both vaccine doses. We assume that the variables $T1$ (dose-1 take rate) and $X2-T1$ (difference between total take rate and dose-1 take rate) are Dirichlet distributed with parameters $a\times b$, $a\times(c-b)$, and $a\times(1-c)$. Here, $b$ is the base case value for $T1$, $c$ is the base case value for $X2$, and $a$ is determined through optimization. Given the above assumptions, we observe that T1 is distributed as a Beta distribution with parameters $a\times b$ and $a\times\left( 1-b \right)$ and $X2$ is distributed as a Beta distribution with parameters $a\times c$ and $a\times\left( 1-c \right)$. Thus, we choose $a$ to minimize the objective function

$$\left( \sqrt{Variance\left[ Beta\left[ a\times b,a\times\left( 1-b \right) \right] \right]}-\frac{diff^{95}(1)}{2\times1.96} \right)^{2}+\left( \sqrt{Variance[Beta[a\times c,a\times(1-c)]]}-\frac{diff^{95}\left( 2 \right)}{2\times1.96} \right)^{2} ,$$

where $diff^{95}\left( 1 \right)$ is the difference between upper and lower bounds for the 95% confidence interval of $T1$ and $diff^{95}\left( 2 \right)$ is the difference between the upper and lower bounds for the 95% confidence interval of $X2$. We use estimates of $T1$ and $X2$ (MSD: $0.903 [0.878,0.929]$ and 0.970 [0.952,0.988]; GSK: 0.617 [0.582,0.653] and 0.938 [0.922,0.954], respectively) from Pillsbury, et al. [33] Estimates of T1 and X2 are provided in Table S1.8.

To draw a parameter realization for T1 and T2 we generate the random variables

$$\left( T1,X2-T1 \right)\sim Dirichlet[a\times b,a\times\left( c-b \right),a\times\left( 1-c \right)]$$

and then compute

$$\left( T1,T2 \right)=\left( T1, \frac{X2-T1}{1-X1} \right) .$$

## ***S1.6.7 Waning of temporary immunity***

The 95% CI estimates of the waning rate for temporary immunity ($\sigma v$) for MSD and GSK products are roughly symmetrical. For example, for MSD products $\sigma v$ is estimated to be 0.826 with 95% CI $(0.351,1.299)$, i.e, $\sigma v\approx0.826\pm0.474$; analogously for GSK products, $\sigma v\approx0.826\pm0.308$. Thus, we model $\sigma v$ as a truncated Normal random variable with support $(0,\infty)$.

## ***S1.6.8 Waning of durable immunity***

We assume the same value for the waning of durable immunity for MSD and GSK products following either the first or second dose, i.e., $\Pi=\pi1=\pi2$. We further assume that $\pi$ is lognormally distributed with mean equal to the base case value and with standard deviation equal to 20% of the base case value.

## ***S1.6.9 Relative infectiousness of breakthrough varicella***

We assume that the relative infectiousness of varicella is the same for both MSD and GSK products. We further assume that this parameter is distributed as a Beta random variable with mean given by the base case parameter value and with standard deviation equal to 20% of the base case parameter value.

# **References**

| [1] | L. J. Wolfson, V. J. Daniels, M. Pillsbury, K. Kurugöl, C. Yardimci, J. Kyle and E. C. Dinleyici, "Cost-effectiveness analysis of universal varicella vaccination in Turkey using a dynamic transmission model," *PLOS ONE,* vol. 14, no. 8, 2019. |
| --- | --- |
| [2] | C. Azzari, V. Baldo, S. Giuffrida, R. Gani, E. O’Brien, C. Alimenti, V. J. Daniels and L. J. Wolfson, "The Cost-Effectiveness of Universal Varicella Vaccination in Italy: A Model-Based Assessment of Vaccination Strategies," *Clinicoeconomics and Outcomes Research,* vol. 12, p. 273, 2020. |
| [3] | J. Graham, L. J. Wolfson, J. Kyle, C. P. Bolde-Villarreal, D. B. Guarneros-DeRegil, H. Monsanto, M. Pillsbury, S. Talbird and V. J. Daniels, "Budget impact analysis of multiple varicella vaccination strategies: a Mexico perspective," *Human Vaccines & Immunotherapeutics,* vol. 16, no. 4, pp. 886-894, 2020. |
| [4] | M. Pawaskar, C. Burgess, M. Pillsbury, T. Wisløff and E. Flem, "Clinical and economic impact of universal varicella vaccination in Norway: A modeling study," *PLOS ONE,* vol. 16, no. 7, 2021. |
| [5] | C. Burgess, S. Samant, T. leFevre, C. Larsen and M. Pawaskar, "Universal varicella vaccination in Denmark: Modeling public health impact, age-shift, and cost-effectiveness," *PLOS Global Public Health,* vol. 3, no. 4, p. e0001743, 2023. |
| [6] | H. W. Hethcote, "The mathematics of infectious diseases," *SIAM Review,* vol. 42, no. 4, pp. 599-653, 2000. |
| [7] | Instituto Nazionale di Statistica, "I.Stat: Resident population on 1st January: By age," 1 1 2022. [Online]. Available: http://dati.istat.it/Index.aspx?QueryId=42869&lang=en. [Accessed 3 8 2022]. |
| [8] | Instituto Nazionale di Statistica, "I.Stat: Mother - Age and citizenship," 1 1 2022. [Online]. Available: https://www.istat.it/en/population-and-households?data-and-indicators. [Accessed 13 5 2022]. |
| [9] | Instituto Nazionale di Statistica, "I.Stat: Life tables: Age," 1 1 2022. [Online]. Available: http://dati.istat.it/Index.aspx?QueryId=19053&lang=en. [Accessed 3 8 2022]. |
| [10] | Instituto Nazionale di Statistica, "I.Stat: Demographic projections - Years 2020-2070," 2022. [Online]. Available: http://dati.istat.it/Index.aspx?QueryId=42869&lang=en. [Accessed 16 03 2022]. |
| [11] | A. De Donno, P. Kuhdari, M. Guido, M. C. Rota, A. Bella, G. Brignole, S. Lupi, A. Idolo, A. Stefanati, M. Del Manso and G. Gabutti, "Has VZV epidemiology changed in Italy? Results of a seroprevalence study," *Human Vaccines & Immunotherapeutics,* vol. 13, no. 2, pp. 385-90, 2017. |
| [12] | G. Gabutti, M. C. Rota, M. Guido, A. De Donno, A. Bella, M. L. Ciofi degli Atti and P. Crovari, "The epidemiology of Varicella Zoster Virus infection in Italy," *BMC Public Health,* vol. 8, no. 1, pp. 1-9, 2008. |
| [13] | L. E. Gialloreti, M. Merito, P. Pezzotti, L. Naldi, A. Gatti, M. Beillat, L. Serradell, R. di Marzo and A. Volpi, "Epidemiology and economic burden of herpes zoster and post-herpetic neuralgia in Italy: A retrospective, population-based study," *BMC Infectious Diseases,* vol. 10, no. 1, p. 230, 2010. |
| [14] | C. Alicino, C. Trucchi, C. Paganino, I. Barberis, S. Boccalini, D. Martinelli, B. Pellizzari, A. Bechini, A. Orsi, P. Bonanni, R. Prato, S. Iannazzo and G. Icardi, "Incidence of herpes zoster and post-herpetic neuralgia in Italy: Results from a 3-years population-based study," *Human Vaccines & Immunotherapeutics,* vol. 13, no. 2, pp. 399-404, 2017. |
| [15] | C. Trucchi, G. Gabutti, M. C. Rota and A. Bella, "Burden of varicella in Italy, 2001–2010: analysis of data from multiple sources and assessment of universal vaccination impact in three pilot regions," *Journal of Medical Microbiology,* vol. 64, no. 11, pp. 1387-94, 2015. |
| [16] | F. D'Ancona, C. D'Amario, F. Maraglino, G. Rezza and S. Iannazzo, "The law of compulsory vaccination in Italy: an update 2 years after the introduction," *Eurosurveillance,* vol. 24, no. 26, p. 1900371, 2019. |
| [17] | M. Marin and S. R. Bialek, Varicella/Herpes Zoster. In Control of Communicable Diseases Manual 16th edn, Washington, DC: American Public Health Association, 2015. |
| [18] | M. Brisson, W. J. Edmunds, N. J. Gay, B. Law and G. De Serres, "Modelling the impact of immunization on the epidemiology of varicella zoster virus," *Epidemiology & Infection,* vol. 125, no. 3, pp. 651-669, 2000. |
| [19] | H. H. Bernstein, E. P. Rothstein, B. M. Watson, K. S. Reisinger, M. M. Blatter, C. O. Wellman, S. A. Chartrand, I. Cho, A. Ngail and C. J. White, "Clinical survey of natural varicella compared with breakthrough varicella after immunization with live attenuated Oka/Merck varicella vaccine," *Pediatrics,* vol. 92, no. 6, pp. 833-837, 1993. |
| [20] | H. S. Izurieta, P. W. M. Strebel and P. A. Blake, "Postlicensure effectiveness of varicella vaccine during an outbreak in a child care center," *JAMA,* vol. 278, pp. 1495-99, 1997. |
| [21] | O. Sharomi, I. Xausa, R. Nachbar, M. Pillsbury, I. Matthews, T. Petigara, E. Elbasha and M. Pawaskar, "Modeling the Impact of Exogenous Boosting and Universal Varicella Vaccination on the Clinical and Economic Burden of Varicella and Herpes Zoster in a Dynamic Population for England and Wales," *Vaccines,* vol. 10, no. 9, p. 1416, 2022. |
| [22] | J. F. Seward, J. X. Zhang, T. J. Maupin, L. Mascola and A. O. Jumaan, "Contagiousness of varicella in vaccinated cases: a household contact study," *JAMA,* vol. 292, no. 6, pp. 704-8, 2004. |
| [23] | M. C. Schuette and H. W. Hethcote, "Modeling the effects of varicella vaccination programs on the incidence of chickenpox and shingles," *Bulletin of Mathematical Biology,* vol. 61, no. 6, pp. 1031-1064, 1999. |
| [24] | N. M. Ferguson, R. M. Anderson and G. P. Garnett, "Mass vaccination to control chickenpox: the influence of zoster," *Proceedings of the National Academy of Sciences,* vol. 93, no. 14, pp. 7231-35, 1996. |
| [25] | P. Poletti, A. Melegaro, M. Ajelli, E. Del Fava, G. Guzzetta and L. Faustini, "Perspectives on the impact of varicella immunization on herpes zoster. A model-based evaluation from three European countries," *PLOS ONE,* vol. 8, no. 4, p. e60732, 2013. |
| [26] | J. Troller, "Herpes-zoster in general practice," *Australian Family Physician,* vol. 16, pp. 1133-40, 1987. |
| [27] | H. Forbes, I. Douglas, A. Finn, J. Breuer, K. Bhaskaran, L. Smeeth, S. Packer, S. Langan, K. E. Mansfield, R. Marlow, H. Whitaker and C. Warren-Gash, "Risk of herpes zoster after exposure to varicella to explore the exogenous boosting hypothesis: self controlled case series study using UK electronic healthcare data," *BMJ,* vol. 368, 2020. |
| [28] | M. Riera-Montes, K. Bollaerts, U. Heininger, N. Hens, G. Gabutti, A. Gil, B. Nozad, G. Mirinaviciute, E. Flem, A. Souverain and T. Verstraeten, "Estimation of the burden of varicella in Europe before the introduction of universal childhood immunization," *BMC Infectious Diseases,* vol. 17, no. 1, p. 353, 2017. |
| [29] | M. Brisson and W. Edmunds, "Epidemiology of Varicella-Zoster Virus in England and Wales," *Journal of Medical Virology,* vol. 70, pp. S9-S14, 2003. |
| [30] | B. Kuter, H. Matthews, H. Shinefield, S. Black, P. Dennehy, B. Watson, K. Resinger, L. L. Kim, L. Lupinacci, J. Hartzel, I. Chan and Study Group for Varivax, "Ten year follow-up of healthy children who received one or two injections of varicella vaccine," *The Pediatric Infectious Disease Journal,* vol. 23, no. 2, pp. 132-137, 2004. |
| [31] | M. J. Ouwens, K. J. Littlewood, C. Sauboin, B. Téhard, F. Denis, P. Y. Boëlle and S. Alain, "The impact of 2-dose routine measles, mumps, rubella, and varicella vaccination in France on the epidemiology of varicella and zoster using a dynamic model with an empirical contact matrix," *Clinical Therapeutics,* vol. 37, no. 4, pp. 816-829, 2015. |
| [32] | A. van Hoek, A. Melegaro, E. Zagheni, J. Edmunds and N. Gay, "Modelling the impact of a combined varicella and zoster vaccination programme on the epidemiology of varicella zoster virus in England," *Vaccine,* vol. 29, no. 13, pp. 2411-2420, 2011. |
| [33] | M. Pillsbury, C. Carias, S. Samant, D. Greenberg and M. Pawaskar, "Comparison of performance of varicella vaccines via infectious disease modeling," *Vaccine,* vol. 40, pp. 3954-3962, 2022. |
| [34] | S. A. Kujawski, C. Burgess, O. Agi, Z. Attias-Geva, M. Pillsbury, D. Greenberg, G. Bencina and M. Pawaskar, "The health and economic impact of switching vaccines in universal varicella vaccination programs using a dynamic transmission model: An Israel case study," *Human Vaccines & Immunotherapeutics,* p. 2124784, 2022. |
| [35] | M. Brisson, W. J. Edmunds and N. J. Gay, "Varicella vaccination: impact of vaccine efficacy on the epidemiology of VZV," *Journal of Medical Virology,* vol. 70, no. S1, pp. S31-S37, 2003. |
| [36] | L. Scalone, P. A. Cortesi, R. Ciampichini, G. Cesana and L. G. Mantovani, "Health related quality of life norm data of the Italian general population: results using the EQ-5D-3L and EQ-5D-5L instruments.," *Epidemiology Biostatistics, and Public Health,* vol. 3, p. 12, 2015. |
| [37] | P. Wutzler, P. Bonanni, M. Burgess, M. Gershon, M. Sáfadi and G. Casabona, "Varicella vaccination - the global experience," *Expert Review of Vaccines,* vol. 16, no. 8, pp. 833-843, 2017. |
| [38] | Ministero Delle'Economia E Delle Finanze, "Gazetta Ufficiale Della Republica Italiana, Serie Generale No 386," Ministero Dell'Economia E Delle Finanze, Rome, 1974. |
| [39] | Ministero della Salute, "DPCM 12 gennaio 2017, Allegato 4," Ministero della Salute, Rome, 2017. |
| [40] | Farmadati Italia, "Farmadati Italia," 2022. [Online]. Available: https://www.farmadati.it/. [Accessed 26 10 2022]. |
| [41] | Agenzia Italiana del Farmaco, "Linea guida per la comilazione del Dossier a supporto della domanda di rimborsabilita e prezzo di un medicinale - Estrata sezione E," Agenzia Italiana del Farmaco, Roma, 2020. |
| [42] | E. Barbieri, G. Porcu, T. Petigara, F. Senese, G. M. Prandi, A. Scamarcia, L. Cantarutti, A. Cantarutti and C. Giaquinto, "The Economic Burden of Pneumococcal Disease in Children: A Population-Based Investigation in the Veneto Region of Italy," *Children.,* vol. 9, no. 9, p. 1347, 2022 Sep 3. |
| [43] | L. Coudeville, A. Brunot, C. Giaquinto, C. Lucioni and B. Dervaux, "Varicella vaccination in Italy : an economic evaluation of different scenarios," *Pharmacoeconomics,* vol. 22, no. 13, pp. 839-55, 2004. |
| [44] | P. Fornaro, F. Gandini, M. Marin, C. Pedrazzi, P. Piccoli, D. Tognetti, B. M. Assael, C. Lucioni and S. Mazzi, "Epidemiology and cost analysis of varicella in Italy: results of a sentinel study in the pediatric practice," *The Pediatric Infectious Disease Journal,* vol. 18, no. 5, 1999. |
| [45] | C. Giaquinto, M. Sturkenboom, S. Mannino, F. Arpinelli, A. Nicolosi and L. Cantarutti, "Epidemiology and outcomes of varicella in Italy: results of a prospective study of children (0-14 years old) followed up by pediatricians (Pedianet study)]," *Annali di igiene : medicina preventiva e di comunita,* vol. 14, no. 4 Suppl 6, 2002. |
| [46] | L. Coudeville, F. Paree, T. Lebrun and J. C. Sailly, "The value of varicella vaccination in healthy children: cost-benefit analysis of the situation in France," *Vaccine,* vol. 17, no. 2, pp. 142-51, 1999. |
| [47] | M Pillsbury, M Carias, C Samant, M Pawaskar, "PIN86 Modeling Performance Parametrization of Varicella Vaccines.," *Value in Health,* vol. 23, p. S559, 2020. |
