# Supplementary material for: The clinical and economic costs associated with regional disparities in varicella vaccine coverage in Italy over 50 years (2020–2070)
Source: Sci Rep. 2024 May 24;14:11929. doi: 10.1038/s41598-024-60649-8 (PMC11126631; doi:10.1038/s41598-024-60649-8)
Supplement: Supplementary file 2 — Supplementary Information 2. [file 41598_2024_60649_MOESM2_ESM.docx]

**The Clinical and Economic Costs Associated with Regional Disparities in Varicella Vaccine Coverage in Italy Over 50 Years (2020-2070)**

**Supplemental Results (S2)**

**Authors:** Lang, J.C., PhD^1*^, Samant, S., MPH^2^, Cook, J.R., PhD^3^, Ranjan, S., MSc ^3^, Senese, F., MPH^4^, Starnino, S., PhD^5^, Giuffrida, S., MD^6^, Azzari, C., MD^7^, Baldo, V., MD^8^, Pawaskar, M., PhD^2^

^1*^ Biostatistics and Research Decision Sciences, Merck Canada Inc., Kirkland, QC, Canada; [john.lang@merck.com](mailto:john.lang@merck.com)

^2^ Center for Observational and Real-World Evidence, Merck & Co., Inc., Rahway, NJ, USA

^3^ CHEORS, North Wales, PA, USA

^4^ Market Access, MSD Italy, Rome, Italy

^5^ Medical Affairs, MSD Italy, Rome, Italy

^6^ LHU Reggio Calabria, Calabria, Italy

^7^ Department of Health Sciences, University of Florence, and Meyer Children’s University Hospital, Florence, Italy

^8^ Department of Cardiac Thoracic Vascular Sciences, Hygiene and Public Health Unit, and Public Health University of Padua, Padua, Italy

**Contents**

[**S2.1 Calibration Results (validation)** 3](#_Toc161175712)

[**S2.2 Health and Economic Outcomes Results** 19](#_Toc161175713)

[**References** 32](#_Toc161175714)

**Tables**

[**Table S2.1: Calibrated parameters for VZV DTM (excluding demographic parameters)** 3](#_Toc161175715)

[**Table S2.2: Empirical and calibrated varicella seroprevalence data** 3](#_Toc161175716)

[**Table S2.3: Empirical and calibrated zoster incidence data** 5](#_Toc161175717)

[**Table S2.4 Varicella-associated hospitalization incidence** 5](#_Toc161175718)

[**Table S2.5: Calibrated parameter values for first- and second-dose VCRs** 11](#_Toc161175719)

[**Table S2.6: Costs, QALYs lost and net monetary benefit summary** 22](#_Toc161175720)

**Figures**

[**Figure S2.1: Varicella seroprevalence calibration results.** 6](#_Toc161175721)

[**Figure S2.2: HZ incidence calibration results.** 6](#_Toc161175722)

[**Figure S2.3: Probability of hospitalization per varicella infection by age.** 7](#_Toc161175723)

[**Figure S2.4: Varicella hospitalization incidence.** 7](#_Toc161175724)

[**Figure S2.5: Varicella incidence.** 8](#_Toc161175725)

[**Figure S2.6: Age stratified varicella-associated hospitalization incidence versus time.** 9](#_Toc161175726)

[**Figure S2.7: Aggregated varicella hospitalization incidence versus time.** 10](#_Toc161175727)

[**Figure S2.8: National and regional varicella VCRs.** 12](#_Toc161175728)

[**Figure S2.9: Calibrated VCR uptake parameter adoption_Year versus year of introduction of mandatory UVV.** 18](#_Toc161175729)

[**Figure S2.10: Varicella incidence under strategy B/D.** 19](#_Toc161175730)

[**Figure S2.11: Cumulative cost and NMB versus regional equilibrium first-dose VCR under strategy B.** 20](#_Toc161175731)

[**Figure S2.12: Deterministic and probabilistic sensitivity analyses.** 31](#_Toc161175732)

# **S2.1 Calibration Results (validation)**

**Table S2.1: Calibrated parameters for VZV DTM (excluding demographic parameters)**

| **Symbol** | **Description** | **Calibrated value** |
| --- | --- | --- |
| $rr\left[ 1 \right]$ | Relative risk for ages 0-3 | 4.3888 |
| $rr\left[ 2 \right]$ | Relative risk for ages 4-19 | 4.3510 |
| $rr\left[ 3 \right]$ | Relative risk for ages 20+ | 2.0466 |
| $\omega m$ | Natural maternal immunity waning rate | 2.8244 |
| $\omega$ | Reactivation rate parameter for “bathtub function” | 59.3584 |
| $\phi$ |  | 0.4740 |
| $\eta$ |  | 1.5982 |
| $\pi$ |  | 4.8121 |
| $h\left[ 0 \right]$ | Probability of hospitalization per varicella case for age 0 | 0.007590 |
| $h\left[ 1 \right]$ | Probability of hospitalization per varicella case for age 1 | 0.004057 |
| $h\left[ 5 \right]$ | Probability of hospitalization per varicella case for age 5 | 0.002931 |
| $h\left[ 25 \right]$ | Probability of hospitalization per varicella case for age 25 | 0.005738 |
| $h\left[ 100 \right]$ | Probability of hospitalization per varicella case for age 100 | 0.020888 |

**Table S2.2: Empirical and calibrated varicella seroprevalence data**

| **Empirical data** | | | **Model output - Italy** | |
| --- | --- | --- | --- | --- |
| **Mid-point of age group** | **Seroprevalence** | | **Mid-point of age group** | **Seroprevalence** |
| 0.5 | 0.389948 | | 0.041667 | 0.766641 |
| 1.5 | 0.116118 | | 0.291667 | 0.390408 |
| 3.5 | 0.331023 | | 0.708333 | 0.24646 |
| 7.5 | 0.670711 | | 0.958333 | 0.225184 |
| 12.5 | 0.847487 | | 1.041667 | 0.209936 |
| 17.5 | 0.856153 | | 1.125 | 0.199545 |
| 30 | 0.911612 | | 1.208333 | 0.193063 |
| 70 | 0.984402 | | 1.291667 | 0.18972 |
|  | | | 1.375 | 0.188897 |
|  |  |  | 1.458333 | 0.190089 |
|  |  |  | 1.541667 | 0.192892 |
|  |  |  | 1.625 | 0.196975 |
|  |  |  | 1.708333 | 0.202074 |
|  |  |  | 1.791667 | 0.207973 |
|  |  |  | 1.875 | 0.214498 |
|  |  |  | 1.958333 | 0.221509 |
|  |  |  | 2.5 | 0.314114 |
|  |  |  | 3.5 | 0.399827 |
|  |  |  | 4.5 | 0.475318 |
|  |  |  | 5.5 | 0.528654 |
|  |  |  | 6.5 | 0.576645 |
|  |  |  | 7.5 | 0.619771 |
|  |  |  | 8.5 | 0.658508 |
|  |  |  | 9.5 | 0.693301 |
|  |  |  | 10.5 | 0.727494 |
|  |  |  | 11.5 | 0.757875 |
|  |  |  | 12.5 | 0.784869 |
|  |  |  | 13.5 | 0.808853 |
|  |  |  | 14.5 | 0.830163 |
|  |  |  | 15.5 | 0.845447 |
|  |  |  | 16.5 | 0.859355 |
|  |  |  | 17.5 | 0.872011 |
|  |  |  | 18.5 | 0.883528 |
|  |  |  | 19.5 | 0.894008 |
|  |  |  | 22.5 | 0.91378 |
|  |  |  | 27.5 | 0.931176 |
|  |  |  | 32.5 | 0.94422 |
|  |  |  | 37.5 | 0.957046 |
|  |  |  | 42.5 | 0.965935 |
|  |  |  | 47.5 | 0.971593 |
|  |  |  | 52.5 | 0.976473 |
|  |  |  | 57.5 | 0.980597 |
|  |  |  | 62.5 | 0.982904 |
|  |  |  | 67.5 | 0.984666 |
|  |  |  | 72.5 | 0.985956 |
|  |  |  | 77.5 | 0.98687 |
|  |  |  | 82.5 | 0.987674 |
|  |  |  | 87.5 | 0.988358 |
|  |  |  | 95 | 0.989137 |
| Source | | [1, 2] | -- | Calculated |

**Table S2.3: Empirical and calibrated zoster incidence data**

| **Minimum age** | **Maximum age** | **Age** | **Zoster incidence per 1,000 person-years (data)** | **Zoster incidence per 1,000 person-years (model output Italy)** |
| --- | --- | --- | --- | --- |
| 15 | 20 | 17.5 | 1.71 | 1.062067 |
| 20 | 25 | 22.5 | 1.82 | 0.827633 |
| 25 | 30 | 27.5 | 1.91 | 1.243456 |
| 30 | 35 | 32.5 | 2.25 | 1.894122 |
| 35 | 40 | 37.5 | 1.95 | 2.59818 |
| 40 | 45 | 42.5 | 2.51 | 3.366055 |
| 45 | 50 | 47.5 | 3.06 | 4.191332 |
| 50 | 55 | 52.5 | 4 | 4.958025 |
| 55 | 60 | 57.5 | 5.6 | 5.628027 |
| 60 | 65 | 62.5 | 6.5 | 6.301467 |
| 65 | 70 | 67.5 | 6.1 | 6.857709 |
| 70 | 75 | 72.5 | 9.1 | 7.292325 |
| 75 | 80 | 77.5 | 8.2 | 7.605622 |
| 80 | 85 | 82.5 | 8.6 | 7.780241 |
| 85 | 100 | 92.5 | 5.6 | 7.882696 |
| Source | | | [3, 4] | Calculated |

**Table S2.4 Varicella-associated hospitalization incidence**

| **Mid-point of age group** | **Hospitalization incidence per 100,000**  **(data)** | **Hospitalization incidence per 100,000**  **(model output Italy)** |
| --- | --- | --- |
| 0.5 | 52.05 | 50.81589 |
| 3 | 34.45 | 33.58517 |
| 10 | 12.15 | 12.44761 |
| 20 | 2.85 | 3.980615 |
| 35 | 2.4 | 1.907336 |
| 55 | 0.6 | 0.955075 |
| 70 | 0.5 | 0.466427 |
| 87.5 | 0.45 | 0.325868 |
| Source | [5] | Calculated |

**Figure S2.1: Varicella seroprevalence calibration results.**

**
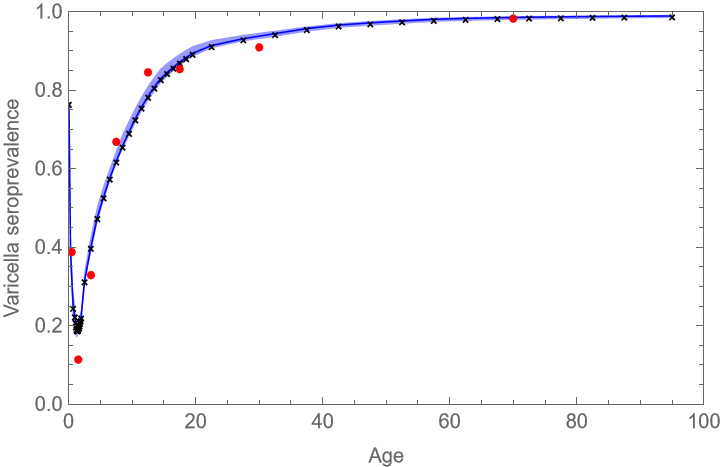
**

**Note:** (Dots) Varicella seroprevalence data. [1, 2] (Line) Calibrated model output from national-level model. (Exes) Calibrated model output aggregated from regional-level models. (Shaded area) 95% region computed from model simulations with PSA parameter realizations.

**Figure S2.2: HZ incidence calibration results.**


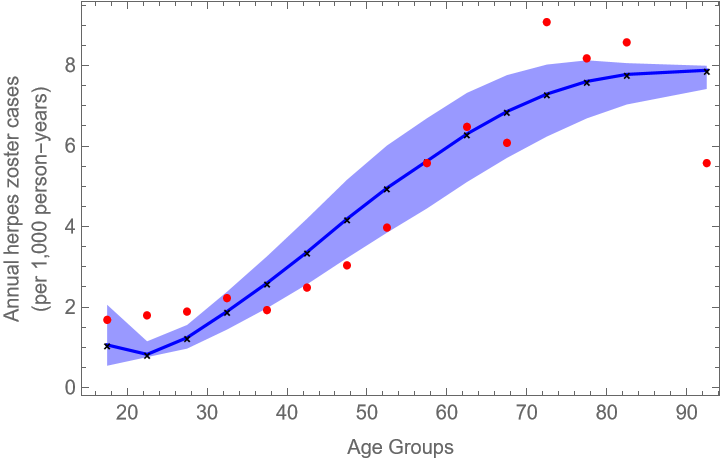


**Note**: (Dots) HZ incidence data. [6] (Line) Calibrated model output from national-level model. (Exes) Calibrated model output aggregated from regional-level models. (Shaded area) 95% region computed from model simulations with PSA parameter realizations.

**Figure S2.3: Probability of hospitalization per varicella infection by age.**


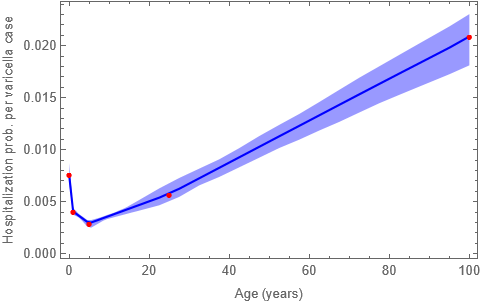


**Note:** (Dots) Calibrated parameter values. (Line) Linear interpolation between calibrated parameter values. (Shaded area) 95% region computed from model simulations with PSA parameter realizations.

**Figure S2.4: Varicella hospitalization incidence.**


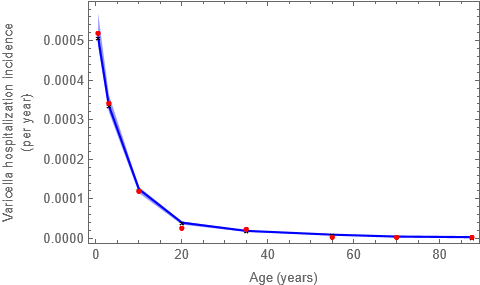


**Note:** (Dots). Hospitalization incidence data. [7] (Line) Calibrated model output from national-level model. (Exes) Calibrated model output aggregated form regional-level model. (Shaded area) 95% region computed from model simulations with PSA parameter realizations.

**Figure S2.5: Varicella incidence.**

**
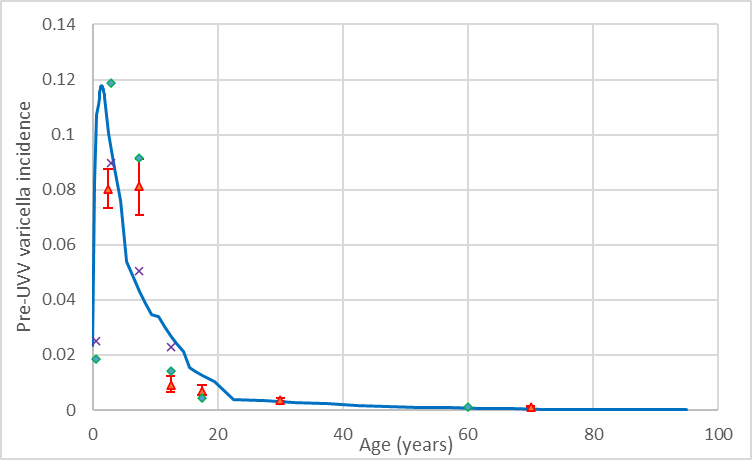
**

**Note:** (Line) model output. (Red triangles; bars indicate 95% CI) Riera-Montes, et al. (2017) [8], (green triangles) Baldo, et al. (2009) [9], (purple exes), Atti, et al. (2002) [10].

**Figure S2.6: Age** **stratified varicella-associated hospitalization incidence versus time.**


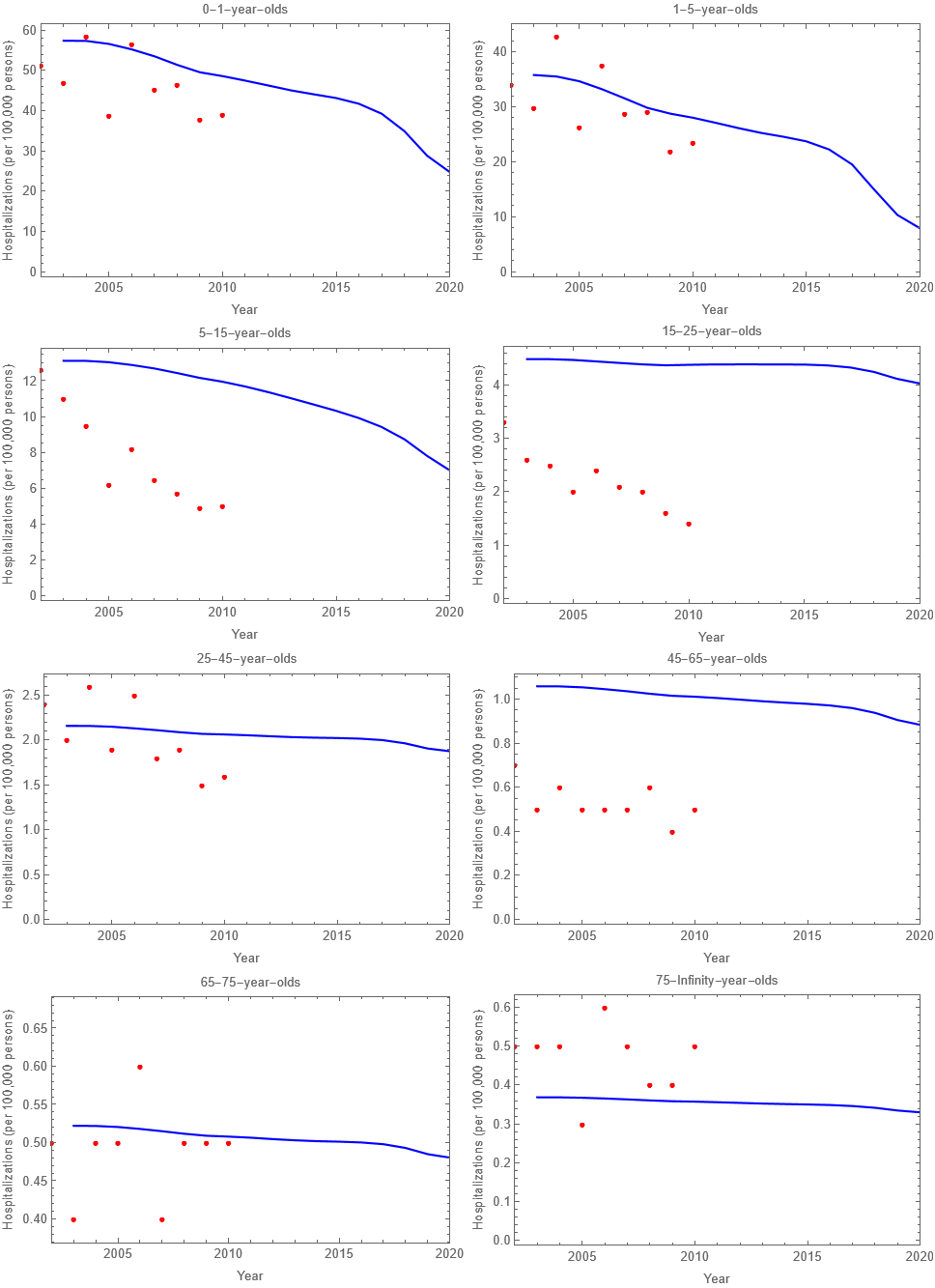


**Note:** (Dots) Incidence from Trucchi et al [5]. (Line) Incidence estimated from calibrated model*.*

**Figure S2.7: Aggregated varicella hospitalization incidence versus time.**


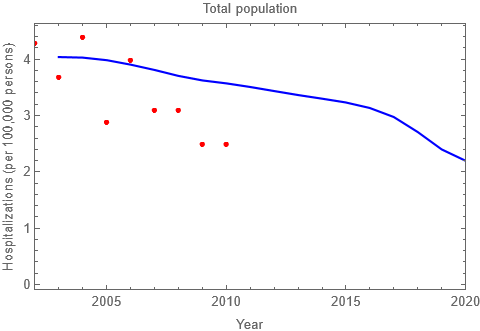


**Note:** (Dots) Hospitalization incidence data from Trucchi et al [5]. (Line) Hospitalization incidence estimated from calibrated model.

**Table S2.5: Calibrated parameter values for first- and second-dose VCRs**

| **Region** | $\boldsymbol{vc}\boldsymbol{r}_{\boldsymbol{initial}}^{\boldsymbol{1}}$ | $\boldsymbol{vc}\boldsymbol{r}_{\boldsymbol{equilibrium}}^{\boldsymbol{1}}$ | $\boldsymbol{adoptio}\boldsymbol{n}_{\boldsymbol{year}}^{\boldsymbol{1}}$ | $\boldsymbol{adoptio}\boldsymbol{n}_{\boldsymbol{speed}}^{\boldsymbol{1}}$ | $\boldsymbol{vc}\boldsymbol{r}_{\boldsymbol{equilibrium}}^{\boldsymbol{2}}$ |
| --- | --- | --- | --- | --- | --- |
| Abruzzo | 0.050 | 0.937 | 2016.0 | 1.350 | 1.000 |
| Basilicata | 0.050 | 0.972 | 2009.3 | 0.001 | 0.949 |
| Calabria | 0.050 | 0.903 | 2010.6 | 0.002 | 0.899 |
| Campania | 0.000 | 0.899 | 2015.5 | 1.527 | 0.929 |
| Emilia Romagna | 0.020 | 0.956 | 2015.8 | 0.933 | 0.993 |
| Friuli Venezia Giulia | 0.000 | 0.941 | 2012.6 | 1.864 | 0.992 |
| Lazio | 0.050 | 0.957 | 2016.1 | 1.267 | 0.938 |
| Liguria | 0.050 | 0.947 | 2015.4 | 1.809 | 0.936 |
| Lombardia | 0.004 | 0.959 | 2016.5 | 0.003 | 0.996 |
| Marche | 0.000 | 0.940 | 2015.4 | 1.505 | 0.991 |
| Molise | 0.000 | 0.929 | 2012.0 | 2.035 | 0.913 |
| Piemonte | 0.018 | 0.965 | 2017.2 | 0.247 | 0.994 |
| Prov. Auton. Bolzano | 0.016 | 0.866 | 2016.0 | 1.804 | 0.901 |
| Prov. Auton. Trento | 0.000 | 0.956 | 2015.4 | 0.002 | 0.984 |
| Puglia | 0.050 | 0.963 | 2004.2 | 2.618 | 0.926 |
| Sardegna | 0.000 | 0.953 | 2011.5 | 0.940 | 0.976 |
| Sicilia | 0.050 | 0.959 | 2006.1 | 0.009 | 0.773 |
| Toscana | 0.050 | 0.952 | 2008.9 | 0.002 | 0.964 |
| Umbria | 0.009 | 0.969 | 2016.2 | 0.002 | 1.000 |
| Valle d'Aosta | 0.009 | 0.949 | 2017.5 | 0.467 | 0.984 |
| Veneto | 0.050 | 0.950 | 2004.8 | 0.002 | 0.978 |

**Figure S2.8: National and regional varicella VCRs.**


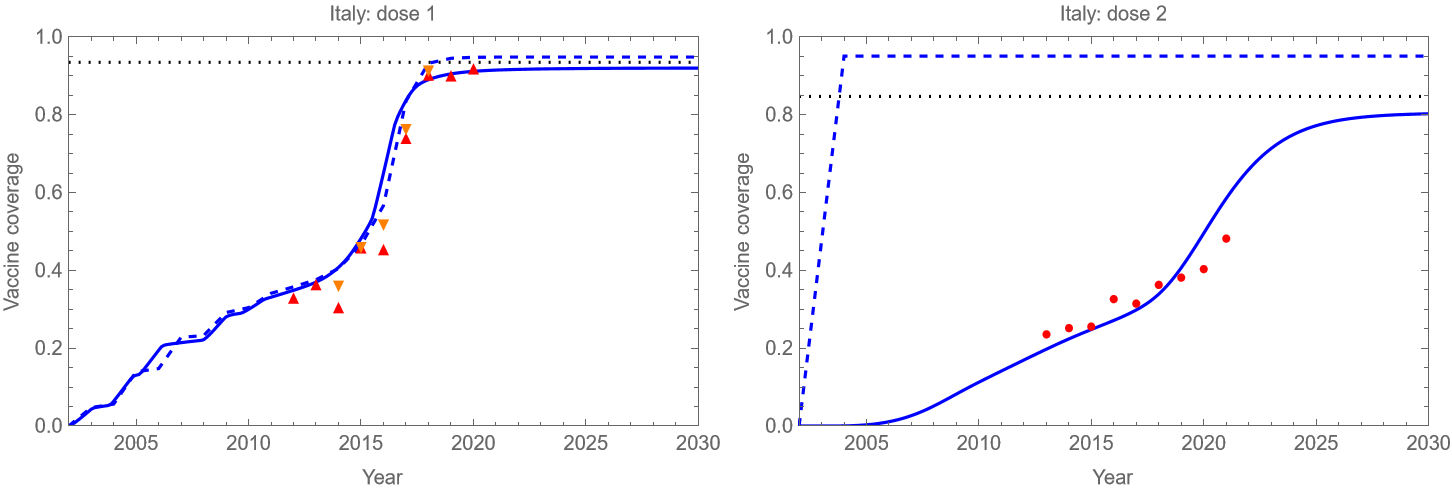


**Note:** (Dotted black line) Average MMR VCR. (Red up triangles) Varicella first-dose VCR at 2 years (shifted one year to the left). (Orange down triangles) Varicella first-dose VCR at 4 years (shifted three years to the left). (Red circles) Varicella second-dose VCR at 5 years. (Solid blue line) Calibrated model estimates of VCR for 1-year-olds (left) and 5-year-olds (right). (Dashed blue line) Calibrated model vaccine uptake for 1-year-olds (left) and 5-year-olds (right).

**Figure S2.8 (continued).**

**
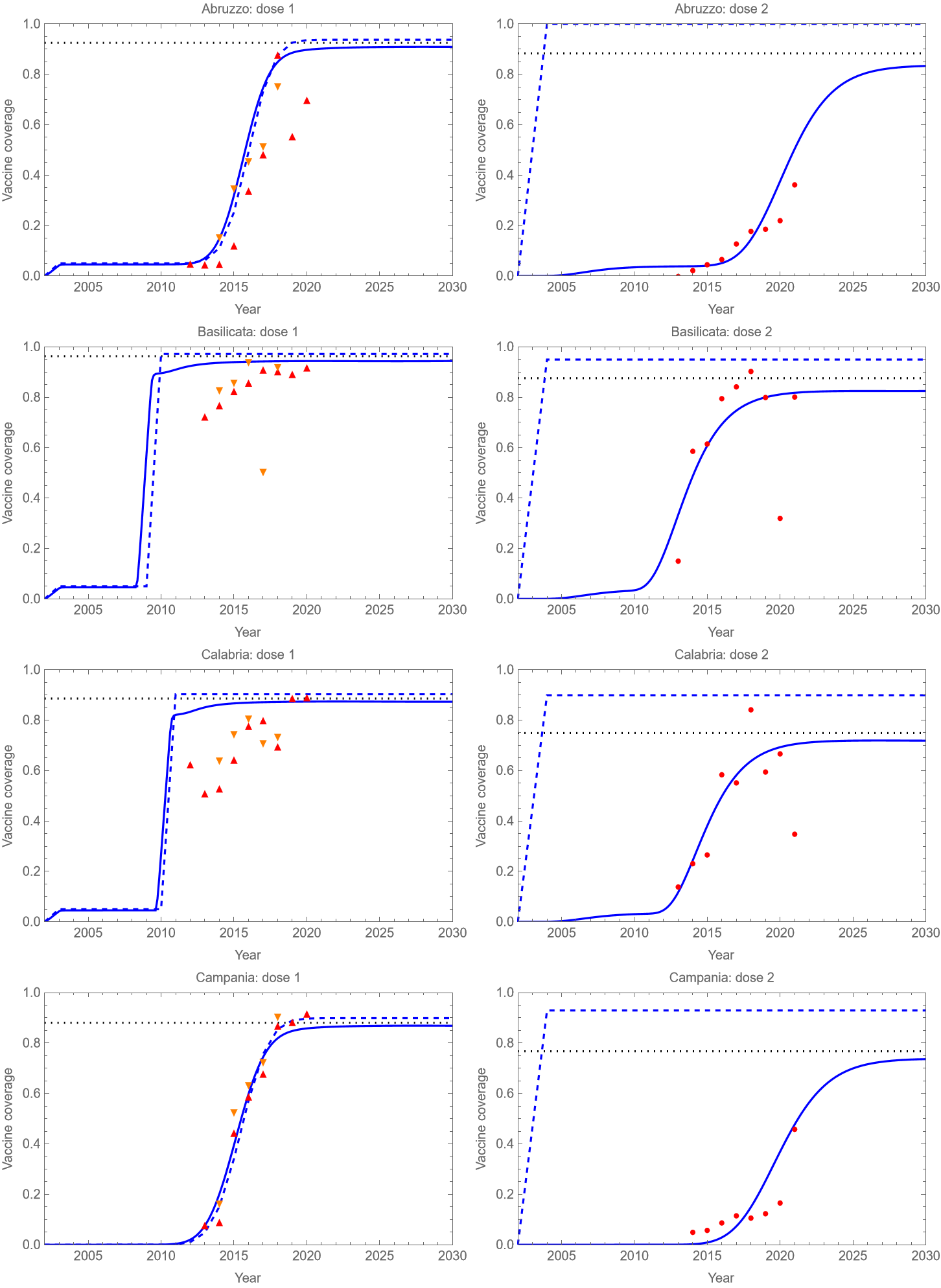
**

**Figure S2.8 (continued).**

**
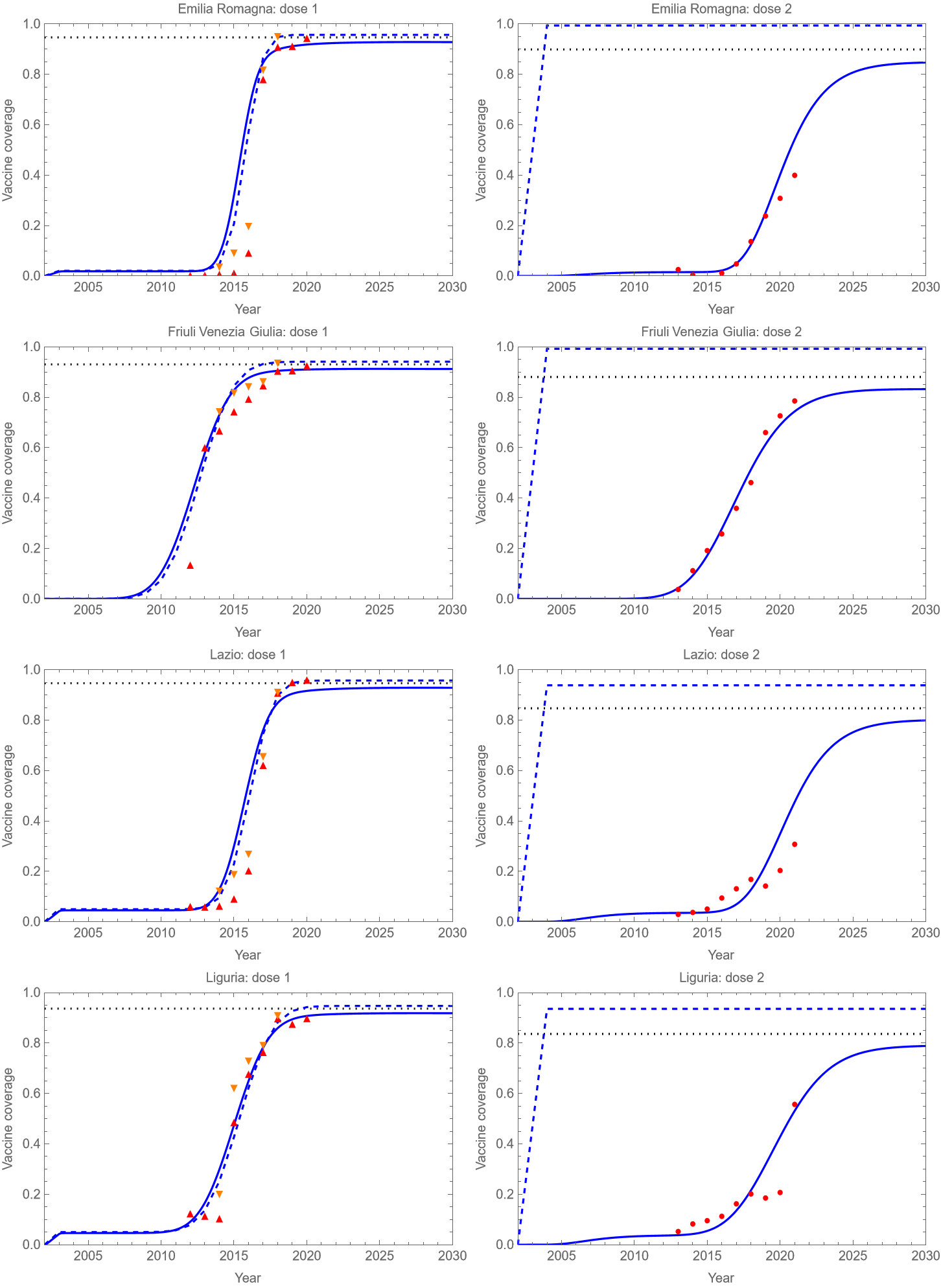
**

**Figure S2.8 (continued).**

**
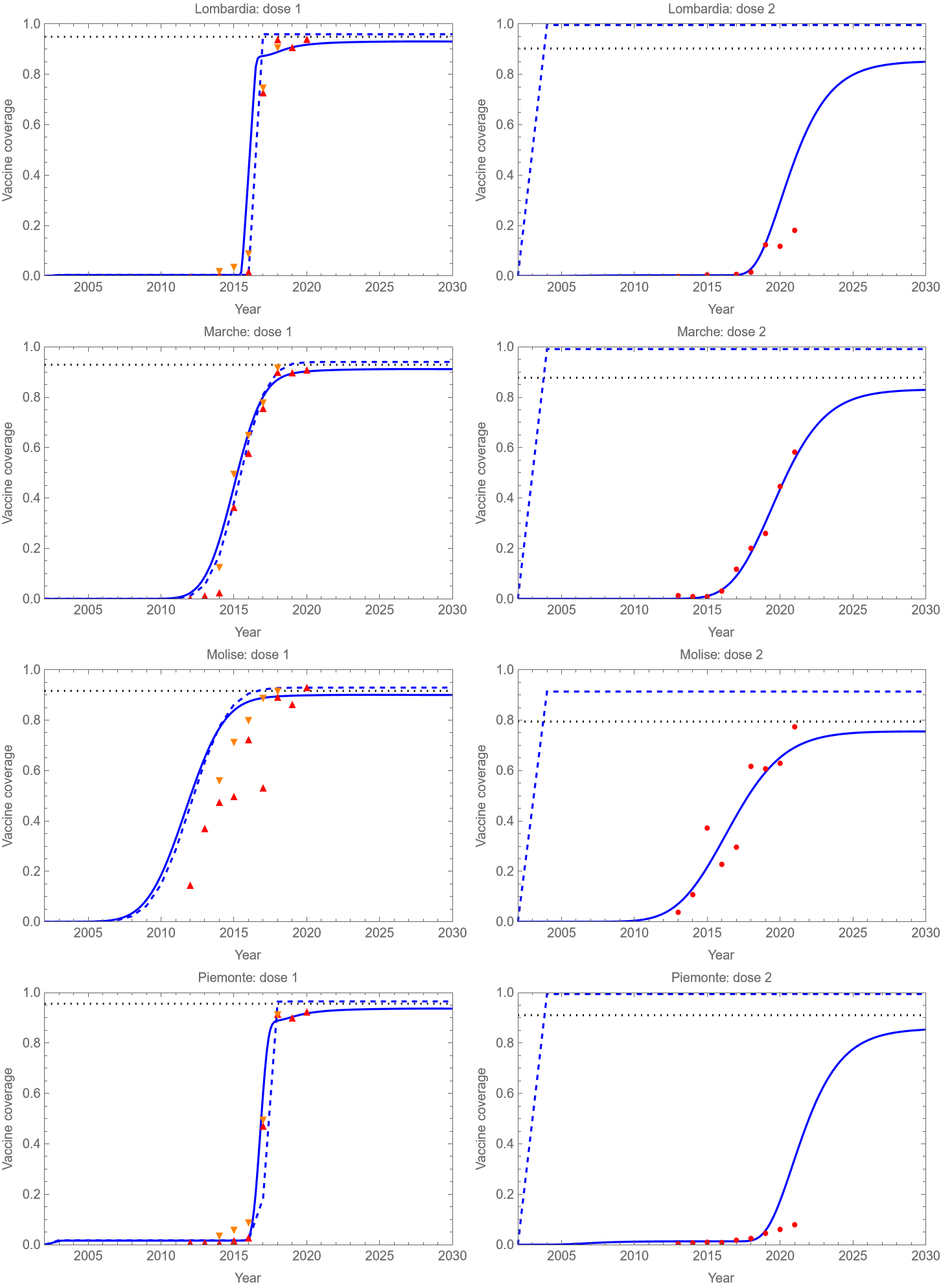
**

**Figure S2.8 (continued).**

**
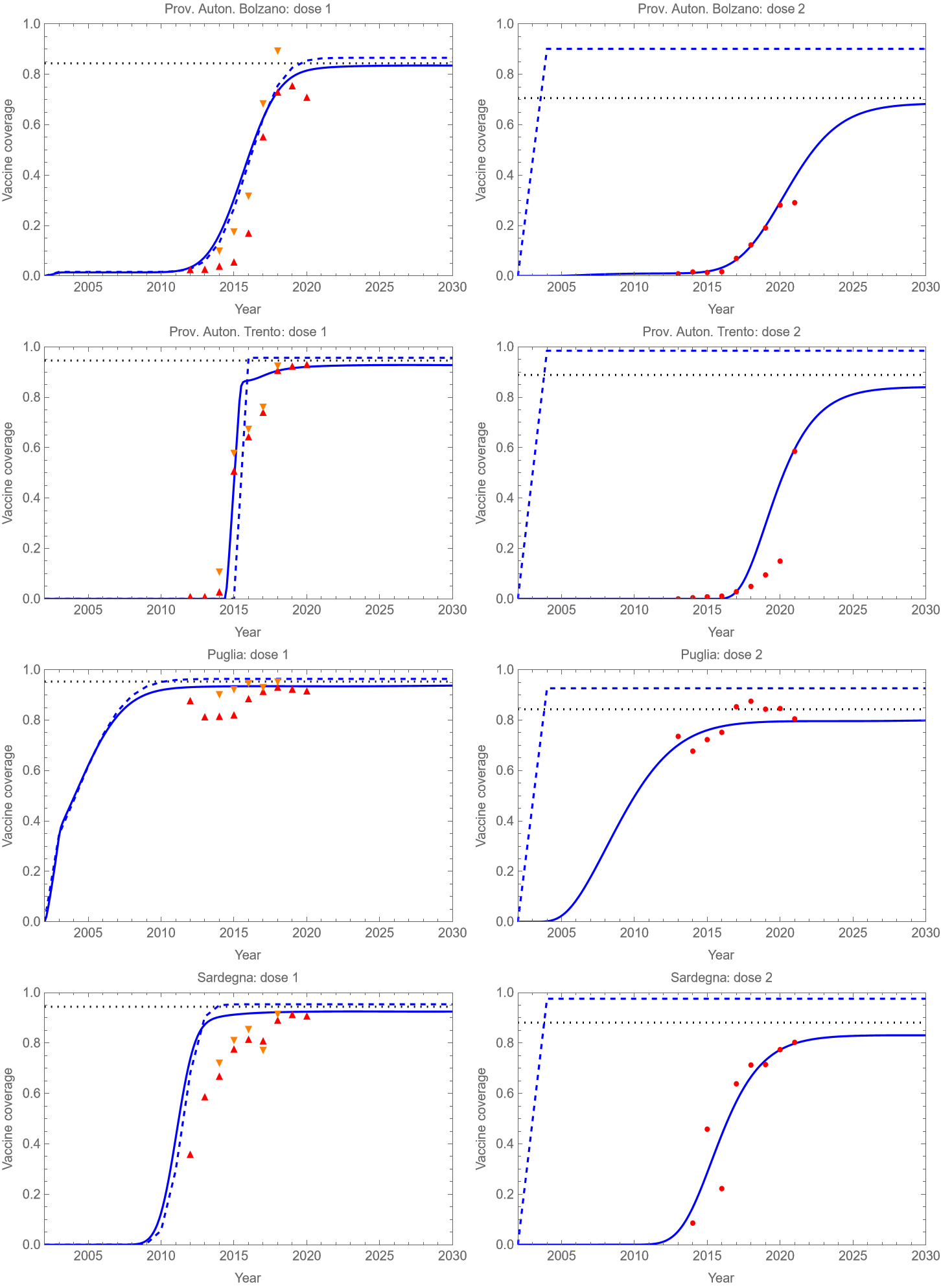
**

**Figure S2.8 (continued).**

**
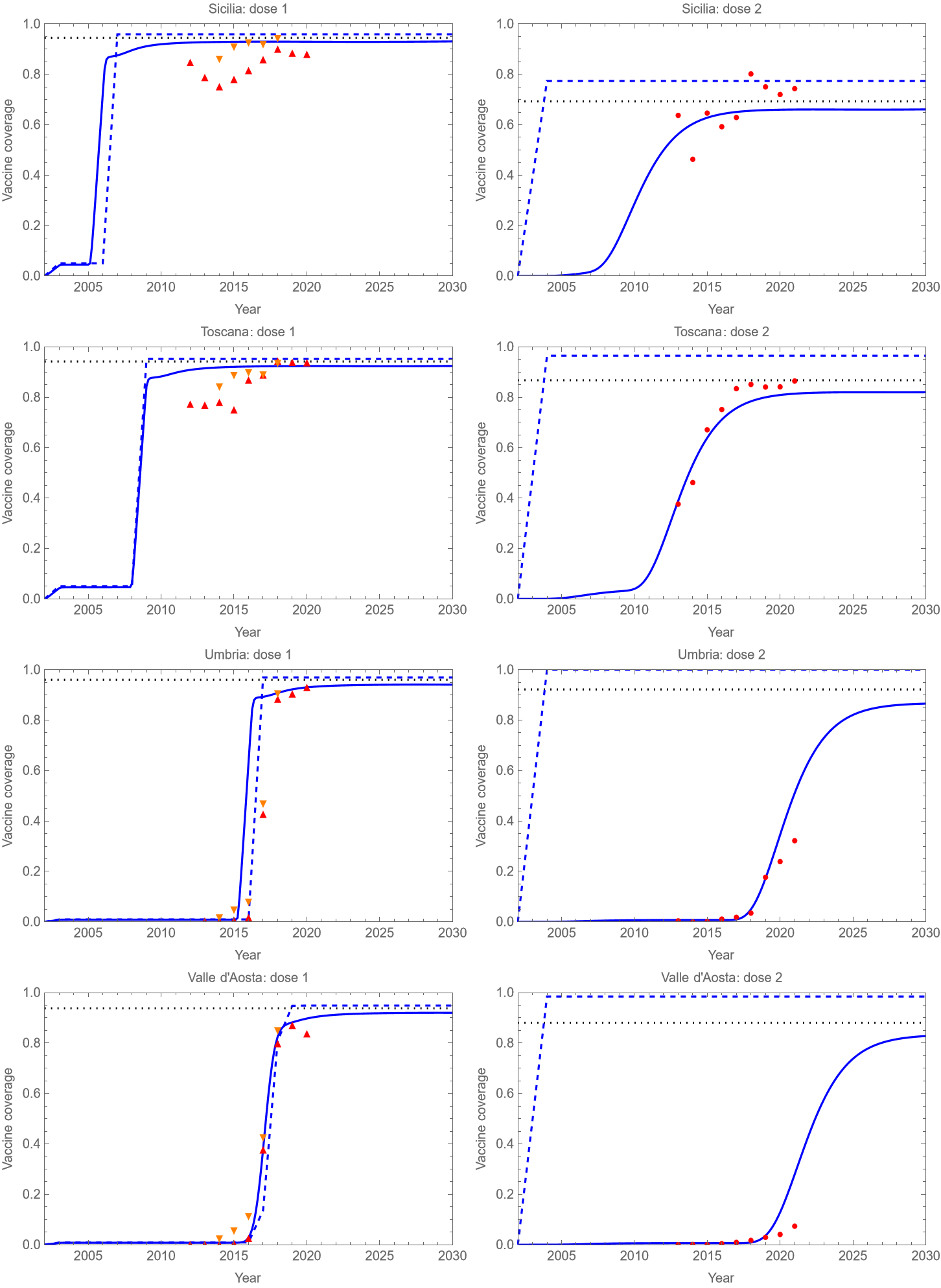
**

**Figure S2.8 (continued).**

**
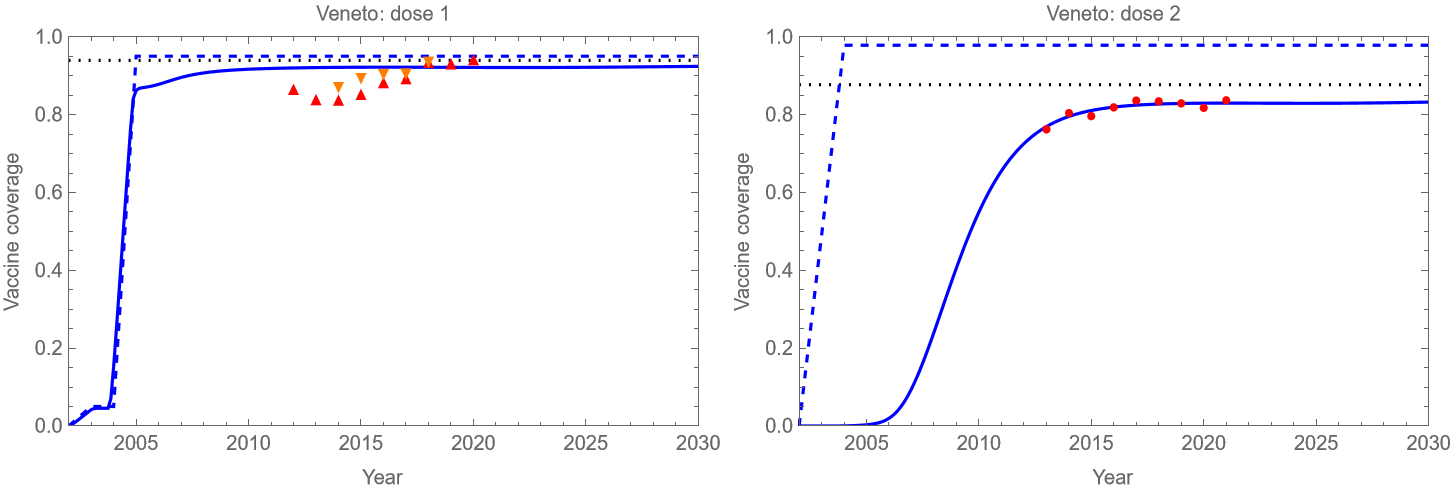
**

**Figure S2.9: Calibrated VCR uptake parameter adoption_Year versus year of introduction of mandatory UVV.**

**
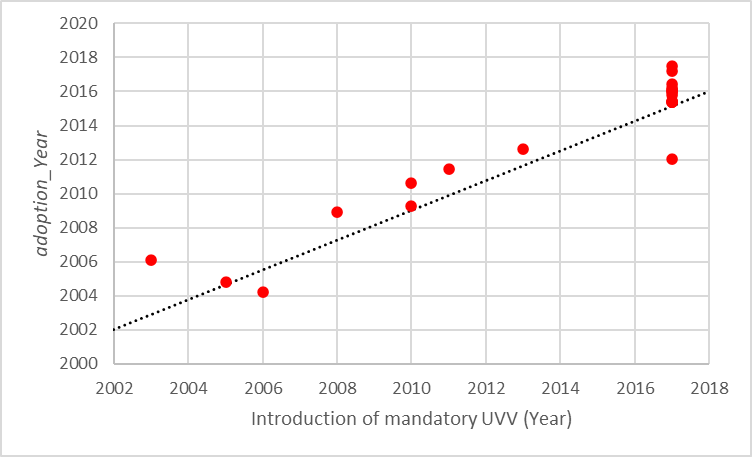
**

**Note:** Dotted line indicates the 45^o^ line whose intercept is the origin.

# **S2.2 Health and Economic Outcomes Results**

**Figure S2.10: Varicella incidence under strategy B/D.**

**
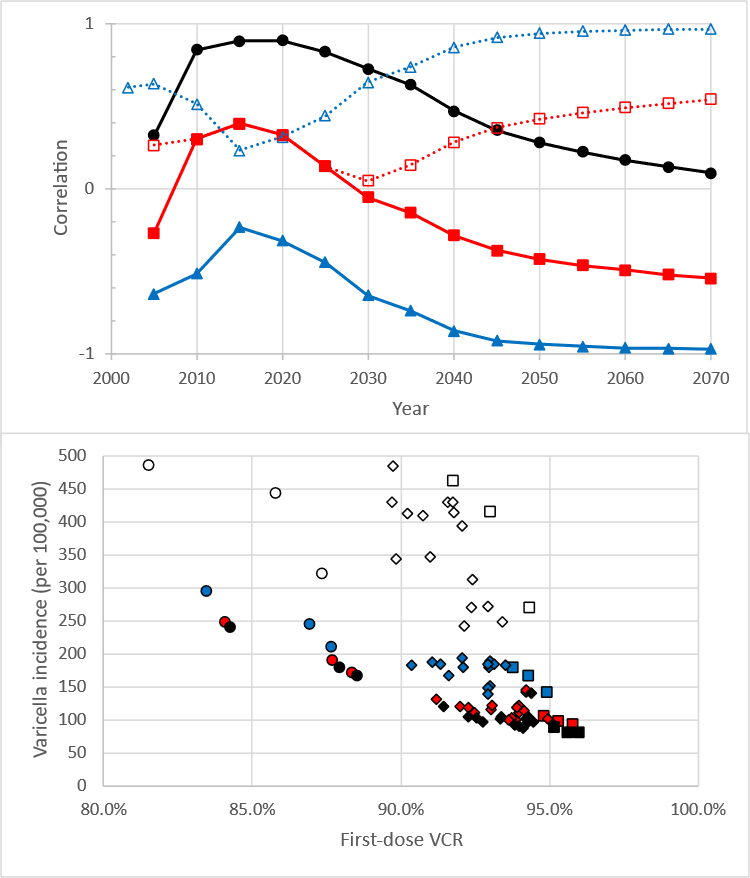
**

**Note 1:** Top row: (Solid lines, filled markers) Correlation. (Dotted lines, open markers) Magnitude of correlation. Correlation between varicella incidence under strategies A/C and (circles) year of adoption of mandatory UVV, (triangles) equilibrium first-dose VCR parameter (*vcr_equilibrium_1*), and (squares) equilibrium second-dose VCR parameter (*vcr_equilibrium_2*).

**Note 2:** Bottom row: Varicella incidence versus time-varying first-dose VCR in (open markers) 2020, (blue-filled markers) 2040, (red-filled markers) 2060, (black) 2070 for strategies A/C. Three regions with lowest and highest first-dose equilibrium VCR (*vcr_equilibrium_1*) are shown as circles and squares, respectively. Other regions are shown as diamonds.

**Figure S2.11: Cumulative cost and NMB versus regional equilibrium first-dose VCR under strategy B.**

**
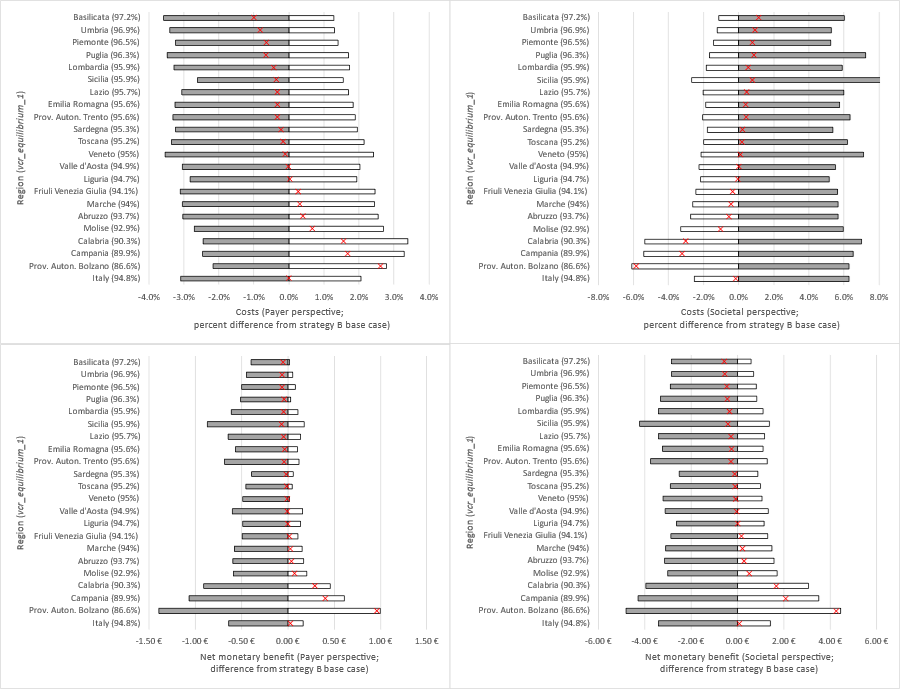
**

**Note 1:** (Top) Payer (left) and societal (right) costs. (Gray bars) Percent change in cost when vcr_equilibrium_1 is decreased by 10%. (White bars) Percent change in cost when vcr_equilibrium_1 is increased by 10%. (Red exes) Percent change in cost when vcr_equilibrium_1 is equal to the national average.

**Note 2:** (Bottom) Per capita payer (left) and societal (right) NMB with respect to strategy A with base case *vcr_equilibrium_1*. (Gray bars) NMB when vcr_equilibrium_1 is decreased by 10%. (White bars) NMB when *vcr_equilibrium_1* is increased by 10%. (Red exes) NMB when *vcr_equilibrium_1* is uniformly equal to the national average.

In all regions we found that the quadrivalent/quadrivalent strategy dominated the monovalent/quadrivalent strategy of the same manufacturer, i.e., strategy A dominated strategy C and strategy B dominated strategy D (see Table S2.6). This result followed from two main factors. First, clinical outcomes were the same for strategies of the same manufacturer, since the vaccine properties were assumed to be the same for monovalent and quadrivalent formulations by the same manufacturer. Second, total vaccination costs (i.e., vaccine cost, administration fee, and cost of adverse events) were higher for monovalent/quadrivalent strategies (C and D) than for quadrivalent/quadrivalent strategies (A and B) of the same manufacturer, driven by the lack of additional administration fee for quadrivalent vaccines (see Table 1 of the main text). Furthermore, in all regions we found that strategy A dominated strategy B. This result also followed from two main factors. First, whereas both MSD and GSK vaccines have similar 2-dose efficacies, MSD vaccines’ higher first-dose efficacy resulted in fewer breakthrough varicella infections under strategy A than under strategy B. Second, total vaccination costs were higher for strategy B than for strategy A (see Table 1 of the main text).

The DSA and PSA were conducted with respect to the net monetary benefit (NMB) of strategies A and B, using strategy A as the reference strategy. Based on the DSA, the most influential parameters with respect to the NMB from the payer perspective were vaccination cost, direct treatment cost, and QALYs lost due to natural varicella infection (see Figure S2.12 [top row]). Additionally, it was observed that HZ parameters (i.e., relative infectiousness of HZ reactivation [ρz] and waning rate for HZ reactivation [ηn]) were also influential with respect to payer NMB. Similarly, the most influential parameters with respect to the NMB from the societal perspective were indirect treatment cost, vaccination cost, long-term first-dose vaccine uptake. DSA results were consistent across all regions. Under the payer and societal perspectives, 82.5% (regionally: [77.9%, 89.1%]) and 92.8% (regionally: [88.6%, 93.9%]) of the 1,000 PSA model realizations simulated resulted in a negative NMB for Strategy B, i.e., fell above the willingness-to-pay threshold line in the cost-effectiveness plane (see Figure S2.12 [bottom row]).

**Table S2.6: Costs, QALYs lost and net monetary benefit summary**

|  | **Strategy A**  **(MMRV-MSD/**  **MMRV-MSD)** | **Strategy B**  **(MMRV-GSK/**  **MMRV-GSK)** | **Strategy C**  **(V-MSD/**  **MMRV-MSD)** | **Strategy D**  **(V-GSK/**  **MMRV-GSK)** |
| --- | --- | --- | --- | --- |
| **Italy** | | | | |
| **Payer costs (€)** | | | | |
| Millions | 950.54 | 1004.66 | 1015.70 | 1060.39 |
| Per capita | 16.04 | 16.96 | 17.14 | 17.90 |
| **Societal costs (€)** | | | | |
| Millions | 1969.72 | 2096.06 | 2034.88 | 2151.80 |
| Per capita | 33.25 | 35.38 | 34.35 | 36.32 |
| **QALYs lost** | | | | |
| Total | 17893.95 | 18633.09 | 17893.95 | 18633.09 |
| Per 100,000 | 30.20 | 31.45 | 30.20 | 31.45 |
| **Net monetary benefit (Payer costs; 1xGDP) (€)** | | | | |
| Millions | * | -73.85 | -65.17 | -129.59 |
| Per capita | * | -1.25 | -1.10 | -2.19 |
| **Net monetary benefit (Societal costs; 1xGDP) (€)** | | | | |
| Millions | * | -146.08 | -65.17 | -201.82 |
| Per capita | * | -2.47 | -1.10 | -3.41 |
| **Abruzzo** | | | | |
| **Payer costs (€)** | | | | |
| Millions | 19.97 | 21.02 | 21.26 | 22.12 |
| Per capita | 15.59 | 16.40 | 16.60 | 17.27 |
| **Societal costs (€)** | | | | |
| Millions | 44.11 | 46.39 | 45.39 | 47.49 |
| Per capita | 34.43 | 36.21 | 35.44 | 37.07 |
| **QALYs lost** | | | | |
| Total | 426.54 | 440.29 | 426.54 | 440.29 |
| Per 100,000 | 33.29 | 34.37 | 33.29 | 34.37 |
| **Net monetary benefit (Payer costs; 1xGDP) (€)** | | | | |
| Millions | * | -1.41 | -1.29 | -2.52 |
| Per capita | * | -1.10 | -1.01 | -1.97 |
| **Net monetary benefit (Societal costs; 1xGDP) (€)** | | | | |
| Millions | * | -2.66 | -1.29 | -3.76 |
| Per capita | * | -2.07 | -1.01 | -2.93 |
| **Basilicata** | | | | |
| **Payer costs (€)** | | | | |
| Millions | 8.06 | 8.51 | 8.62 | 9.00 |
| Per capita | 14.79 | 15.62 | 15.82 | 16.51 |
| **Societal costs (€)** | | | | |
| Millions | 16 | 16.98 | 16.57 | 17.46 |
| Per capita | 29.36 | 31.16 | 30.39 | 32.04 |
| **QALYs lost** | | | | |
| Total | 140.28 | 145.55 | 140.28 | 145.55 |
| Per 100,000 | 25.73 | 26.70 | 25.73 | 26.70 |
| **Net monetary benefit (Payer costs; 1xGDP) (€)** | | | | |
| Millions | * | -0.6 | -0.57 | -1.08 |
| Per capita | * | -1.09 | -1.04 | -1.98 |
| **Net monetary benefit (Societal costs; 1xGDP) (€)** | | | | |
| Millions | * | -1.13 | -0.57 | -1.61 |
| Per capita | * | -2.06 | -1.04 | -2.94 |
| **Calabria** | | | | |
| **Payer costs (€)** | | | | |
| Millions | 30.40 | 32.27 | 32.49 | 34.07 |
| Per capita | 16.33 | 17.34 | 17.46 | 18.31 |
| **Societal costs (€)** | | | | |
| Millions | 64.29 | 69.16 | 66.39 | 70.95 |
| Per capita | 34.55 | 37.17 | 35.68 | 38.13 |
| **QALYs lost** | | | | |
| Total | 566.65 | 596.51 | 566.65 | 596.51 |
| Per 100,000 | 30.45 | 32.06 | 30.45 | 32.06 |
| **Net monetary benefit (Payer costs; 1xGDP) (€)** | | | | |
| Millions | * | -2.68 | -2.10 | -4.47 |
| Per capita | * | -1.44 | -1.13 | -2.40 |
| **Net monetary benefit (Societal costs; 1xGDP) (€)** | | | | |
| Millions | * | -5.67 | -2.10 | -7.46 |
| Per capita | * | -3.04 | -1.13 | -4.01 |
| **Campania** | | | | |
| **Payer costs (€)** | | | | |
| Millions | 103.54 | 109.77 | 110.59 | 115.79 |
| Per capita | 18.41 | 19.51 | 19.66 | 20.58 |
| **Societal costs (€)** | | | | |
| Millions | 218.45 | 234.36 | 225.49 | 240.37 |
| Per capita | 38.84 | 41.66 | 40.09 | 42.73 |
| **QALYs lost** | | | | |
| Total | 1978.27 | 2083.46 | 1978.27 | 2083.46 |
| Per 100,000 | 35.17 | 37.04 | 35.17 | 37.04 |
| **Net monetary benefit (Payer costs; 1xGDP) (€)** | | | | |
| Millions | * | -9.04 | -7.05 | -15.06 |
| Per capita | * | -1.61 | -1.25 | -2.68 |
| **Net monetary benefit (Societal costs; 1xGDP) (€)** | | | | |
| Millions | * | -18.72 | -7.05 | -24.74 |
| Per capita | * | -3.33 | -1.25 | -4.40 |
| **Emilia Romagna** | | | | |
| **Payer costs (€)** | | | | |
| Millions | 72.29 | 76.14 | 77.09 | 80.25 |
| Per capita | 16.28 | 17.15 | 17.36 | 18.08 |
| **Societal costs (€)** | | | | |
| Millions | 152.73 | 161.10 | 157.54 | 165.21 |
| Per capita | 34.40 | 36.29 | 35.49 | 37.21 |
| **QALYs lost** | | | | |
| Total | 1421.74 | 1471.09 | 1421.74 | 1471.09 |
| Per 100,000 | 32.02 | 33.14 | 32.02 | 33.14 |
| **Net monetary benefit (Payer costs; 1xGDP) (€)** | | | | |
| Millions | * | -5.18 | -4.81 | -9.29 |
| Per capita | * | -1.17 | -1.08 | -2.09 |
| **Net monetary benefit (Societal costs; 1xGDP) (€)** | | | | |
| Millions | * | -9.69 | -4.81 | -13.8 |
| Per capita | * | -2.18 | -1.08 | -3.11 |
| **Friuli Venezia Giulia** | | | | |
| **Payer costs (€)** | | | | |
| Millions | 17.34 | 18.25 | 18.46 | 19.21 |
| Per capita | 14.43 | 15.19 | 15.36 | 15.98 |
| **Societal costs (€)** | | | | |
| Millions | 38.19 | 40.13 | 39.31 | 41.09 |
| Per capita | 31.78 | 33.4 | 32.72 | 34.19 |
| **QALYs lost** | | | | |
| Total | 364.99 | 376.18 | 364.99 | 376.18 |
| Per 100,000 | 30.37 | 31.30 | 30.37 | 31.30 |
| **Net monetary benefit (Payer costs; 1xGDP) (€)** | | | | |
| Millions | * | -1.21 | -1.13 | -2.17 |
| Per capita | * | -1.00 | -0.93 | -1.80 |
| **Net monetary benefit (Societal costs; 1xGDP) (€)** | | | | |
| Millions | * | -2.24 | -1.13 | -3.20 |
| Per capita | * | -1.86 | -0.93 | -2.66 |
| **Lazio** | | | | |
| **Payer costs (€)** | | | | |
| Millions | 93.51 | 98.81 | 99.88 | 104.25 |
| Per capita | 16.31 | 17.24 | 17.43 | 18.19 |
| **Societal costs (€)** | | | | |
| Millions | 199.38 | 212.04 | 205.75 | 217.49 |
| Per capita | 34.79 | 37.00 | 35.90 | 37.95 |
| **QALYs lost** | | | | |
| Total | 1860.07 | 1933.54 | 1860.07 | 1933.54 |
| Per 100,000 | 32.45 | 33.74 | 32.45 | 33.74 |
| **Net monetary benefit (Payer costs; 1xGDP) (€)** | | | | |
| Millions | * | -7.26 | -6.37 | -12.71 |
| Per capita | * | -1.27 | -1.11 | -2.20 |
| **Net monetary benefit (Societal costs; 1xGDP) (€)** | | | | |
| Millions | * | -14.63 | -6.37 | -20.07 |
| Per capita | * | -2.55 | -1.11 | -3.50 |
| **Liguria** | | | | |
| **Payer costs (€)** | | | | |
| Millions | 20.16 | 21.23 | 21.43 | 22.31 |
| Per capita | 13.27 | 13.98 | 14.11 | 14.69 |
| **Societal costs (€)** | | | | |
| Millions | 48.43 | 51.01 | 49.7 | 52.10 |
| Per capita | 31.89 | 33.59 | 32.73 | 34.31 |
| **QALYs lost** | | | | |
| Total | 497.39 | 511.76 | 497.39 | 511.76 |
| Per 100,000 | 32.75 | 33.70 | 32.75 | 33.70 |
| **Net monetary benefit (Payer costs; 1xGDP) (€)** | | | | |
| Millions | * | -1.46 | -1.28 | -2.55 |
| Per capita | * | -0.96 | -0.84 | -1.67 |
| **Net monetary benefit (Societal costs; 1xGDP) (€)** | | | | |
| Millions | * | -2.96 | -1.28 | -4.05 |
| Per capita | * | -1.95 | -0.84 | -2.66 |
| **Lombardia** | | | | |
| **Payer costs (€)** | | | | |
| Millions | 169.16 | 178.28 | 180.54 | 188.02 |
| Per capita | 16.94 | 17.86 | 18.08 | 18.83 |
| **Societal costs (€)** | | | | |
| Millions | 350.95 | 370.92 | 362.34 | 380.66 |
| Per capita | 35.16 | 37.16 | 36.30 | 38.13 |
| **QALYs lost** | | | | |
| Total | 3215.23 | 3334.89 | 3215.23 | 3334.89 |
| Per 100,000 | 32.21 | 33.41 | 32.21 | 33.41 |
| **Net monetary benefit (Payer costs; 1xGDP) (€)** | | | | |
| Millions | * | -12.32 | -11.39 | -22.06 |
| Per capita | * | -1.23 | -1.14 | -2.21 |
| **Net monetary benefit (Societal costs; 1xGDP) (€)** | | | | |
| Millions | * | -23.17 | -11.39 | -32.9 |
| Per capita | * | -2.32 | -1.14 | -3.30 |
| **Marche** | | | | |
| **Payer costs (€)** | | | | |
| Millions | 23.08 | 24.30 | 24.58 | 25.58 |
| Per capita | 15.41 | 16.22 | 16.40 | 17.07 |
| **Societal costs (€)** | | | | |
| Millions | 50.87 | 53.55 | 52.37 | 54.83 |
| Per capita | 33.95 | 35.74 | 34.95 | 36.59 |
| **QALYs lost** | | | | |
| Total | 491.72 | 507.59 | 491.72 | 507.59 |
| Per 100,000 | 32.81 | 33.87 | 32.81 | 33.87 |
| **Net monetary benefit (Payer costs; 1xGDP) (€)** | | | | |
| Millions | * | -1.64 | -1.50 | -2.92 |
| Per capita | * | -1.09 | -1.00 | -1.95 |
| **Net monetary benefit (Societal costs; 1xGDP) (€)** | | | | |
| Millions | * | -3.11 | -1.5 | -4.38 |
| Per capita | * | -2.07 | -1.00 | -2.92 |
| **Molise** | | | | |
| **Payer costs (€)** | | | | |
| Millions | 4.07 | 4.31 | 4.34 | 4.53 |
| Per capita | 13.86 | 14.64 | 14.77 | 15.42 |
| **Societal costs (€)** | | | | |
| Millions | 9.31 | 9.88 | 9.58 | 10.11 |
| Per capita | 31.64 | 33.58 | 32.55 | 34.36 |
| **QALYs lost** | | | | |
| Total | 90.16 | 93.39 | 90.16 | 93.39 |
| Per 100,000 | 30.63 | 31.73 | 30.63 | 31.73 |
| **Net monetary benefit (Payer costs; 1xGDP) (€)** | | | | |
| Millions | * | -0.32 | -0.27 | -0.55 |
| Per capita | * | -1.08 | -0.91 | -1.86 |
| **Net monetary benefit (Societal costs; 1xGDP) (€)** | | | | |
| Millions | * | -0.66 | -0.27 | -0.89 |
| Per capita | * | -2.23 | -0.91 | -3.01 |
| **Piemonte** | | | | |
| **Payer costs (€)** | | | | |
| Millions | 64.95 | 68.32 | 69.17 | 71.94 |
| Per capita | 15.19 | 15.98 | 16.18 | 16.82 |
| **Societal costs (€)** | | | | |
| Millions | 144.39 | 151.85 | 148.61 | 155.47 |
| Per capita | 33.77 | 35.52 | 34.76 | 36.36 |
| **QALYs lost** | | | | |
| Total | 1423.30 | 1466.62 | 1423.30 | 1466.62 |
| Per 100,000 | 33.29 | 34.30 | 33.29 | 34.30 |
| **Net monetary benefit (Payer costs; 1xGDP) (€)** | | | | |
| Millions | * | -4.54 | -4.23 | -8.15 |
| Per capita | * | -1.06 | -0.99 | -1.91 |
| **Net monetary benefit (Societal costs; 1xGDP) (€)** | | | | |
| Millions | * | -8.63 | -4.23 | -12.24 |
| Per capita | * | -2.02 | -0.99 | -2.86 |
| **Prov. Auton. Bolzano** | | | | |
| **Payer costs (€)** | | | | |
| Millions | 11.00 | 11.70 | 11.76 | 12.35 |
| Per capita | 20.57 | 21.88 | 21.98 | 23.09 |
| **Societal costs (€)** | | | | |
| Millions | 22.97 | 24.85 | 23.73 | 25.50 |
| Per capita | 42.94 | 46.46 | 44.36 | 47.67 |
| **QALYs lost** | | | | |
| Total | 209.88 | 223.49 | 209.88 | 223.49 |
| Per 100,000 | 39.23 | 41.78 | 39.23 | 41.78 |
| **Net monetary benefit (Payer costs; 1xGDP) (€)** | | | | |
| Millions | * | -1.07 | -0.76 | -1.72 |
| Per capita | * | -1.99 | -1.41476 | -3.20274 |
| **Net monetary benefit (Societal costs; 1xGDP) (€)** | | | | |
| Millions | * | -2.25 | -0.76 | -2.9 |
| Per capita | * | -4.20 | -1.41 | -5.41 |
| **Prov. Auton. Trento** | | | | |
| **Payer costs (€)** | | | | |
| Millions | 9.82 | 10.37 | 10.50 | 10.95 |
| Per capita | 18.12 | 19.13 | 19.37 | 20.20 |
| **Societal costs (€)** | | | | |
| Millions | 19.42 | 20.62 | 20.10 | 21.20 |
| Per capita | 35.83 | 38.04 | 37.08 | 39.11 |
| **QALYs lost** | | | | |
| Total | 168.89 | 176.13 | 168.89 | 176.13 |
| Per 100,000 | 31.15 | 32.48 | 31.15 | 32.48 |
| **Net monetary benefit (Payer costs; 1xGDP) (€)** | | | | |
| Millions | * | -0.75 | -0.68 | -1.33 |
| Per capita | * | -1.37 | -1.25 | -2.44 |
| **Net monetary benefit (Societal costs; 1xGDP) (€)** | | | | |
| Millions | * | -1.4 | -0.68 | -1.98 |
| Per capita | * | -2.57 | -1.25 | -3.64 |
| **Puglia** | | | | |
| **Payer costs (€)** | | | | |
| Millions | 60.99 | 64.60 | 65.43 | 68.41 |
| Per capita | 15.50 | 16.42 | 16.63 | 17.39 |
| **Societal costs (€)** | | | | |
| Millions | 113.15 | 121.25 | 117.60 | 125.05 |
| Per capita | 28.76 | 30.82 | 29.89 | 31.79 |
| **QALYs lost** | | | | |
| Total | 926.92 | 970.38 | 926.92 | 970.38 |
| Per 100,000 | 23.56 | 24.66 | 23.56 | 24.66 |
| **Net monetary benefit (Payer costs; 1xGDP) (€)** | | | | |
| Millions | * | -4.78 | -4.45 | -8.58 |
| Per capita | * | -1.21 | -1.13 | -2.18 |
| **Net monetary benefit (Societal costs; 1xGDP) (€)** | | | | |
| Millions | * | -9.26 | -4.45 | -13.06 |
| Per capita | * | -2.35 | -1.13 | -3.32 |
| **Sardegna** | | | | |
| **Payer costs (€)** | | | | |
| Millions | 21.1 | 22.21 | 22.47 | 23.38 |
| Per capita | 13.27 | 13.97 | 14.13 | 14.70 |
| **Societal costs (€)** | | | | |
| Millions | 46.88 | 49.24 | 48.24 | 50.41 |
| Per capita | 29.48 | 30.97 | 30.34 | 31.7 |
| **QALYs lost** | | | | |
| Total | 456.93 | 469.94 | 456.93 | 469.94 |
| Per 100,000 | 28.73 | 29.55 | 28.73 | 29.55 |
| **Net monetary benefit (Payer costs; 1xGDP) (€)** | | | | |
| Millions | * | -1.46 | -1.37 | -2.63 |
| Per capita | * | -0.92 | -0.86 | -1.65 |
| **Net monetary benefit (Societal costs; 1xGDP) (€)** | | | | |
| Millions | * | -2.72 | -1.37 | -3.89 |
| Per capita | * | -1.71 | -0.86 | -2.44 |
| **Sicilia** | | | | |
| **Payer costs (€)** | | | | |
| Millions | 77.65 | 83.14 | 83.69 | 88.31 |
| Per capita | 16.06 | 17.20 | 17.31 | 18.27 |
| **Societal costs (€)** | | | | |
| Millions | 150.39 | 166.05 | 156.43 | 171.21 |
| Per capita | 31.11 | 34.35 | 32.36 | 35.42 |
| **QALYs lost** | | | | |
| Total | 1190.08 | 1273.76 | 1190.08 | 1273.76 |
| Per 100,000 | 24.62 | 26.35 | 24.62 | 26.35 |
| **Net monetary benefit (Payer costs; 1xGDP) (€)** | | | | |
| Millions | * | -7.73 | -6.05 | -12.90 |
| Per capita | * | -1.60 | -1.25 | -2.67 |
| **Net monetary benefit (Societal costs; 1xGDP) (€)** | | | | |
| Millions | * | -17.90 | -6.05 | -23.07 |
| Per capita | * | -3.70 | -1.25 | -4.77 |
| **Toscana** | | | | |
| **Payer costs (€)** | | | | |
| Millions | 53.20 | 56.14 | 56.83 | 59.25 |
| Per capita | 14.40 | 15.20 | 15.39 | 16.04 |
| **Societal costs (€)** | | | | |
| Millions | 108.39 | 114.74 | 112.02 | 117.85 |
| Per capita | 29.35 | 31.07 | 30.33 | 31.91 |
| **QALYs lost** | | | | |
| Total | 982.23 | 1017.32 | 982.23 | 1017.32 |
| Per 100,000 | 26.59 | 27.54 | 26.59 | 27.54 |
| **Net monetary benefit (Payer costs; 1xGDP) (€)** | | | | |
| Millions | * | -3.89 | -3.64 | -7.00 |
| Per capita | * | -1.05 | -0.98 | -1.89 |
| **Net monetary benefit (Societal costs; 1xGDP) (€)** | | | | |
| Millions | * | -7.30 | -3.64 | -10.41 |
| Per capita | * | -1.97 | -0.98 | -2.82 |
| **Umbria** | | | | |
| **Payer costs (€)** | | | | |
| Millions | 13.34 | 14.03 | 14.21 | 14.78 |
| Per capita | 15.41 | 16.21 | 16.43 | 17.08 |
| **Societal costs (€)** | | | | |
| Millions | 28.79 | 30.26 | 29.67 | 31.01 |
| Per capita | 33.27 | 34.96 | 34.28 | 35.83 |
| **QALYs lost** | | | | |
| Total | 278.29 | 286.63 | 278.29 | 286.63 |
| Per 100,000 | 32.15 | 33.11 | 32.15 | 33.11 |
| **Net monetary benefit (Payer costs; 1xGDP) (€)** | | | | |
| Millions | * | -0.92 | -0.88 | -1.67 |
| Per capita | * | -1.06 | -1.01 | -1.92 |
| **Net monetary benefit (Societal costs; 1xGDP) (€)** | | | | |
| Millions | * | -1.69 | -0.88 | -2.44 |
| Per capita | * | -1.95 | -1.01 | -2.81 |
| **Valle d'Aosta** | | | | |
| **Payer costs (€)** | | | | |
| Millions | 1.93 | 2.03 | 2.05 | 2.14 |
| Per capita | 15.57 | 16.4 | 16.59 | 17.27 |
| **Societal costs (€)** | | | | |
| Millions | 4.3 | 4.54 | 4.43 | 4.64 |
| Per capita | 34.68 | 36.58 | 35.70 | 37.45 |
| **QALYs lost** | | | | |
| Total | 42.49 | 43.90 | 42.49 | 43.90 |
| Per 100,000 | 34.23 | 35.37 | 34.23 | 35.37 |
| **Net monetary benefit (Payer costs; 1xGDP) (€)** | | | | |
| Millions | * | -0.15 | -0.13 | -0.25 |
| Per capita | * | -1.13 | -1.02 | -2.00 |
| **Net monetary benefit (Societal costs; 1xGDP) (€)** | | | | |
| Millions | * | -0.28 | -0.13 | -0.39 |
| Per capita | * | -2.21 | -1.02 | -3.08 |
| **Veneto** | | | | |
| **Payer costs (€)** | | | | |
| Millions | 74.89 | 79.13 | 80.20 | 83.67 |
| Per capita | 15.37 | 16.24 | 16.46 | 17.18 |
| **Societal costs (€)** | | | | |
| Millions | 138.22 | 147.05 | 143.53 | 151.59 |
| Per capita | 28.38 | 30.19 | 29.47 | 31.13 |
| **QALYs lost** | | | | |
| Total | 1161.91 | 1210.68 | 1161.91 | 1210.68 |
| Per 100,000 | 23.85 | 24.86 | 23.85 | 24.86 |
| **Net monetary benefit (Payer costs; 1xGDP) (€)** | | | | |
| Millions | * | -5.55 | -5.31 | -10.09 |
| Per capita | * | -1.14 | -1.09 | -2.07 |
| **Net monetary benefit (Societal costs; 1xGDP)** **(€)** | | | | |
| Millions | * | -10.14 | -5.31 | -14.68 |
| Per capita | * | -2.08 | -1.09 | -3.01 |

*Reference Strategy

**Figure S2.12: Deterministic and probabilistic sensitivity analyses.**


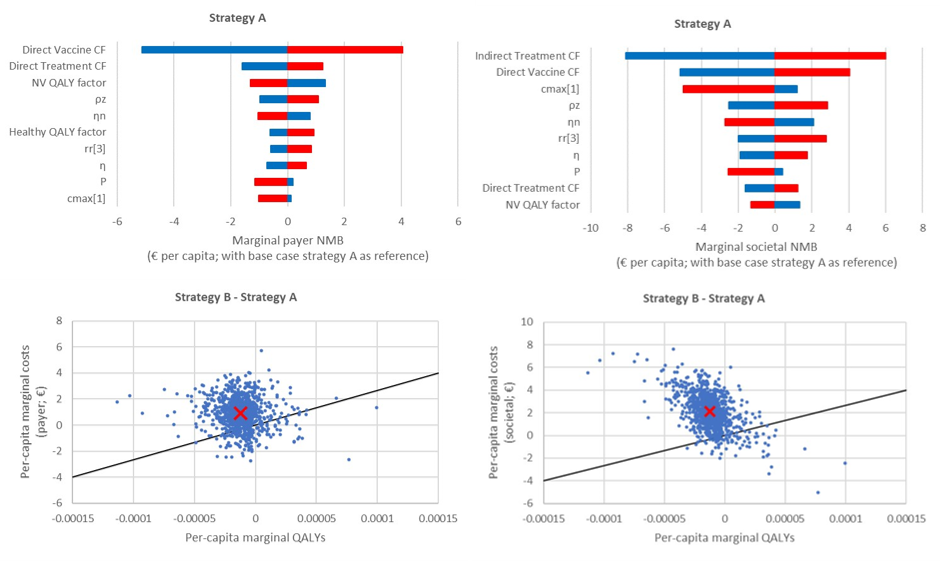


**Note 1**: (Top) Deterministic sensitivity analysis for marginal payer (left) and societal (right) net monetary benefit (NMB). (Blue bars) Result from parameter upper bound. (Red bars) Result from parameter lower bound.

**Note 2**: (Bottom) Probabilistic sensitivity analysis comparing marginal costs versus marginal QALYs gained for strategy B with respect to strategy A for the payer (left) and societal (right) perspective. (Black line) Willingness-to-pay threshold (1x GDP = 26,700 €). (Blue dots) Results from parameter realizations. (Red ex) Base case result.

**Abbreviations:** ρz: the relative infectiousness of HZ; ηn: duration of HZ outbreaks; cmax[1]: *vcr_equilibrium_1*$;$rr[3]: relative risk for ≥20-year-olds; π and η: reactivation rate parameter for “bathtub function” ; P: Vaccine success rate (1-Vaccine failure rate) of second dose.

# **References**

| [1] | A. De Donno, K. P, M. Guido, M. C. Rota, A. Bella, G. Brignole, S. Lupi, A. Idolo, A. Stefanati, M. Del Manso and G. Gabutti, “Has VZV epidemiology changed in Italy? Results of a seroprevalence study,” *Human Vaccines & Immunotherapeutics,* vol. 13, no. 2, pp. 385-90, 2017. |
| --- | --- |
| [2] | G. Gabutti, M. C. Rota, M. Guido, A. De Donno, A. Bella, M. L. Ciofi degli Atti and P. Crovari, “The epidemiology of varicella zoster virus infection in Italy,” *BMC Public Health,* no. 1, pp. 1-9, 2008. |
| [3] | L. E. Gialloreti, M. Merito, P. Pezzotti, L. Naldi, A. Gatti, M. Beillat, L. Serradell, R. di Marzo and A. Volpi, “Epidemiology and economic burden of herpes zoster and post-herpetic neuralgia in Italy: a retrospective, population-based study.,” *BMC Infectious Diseases,* vol. 10, no. 1, 2010. |
| [4] | C. Alicino, C. Trucchi, C. Paganino, I. Barberis, S. Boccalini, D. Martinelli, B. Pellizzari, A. Bechini, A. Orsi, P. Bonanni and R. Prato, “Incidence of herpes zoster and post-herpetic neuralgia in Italy: results from a 3-years population-based study,” *Human Vaccines & Immunotherapeutics,* vol. 13, no. 2, pp. 399-404, 2017. |
| [5] | C. Trucchi, G. Gabutti, M. C. Rota and A. Bella, “Burden of varicella in Italy, 2001–2010: analysis of data from multiple sources and assessment of universal vaccination impact in three pilot regions.,” *Journal of Medical Microbiology,* vol. 64, no. 11, pp. 1387-94, 2015 Nov. |
| [6] | A. Bechini, S. Boccalini, V. Baldo, S. Cocchio, P. Castiglia, T. Gallo, S. Giuffrida, F. Locuratolo, S. Tafuri, D. Martinelli, R. Prato, E. Amodio, F. Vitale and P. Bonanni, “Impact of universal varicella vaccination against varicella in Italy: Experiences from eight Italian Regions,” *Human Vaccines & Immunotherapeutics,* vol. 11, no. 1, pp. 63-71, 2015. |
| [7] | M. Marin, M. Marti, A. Kambhampati, S. M. Jeram and J. F. Seward, “Global varicella vaccine effectiveness: a meta-analysis,” *Pediatrics,* vol. 137, no. 3, 2016. |
| [8] | M. Riera-Montes, K. Bollaerts, U. Heininger, N. Hens, G. Gabutti, A. Gil, B. Nozad, G. Mirinaviciute, E. Flem, A. Souverain, T. Vertraeten and S. Hartwig, “Estimation of the burden of varicella in Europe before the introduction of universal childhood immunization,” *BMC Infectious Diseases,* vol. 17, no. 1, p. 353, 2017. |
| [9] | V. Baldo, T. Baldovin, F. Russo, M. Busana, C. Piovesan, G. Bordignon, A. Giliberti and R. Trivello, “Varicella: epidemiological aspects and vaccination coverage in the,” *BMC Infectious Diseases,* vol. 9, p. 150, 2009. |
| [10] | M. Ciofi Degli Atti, M. Rota, D. Mandolini, A. Bella, G. Gabutti, P. Crovari and S. Salmaso, “Assessment of varicella underreporting in Italy,” *Epidemiololy & Infection,* vol. 128, pp. 479-484, 2002. |
